# Comparative effectiveness of lower body positive pressure and traditional treadmill training on adults with mild balance impairment

**DOI:** 10.3389/fragi.2025.1645026

**Published:** 2025-10-22

**Authors:** Hina Shafi, Waqar Ahmed Awan, Sharon Olsen, Furqan Ahmed Siddiqi, Usman Rashid, Imran Khan Niazi

**Affiliations:** ^1^ Riphah College of Rehabilitation and Allied Health Sciences, Riphah International University, Islamabad, Pakistan; ^2^ Foundation Institute of Rehabilitation Sciences, Foundation University, Islamabad, Pakistan; ^3^ Health and Rehabilitation Research Institute, Faculty of Health and Environmental Sciences, AUT University, Auckland, New Zealand; ^4^ Centre for Chiropractic Research, New Zealand College of Chiropractic, Auckland, New Zealand; ^5^ Centre for Sensory-Motor Interaction (SMI), Department of Health Science and Technology, Aalborg University, Aalborg, Denmark

**Keywords:** adults with mild balance impairment, anti-gravity treadmill, balance, lower bodypositive pressure, mobility, treadmill

## Abstract

**Background:**

Treadmill training and body-weight supported treadmill training are effective for improving gait and balance in various populations. Lower-body positive pressure (PP) treadmill training uses positive air pressure to support body weight, potentially offering advantages over traditional treadmill training by reducing joint impact and allowing longer sessions. However, no studies have directly compared PP treadmill training with traditional treadmill training in adults with mild balance impairment.

**Method:**

In this three-armed parallel design randomised controlled trial, 72 adults were randomly assigned to: i) PP treadmill training with 20% bodyweight support (PP-BWS), ii) PP treadmill training without bodyweight support (PP-noBWS), and iii) traditional treadmill training without bodyweight support (TT). Participants in all three groups completed 25 min of treadmill training, three times per week, for 8 weeks. Outcomes included the Berg Balance Scale (BBS), Timed Up and Go (TUG), Functional Reach Test (FRT), and postural sway and gait measured with smartphone accelerometry and force plates. Outcomes were collected at baseline, at the end of the 2nd, 4th, 6th, and 8th week, and follow-up data were collected in the 10th week. Data were analysed using linear mixed-effects models, with multiple-imputation sensitivity analyses.

**Results:**

All interventions resulted in significant within-group improvements in balance and mobility measured with the BBS, TUG and FRT. For balance measures, the primary analysis revealed a group by time interaction (p = 0.003) for the BBS, favouring traditional treadmill training and PP-noBWS at week 10, but no between-group differences for the FRT. TUG measures of functional mobility showed a significant group by time interaction (p = 0.028), initially favouring novel PP-BWS, but there were no between-group differences after week 4. This aligned with smartphone accelerometry outcomes, which showed no between-group differences for comfortable walking speed and gait symmetry. Between-group differences in standing postural sway did not consistently favour one group. Due to a large dropout rate at follow-up, a sensitivity analysis was completed; this confirmed the significant within-group effects on balance and mobility at week 10, but between-group differences in balance were no longer statistically significant.

**Conclusion:**

All treadmill interventions led to significant within-group improvements in balance and mobility over the 10-week period. The initial analysis suggested treadmill interventions without body weight support, traditional treadmill training and PP-noBWS, demonstrated larger improvements in balance at week 10, but between-group differences were not sustained after accounting for dropout rates in the sensitivity analysis. This may suggest that the altered gait mechanics and reduced sensory input during PP treadmill training with bodyweight support may limit the improvements in balance that accompany treadmill training.

## 1 Introduction

Aging is associated with numerous changes in the neuromuscular system (Rezaei et al., 2024). These include increased unsteadiness during unperturbed upright standing (Osoba et al., 2019) and a general decline in balance responses during standing and walking (Osoba et al., 2019; Rezaei et al., 2024). Balance responses enable a person to maintain a posture (e.g., sitting or standing), move between postures, and avoid a fall when reacting to an external disturbance (McCormick and Vasilaki, 2018). Thus, age-related changes in balance are associated with an increased risk of falls and associated injuries (Henry and Baudry, 2019). While falls are one potential consequence of impaired balance and mobility, the broader impact includes decreased independence and participation in daily activities, reduced quality of life, increased reliance on caregivers, and significant healthcare resource use (Haddad et al., 2019; Chittrakul et al., 2020; Abell et al., 2021; Miller et al., 2025). Addressing age-related physical decline is crucial for improving functional capacity and wellbeing in older populations (Schoene et al., 2019).

A range of age-related impairments that increase fall risk can be targeted with exercise programs. These include the decline in muscle strength, decreased functional mobility, reduced balance, and altered gait characteristics, such as decreased speed (Orange et al., 2019). Physical exercise targeting these impairments can reduce falls and fall-related effects in older adults (Rodrigues et al., 2022). Exercise can involve different modalities, and can target various aspects of neuromuscular control such as balance strategies (Horlings et al., 2008; Taube et al., 2008; Cadore et al., 2013), ankle mobility (Allum et al., 2002; Gajdosik et al., 2005; Ema et al., 2017), sensory-motor processing, muscle strength (Peterson et al., 2010; Chen N. et al., 2021), walking function and endurance (Horlings et al., 2008; Taube et al., 2008; Cadore et al., 2013).

A common modality used to improve balance and mobility is treadmill training, with or without body weight support (BWS). This rehabilitation approach has been studied in various older adult populations (Rieger et al., 2024; Zafer et al., 2024). However, the majority of research has focused on neurological populations. Systematic review evidence has shown clear benefits for postural balance, gait pattern and speed, sitting to standing transfers, and lower limb strength in people with PD (Ganesan et al., 2015; Mehrholz et al., 2015; Bishnoi et al., 2022) and stroke (Schindl et al., 2000; Lindquist et al., 2007; Hesse, 2008; Bishnoi et al., 2022).

A small body of research has studied treadmill training as a component of exercise programs for adults with balance impairment and falls (Sauvage et al., 1992; Nowalk et al., 2001; Tsaih et al., 2012). While positive results of treadmill training have been demonstrated in populations such as PD (Ganesan et al., 2015; Mehrholz et al., 2015; Bishnoi et al., 2022) and stroke (Schindl et al., 2000; Lindquist et al., 2007; Hesse, 2008; Bishnoi et al., 2022), evidence for its effectiveness as a stand-alone intervention in older adults remains limited (Pirouzi et al., 2014; Pereira et al., 2020). Traditional treadmill training (TT) in isolation has improved balance and functional mobility in institutionalised older adults compared with a no-exercise control (Pereira et al., 2020). Treadmill training, both with and without perturbations, can improve balance, gait performance, and reduce concerns about falling in older adults at risk of falls; nevertheless, perturbation-based treadmill training is superior to conventional treadmill training. (Rieger et al., 2024). The main disadvantage of perturbation-based treadmills is that they are less safe, discomfort and have higher source requirements (McCrum et al., 2022). Treadmill training is theorised to improve functional independence by enhancing cardiovascular endurance (MacKay-Lyons, 2012; Shulman et al., 2013), gait parameters (Bishnoi et al., 2022), lower limb strength (Shulman et al., 2013), and postural control (Pereira et al., 2020; Zafer et al., 2024).

Traditional TT is often implemented with BWS using a harness system to optimise safety and reduce the mechanical support required from staff (Mehrholz et al., 2017). These harness systems, though effective, can cause discomfort and impede circulation due to the straps required on the torso and lower extremities. Another approach to unweight the body is through water immersion, but water-based TT may alter gait timing, joint kinematics, and muscle activity due to the drag forces of the water acting in opposition to movement (Hall et al., 2004; Raghu et al., 2021; Mohammadi Momen et al., 2024). More recently, another method of providing body weight support has been developed that uses negative air pressure applied to the lower body to provide BWS. This method is termed lower body positive pressure (PP) (Lazaro, 2020). An inflatable bag is included in this system. The patient is dressed in neoprene shorts that are zipped up within the bag. The air pressure in the bag, which acts as a lifting force on the body, determines how much body weight is supported. This chamber is part of the treadmill structure and creates a sealed environment below the waist (AlterG, 2024). The air pressure is equally distributed across the lower body, minimising the pressure points found in traditional BWS systems (Abdelaal and El-Shamy, 2022).

Lower body PP has shown promising effects on gait, physiological, and functional outcomes in various populations, including individuals with cerebral palsy (Alwhaibi et al., 2022), stroke (Almutairi S., 2023; Almutairi S. M., 2023), well-trained male athletes (Farina et al., 2017), healthy men (Stucky et al., 2018), and in a pilot study of community-dwelling older adults (Lazaro, 2020). In the latter pilot study of five older adults, 8 weeks of lower-body PP treadmill training with 20% BWS resulted in significantly improved lower extremity strength, while walking speed and balance showed improvements that did not reach statistical significance (Lazaro, 2020). These findings suggest that lower-body PP training could be beneficial in enhancing balance and mobility and reducing the risk of falls in older adult populations. By altering neuromuscular and physiological load during gait practice (Pereira et al., 2020), lower-body PP training may decrease fatigue and injury risk, creating a safer and more tolerable environment for older adults to engage in higher volumes of repetitive practice. This may enable more sustained training at sufficient intensity to promote functional improvements, while minimising overexertion and adverse events that might otherwise limit participation or progression (Pereira et al., 2020; Zafer et al., 2024). However, no research has compared lower-body PP treadmill training with traditional gait training methods to evaluate its relative effectiveness, particularly in improving static and dynamic balance, and functional mobility in adults with mild balance impairment. Larger randomised control studies with sufficient sample sizes are needed to better understand the potential effects of the PP treadmill intervention (Almutairi S. M., 2023). Therefore, this study investigated the effect of lower-body PP treadmill training on balance and mobility in adults with mild balance impairment, compared with two comparison interventions: PP treadmill training without BWS, and traditional treadmill training. It was hypothesised that lower-body PP treadmill training with body weight support would lead to greater improvements in balance and functional mobility compared with the two treadmill training interventions without positive pressure body weight support.

## 2 Methods

### 2.1 Design and setting

This was a parallel-group, randomized controlled trial (RCT). The study was conducted at the Foundation Institute of Rehabilitation Sciences, Foundation University, Islamabad. The study received approval from the ethical review committees of Riphah International University, Pakistan (Riphah/RCRS/REC/Letter-0011961- [7 November 2020]) and the Foundation University Islamabad (FF/FUMC/215-45 Phy20-[8 October 2020]). The study was registered with the National Institutes of Health ClinicalTrials.gov clinical trial registry (NCT04636645).

### 2.2 Study participants

The participants were Pakistani adults with mild balance impairment. The sample size was calculated based on an effect size of the Berg balance scale (BBS) of 3.3 (Donoghue and Stokes, 2009) and a test-retest correlation of 0.97. Because our study used linear mixed-effects models (LMMs) with repeated measures and multiple covariates, a simulation-based approach in R (42,000 tests) was employed, as traditional tools such as G*Power are less suited to this design. The simulation indicated that 20 participants per group (total N = 60) would be sufficient, allowing for dropout and variability expected in this population. The inclusion criteria were: aged 50 years or above, BBS score ranging from 46 to 54 (indicating mild balance impairment without the use of aids) (Donoghue and Stokes, 2009) and the ability to follow instructions in Urdu. Participants were classified as adults with mild balance impairment using an age threshold of ≥50 years, consistent with rehabilitation research in Pakistan and other low- and middle-income countries, where lower life expectancy (approximately 67 years) (World Health Organization and Unicef, 2022), socioeconomic challenges, nutritional deficiencies, and limited healthcare access contribute to the earlier onset of age-related functional declines such as frailty, reduced mobility, and mild balance impairment, as targeted in this study (World Health Organization and Unicef, 2022). Participants were excluded if they had a history of neurological conditions or musculoskeletal injuries within the past year, pain with ambulation, dizziness when standing or walking, any contraindications to lower body PP or treadmill training (such as cardiovascular conditions like uncontrolled hypertension, arrhythmias, or recent myocardial infarction), or severe diagnosed osteoporosis that would contraindicate loading of specific body regions (such as the abdominal region, hip, or pelvis). Before participating, all individuals provided written informed consent following the Declaration of Helsinki guidelines. All participants were also informed that the study aimed to compare three different types of treadmill training on balance, without disclosing specific expectations about which approach might be superior.

### 2.3 Randomisation

After consent and baseline assessments, participants were randomised into three intervention groups (PP-BWS, PP-noBWS, and TT) using Minimiser (Microsoft Corp., Redmond, WA). Allocation was based on the software output and implemented by a researcher not involved in assessments or data analysis. This process ensured allocation concealment, with outcome assessors and data analysts remaining blinded to group assignments.

### 2.4 Interventions

Participants completed their allocated intervention three times per week for eight consecutive weeks. Each session lasted 25 min. For all interventions, participants completed a warm-up at a self-selected comfortable speed for 5 min. Then the treadmill speed was increased until the participants self-reported a rating of perceived exertion (RPE) of 10–13 (Borg, 1982), indicating a “fairly light” to “somewhat hard” intensity, that was challenging enough to promote health benefits without being overwhelming or excessively strenuous (Elsawy and Higgins, 2010). Walking was maintained at this intensity for 25 min, up to a maximum speed of 3.1 mph, with a 0-degree incline. This ceiling speed and RPE range were chosen to prioritise participant safety, particularly for deconditioned adults (Pereira et al., 2020). If a participant felt excessively exerted, the speed was reduced or the session was stopped, depending on the severity of the exertion. Adherence to the 8-week treadmill training protocols (PP-BWS, PP-noBWS, TT) was monitored using session logs completed by trained staff, who supervised all sessions to ensure compliance with prescribed intensity, duration, and safety protocols. The specifics of each treadmill intervention are described below.

1. Lower body positive pressure (PP) with bodyweight support (PP-BWS): Participants were suspended on an anti-gravity treadmill (AlterG M320; AlterG™) with BWS set to 20%. To set this up, participants stepped into neoprene shorts, which were then zipped into a pressurized air chamber that surrounded the lower body. This chamber was part of the treadmill structure and created a sealed environment below the waist (AlterG, 2024). BWS was provided via the negative air pressure, which supported the person’s body weight at the waistband.
2. Lower body positive pressure (PP) without bodyweight support (PP-noBWS): Participants were set up in the same anti-gravity treadmill (AlterG M320; AlterG™) as the first intervention group, with the same neoprene shorts and inflatable bag, but without any negative air pressure inside the bag. Thus, they experienced a similar environment, with waistband support that would prevent a fall, but without the additional deweighting of body weight.
3. Traditional Treadmill Training (TT): Participants walked on a standard treadmill (Apollo^®^ model: Smart t9AC).

### 2.5 Outcome measures

The following assessments were conducted at baseline, at the end of weeks 2, 4, 6 and 8, and at week 10 for follow-up. Post-tests were scheduled 24–48 h after the final session before each assessment time. Assessments (BBS, TUG, FRT, G&B App, force plate) were conducted in a controlled indoor clinic (22–26 °C, consistent lighting) during morning sessions (8 a.m.–12 p.m.) to minimise diurnal and environmental variability.

#### 2.5.1 Berg balance scale (BBS)

Evaluation of functional balance was completed using the BBS, a valid and reliable (ICC = 0.986) (Berg et al., 1995; Newstead et al., 2005) tool for dynamic and static balance assessment. The BBS requires a measuring ruler, a stopwatch and two chairs and is considered safe and easy to administer to the elderly. The BBS takes almost 15 min to complete and consists of 14 items, where balance is assessed using an ordinal scale comprising 5 points that range from 0 to 4. High scores are indicative of superior balance, whereas low scores are suggestive of increased fall risk and poor balance (Berg et al., 1989). Scoring of 0–4 usually relies on the time required by a person to achieve a specific task (e.g., 8 times stepping on a small step) or time taken by a person to maintain a specific position (e.g., single leg stance or tandem stance). Scoring was completed via observation by a trained researcher (Siddiqi, 2019).

#### 2.5.2 Timed up and go test (TUG)

The TUG assesses balance and mobility by requiring purposeful movement of the base of support (BOS) and is commonly used to evaluate fall risk (Barry et al., 2014). It has excellent test-retest reliability (ICC = 0.97) (Steffen et al., 2002) in the older adult population. The TUG requires a normal standardized chair without back support. Each participant was instructed to stand up, walk as quickly as possible (but safely) to a 3-m mark, turn around, return to the chair, and sit back down. The test was conducted once, and the total duration was measured using a stopwatch. Participants were permitted to use an assistive device for this task, but devices were kept consistent between measurement time points.

#### 2.5.3 Functional reach test (FRT)

This FRT was used to assess limits of stability while reaching forward. The test and re-test reliability (ICC = 0.89) for FRT is excellent in community-dwelling older adults (Weiner et al., 1992). This assessment is carried out by calculating the maximum distance a person can reach forward in a fixed standing position. The participant kept their shoulder flexed at 90° and stood next to a meter scale on the wall. The position of the head of the 3rd metacarpal was noted, and the participant was instructed to lean forward as much as possible without stepping. At this point, the position of the head of the 3rd metacarpal was again noted, and the difference between the start and end points was recorded. Participants were given three trial sessions, and the mean of the last two trials was considered the actual value (Weiner et al., 1992).

#### 2.5.4 Gait and balance mobile application (G&B app)

The G&B system consists of the following three components: i) a standard smartphone (iPhone 7) which has embedded accelerometers, ii) a customized belt with a pocket to house the phone at the lumbosacral junction on the lower back (Allcare Ortho Core Stability Belt, Whiteley Allcare, Auckland, New Zealand), iii) and the Gait and Balance mobile application (G&B App). The protocol involves six different tasks, including 4 quiet stance tasks of 30 s each (standing barefoot with feet hip-width apart and arms by sides, on a firm surface with eyes open and closed, and on a compliant surface with eyes open and closed) and 2 gait tasks (normal walking, and walking with head turning). The compliant surface utilized was medium-density foam (50 cm × 28 cm × 5 cm, Diamond Foam, Lahore, Pakistan) (Rashid et al., 2022; Olsen et al., 2023; Shafi et al., 2023). At the onset of the study, the reliability and validity of this mobile application were tested in this adults with mild balance impairment population revealing a range of reliable and valid gait and balance parameters (Shafi et al., 2023).

#### 2.5.5 Force plate

Postural sway in quiet stance was also measured while standing on gold-standard force plate (Pasco Force plate, Perform Better Limited, Southam, United Kingdom) at a sampling rate of [20 Hz], under the same four conditions recorded with the G&B App. For the compliant surface, the foam was placed directly on the force plate. Placing foam directly on a force plate to create a compliant surface for balance testing is a common and accepted method (Carzoli and Enoka, 2023; Boonkerd et al., 2024).

### 2.6 Data processing

#### 2.6.1 Gait and balance app data

Three outcome measures, namely, postural stability, mediolateral (ML) stability, and anterior-posterior (AP) stability, were calculated from the 4 quiet stance tasks. Postural stability units were reported as the negative natural logarithm of acceleration (−ln [m/s/s]). Additionally, for the gait tasks, four outcome measures were recorded: mean walking speed (m/s), gait symmetry (periodicity index) (%), average step length (m), and average step time (s) (Rashid et al., 2022).

#### 2.6.2 Force plate data

Initially, a total of 22 outcomes were computed from the force plate data using the standard pipeline provided by the force plate software (PASCO Capstone 2.0). To reduce redundancy and identify a concise set of outcome domains, a factor analysis was performed to determine the number of factors that accounted for at least 90% of the variance in the force plate outcomes. Subsequently, a single representative outcome was selected from each factor to aid the simplicity of interpretation. The selected outcomes were Total Distance wandered, Total medial-lateral sway (ML Sway) and Total Anterior-Posterior (AP) sway.

#### 2.6.3 Important changes to the trial after commencement

To enhance feasibility and clarity while maintaining scientific rigor, we implemented a limited protocol refinement. The age eligibility threshold was revised from ≥55 to ≥50 years early in recruitment. This change was necessitated by COVID-19–related disruptions and a higher-than-anticipated comorbidity burden in the ≥55 cohort, which constrained accrual.

### 2.7 Statistical analysis

Data were analysed to evaluate the null hypothesis that there were no statistically significant differences between post-intervention outcomes in the three groups (after accounting for pre-intervention scores and pre-specified covariates) (De Boer et al., 2015). To facilitate the interpretation of change over time, the post-intervention outcomes were converted to change scores by subtracting from them the pre-intervention outcomes. At each time point, only the complete pre- and post-intervention outcome pairs were included in the primary intention-to-treat analysis models. The change scores were then regressed using linear mixed-effects models on pre-intervention scores, age, height, BMI, gender, group (TT, PP-BWS, PP-noBWS), time (week 2, 4, 6, 8, 10), and the interaction of group and time. For the G&B App and force plate outcomes, task type (firm/compliant surface) and task condition (eyes open/closed, head forwards/turning) were also included in the model, along with their interactions with group and time. Random intercepts for participants, and in some cases week-wise correlated random intercepts for participants, were also included in the model to ensure that the correlation structure of the repeated-measures in the dataset was adequately accounted for. The choice of the random effects structure was based on the minimisation of Akaike’s information criterion. The null hypothesis was tested with analysis of deviance, which checked for the statistical significance of the group variable in the model and its interaction with other variables. For statistically significant outcomes, the between-group marginal mean differences in change scores were estimated from the models. Their 95% confidence intervals were also reported. The within-group change scores were also reported at each post-intervention time point and tested against the null hypothesis that the change was zero. Benjamini-Hochberg adjustment was applied for pair-wise hypothesis tests. The statistical significance criterion was set as p-value <0.05. Cohen’s d effect sizes were also reported for statistically significant between-group differences. The robustness of the significant findings to data missingness was evaluated by conducting a secondary sensitivity analysis in which the missing values in the intention-to-treat dataset were imputed 50 times through multivariate chained equations and the primary analysis was repeated on the imputed datasets. All the covariates from the primary analysis were included in the imputation model. If a statistically significant between- or within-group effect from the primary analysis remained statistically significant in the secondary analysis, it was marked with an obelisk (†). Analysis was performed in the R environment for statistical computing using *lme4*, *mice*, *ggplot2*, *dplyr*, *car*, and *emmeans* (Bates et al., 2015; Wickham, 2016; Fox and Weisberg, 2018; R Core Team, 2019; Wickham et al., 2023) packages.

## 3 Results

### 3.1 Participant characteristics

**FIGURE 1:**
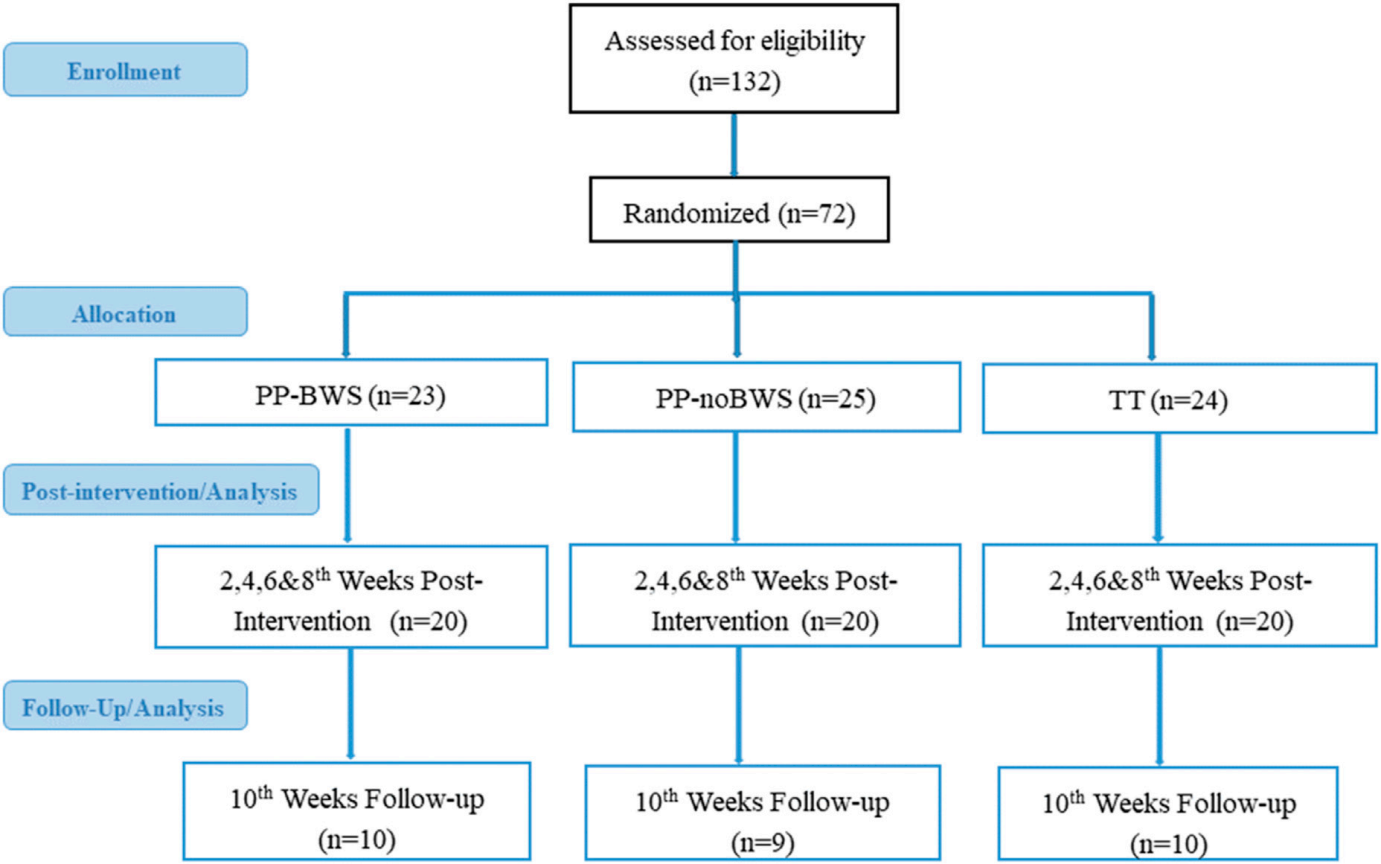
CONSORT flow chart showing flow of participants through the study. PP-BWS = Lower body positive pressure treadmill training with 20% bodyweight support; PP-noBWS = Lower body positive pressure treadmill training without bodyweight support; TT = traditional treadmill training without bodyweight support. Dropouts: PP-BWS: 3 after allocation, 1 at week 4 and 1 at week 8; PP-noBWS: 5 after allocation, 2 at week 6 and 1 at week 8; TT: 4 after allocation and no dropouts during intervention. The dropouts in all groups were due to transport issues.

The flow of participants through the study is shown in Figure 1. A total of 72 participants were enrolled and randomized to the three intervention groups. The dropout pattern showed: PP-BWS group lost 3 participants after allocation, 1 at week 4 and 1 at week 8, PP-noBWS group lost 5 participants after allocation, 2 at week 6 and 1 at week 8; and TT group lost 4 participants after allocation and no dropouts during intervention. Between weeks 8 and 10 there was further attrition across all arms, PP-BWS (10), PP-noBWS (9), and TT (10), leaving 29 participants at week-10 follow-up. The mean age of the participants was 55.9 years (SD 5.5), with a mean BMI of 29.0 (SD 5.4), and 42 were female (Table 1).

**TABLE 1:** Baseline characteristics.

Variable	PP-BWS	PP-noBWS	TT
Age (Year)	57.95 ± 6.32	54.65 ± 4.29	55.05 ± 5.45
BMI (kg/m^2^)	29.34 ± 4.48	28.63 ± 6.87	28.88 ± 4.84
Height (cm)	158.9 ± 10.25	165 ± 8.64	164.45 ± 9.15
Weight (kg)	73.83 ± 13.80	77.38 ± 16.78	77.755 ± 11.96
BBS (Score)	50.8 ± 2.39	52 ± 1.56	51.25 ± 1.33
FRT (cm)	8.77 ± 2.71	9.43 ± 2.41	10.64 ± 2.08
TUG (s)	12.16 ± 2.83	12.43 ± 2.29	13.34 ± 3.27

Comparison of baseline characteristics, Berg Balance Scale (BBS), Functional Reach Test (FRT), Timed Up and Go (TUG), age, BMI, height and weight, across three study groups: (i) PP, treadmill training with 20% body weight support (PP-BWS), (ii) PP, treadmill training without body weight support (PP-noBWS), and (iii) traditional treadmill training without body weight support (TT).

There were no significant differences between participant characteristics or clinical outcomes at baseline.

### 3.2 Effects of training on clinical outcomes

Clinical outcomes over the 10 weeks are shown in Table 1. There were significant within-group effects for all groups for the BBS, FRT and TUG. The results of ANOVA tests and post-hoc between-group differences for each outcome at each time point can be seen in Supplementary Table S1 (Supplementary Material 1).

#### 3.2.1 Berg balance scale

**FIGURE 2:**
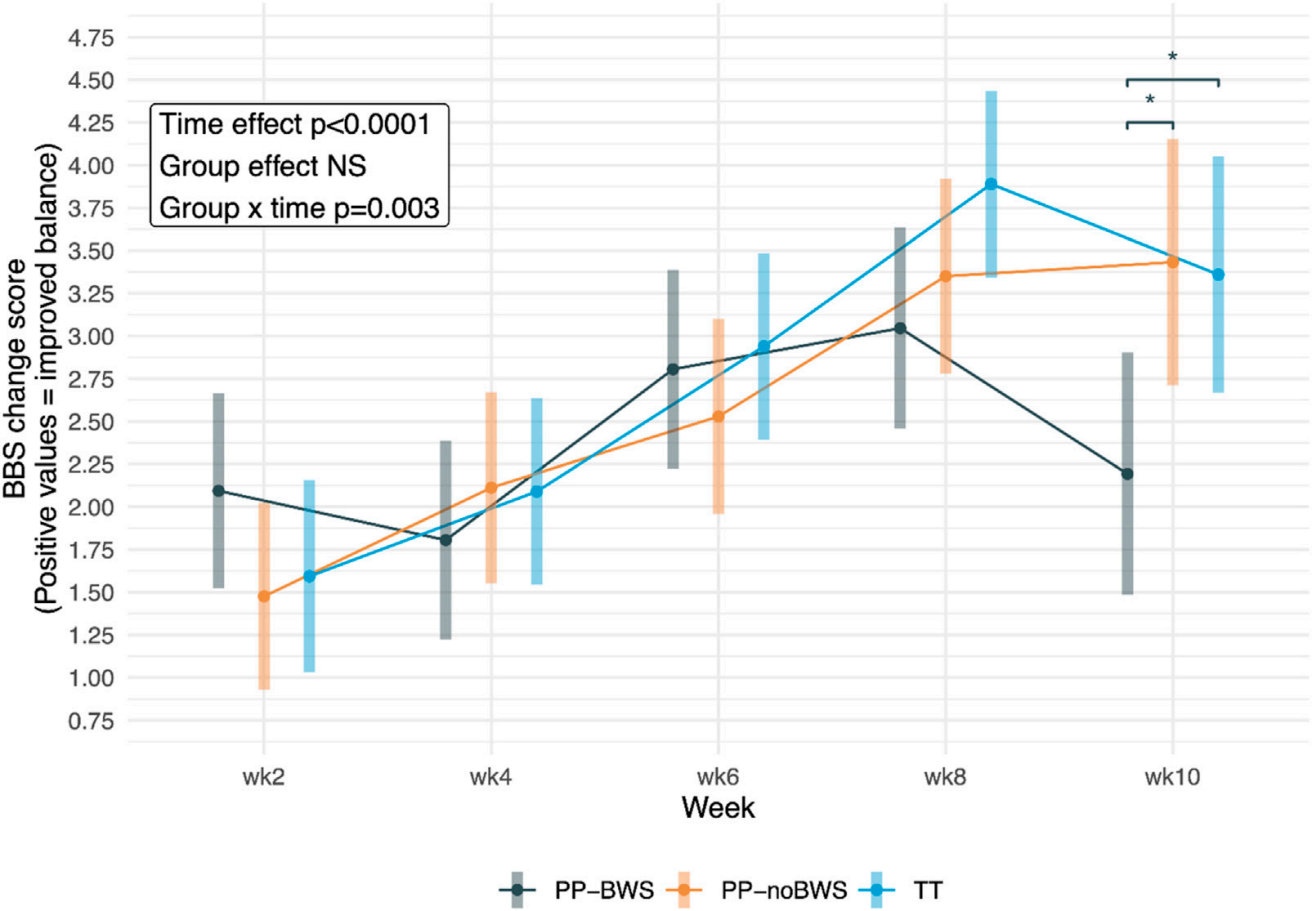
Change scores for Berg Balance Scale (BBS) in the three intervention groups after 2, 4, 6, and 8 weeks of intervention, and at the 10-week follow-up. Significant between-group differences are marked with *.

For the BBS, there were significant effects of time (p < 0.0001) and a group by time interaction (p = 0.003). Post hoc tests showed at week 10 (follow-up), both PP-noBWS and TT performed significantly better than PP-BWS (p = 0.027, SMD = −0.17 each). These differences are illustrated in Figure 2, which shows that TT (blue) exhibited the most substantial improvement up to week 8, but then declined by week 10. Whereas PP-noBWS demonstrated improvements up to week 8, and these were maintained at week 10. The between-group differences were not statistically significant in the sensitivity analysis. Whereas all the within-group differences remained statistically significant. The detailed analysis and results are provided in Supplementary Material 2.

#### 3.2.2 Functional reach test

**FIGURE 3:**
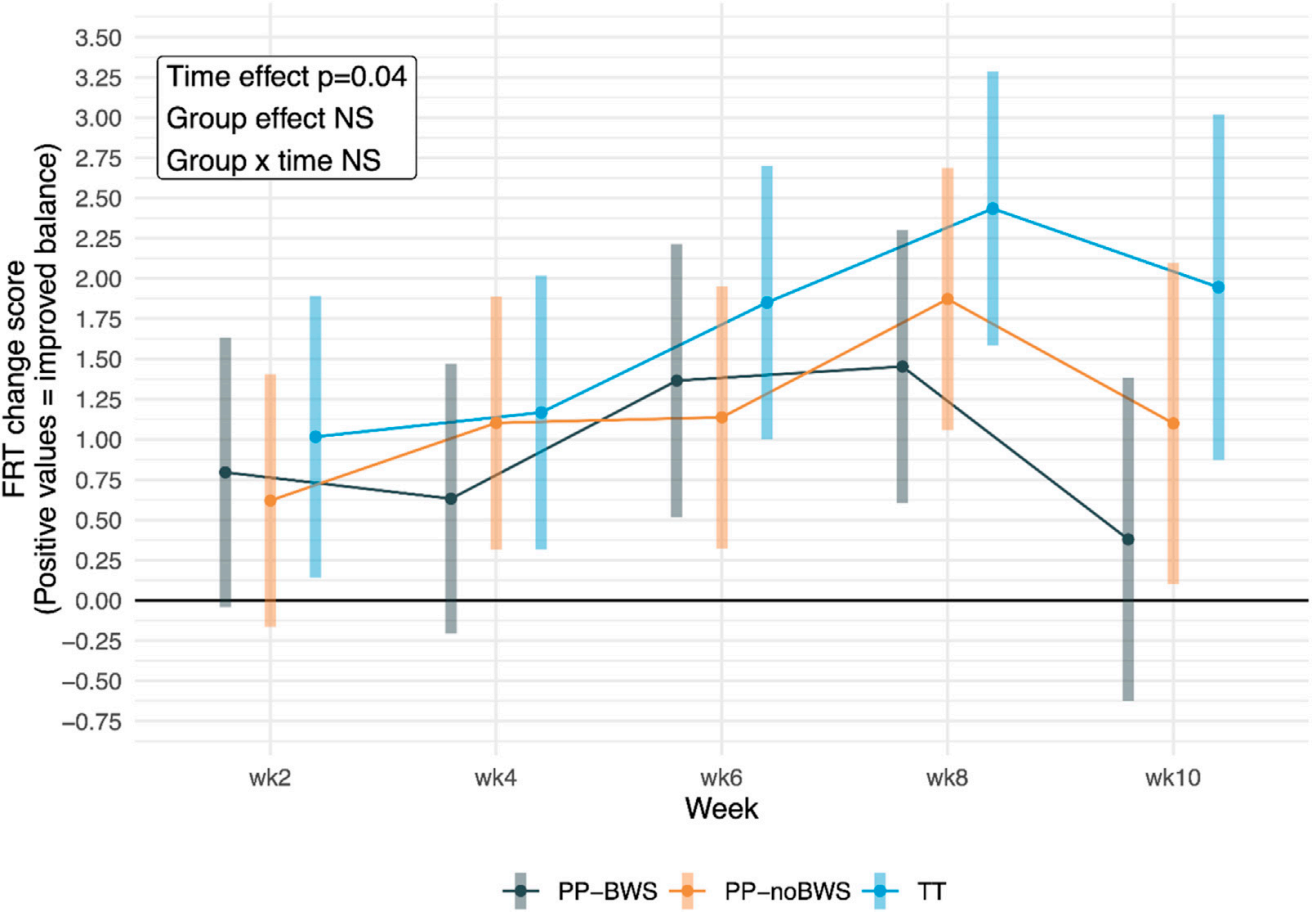
Change scores for Functional Reach Test (FRT) in the three intervention groups at 2, 4, 6, and 8 weeks of intervention, and at the 10-week follow-up.

Although there were no significant between-group differences, there was a significant effect of time (p = 0.04) (see within-group effects in Table 2), and the FRT change score plot in Figure 3 shows a trend of improvement up to week 8 in all groups, followed by a decline between weeks 8 and 10 across all three groups. The detailed analysis and results are provided in Supplementary Material 2.

**TABLE 2:** Clinical outcomes.

Outcome measures	Group	Baseline	Week 2	Week 4	Week 6	Week 8 (post)	Week 10 (follow up)
BBS (Score)	PP-BWS	50.8 ± 2.39	52.85 ± 1.63^a^ ^,^ ^b^	52.52 ± 1.89^a^ ^,^ ^b^	53.52 ± 1.38^a^ ^,^ ^b^	53.83 ± 1.85^a^ ^,^ ^b^	53.20 ± 2.20^a^ ^,^ ^b^
	PP-noBWS	52.0 ± 1.55	53.05 ± 1.63^a^ ^,^ ^b^	53.55 ± 1.29^a^ ^,^ ^b^	54.00 ± 1.54^a^ ^,^ ^b^	54.76 ± 1.34^a^ ^,^ ^b^	54.55 ± 1.58^a^ ^,^ ^b^
	TT	51.25 ± 1.33	52.77 ± 0.87^a^ ^,^ ^b^	53.25 ± 1.01^a^ ^,^ ^b^	54.10 ± 0.79^a^ ^,^ ^b^	55.05 ± 53.83^a^ ^,^ ^b^	54.60 ± 1.71^a^ ^,^ ^b^
FRT (cm)	PP-BWS	8.77 ± 2.71	9.52 ± 2.21	9.36 ± 2.65	10.13 ± 2.31^a^ ^,^ ^b^	10.22 + 2.35^a^ ^,^ ^b^	9.79 + 1.77
	PP-noBWS	9.43 ± 2.40	10.23 ± 2.06	10.60 ± 2.43^a^ ^,^ ^b^	10.40 ± 2.60^a^ ^,^ ^b^	11.12 + 2.22^a^ ^,^ ^b^	10.07 ± 2.22^a^ ^,^ ^b^
	TT	10.64 ± 2.08	11.14 ± 1.80^a^ ^,^ ^b^	11.18 ± 1.77^a^ ^,^ ^b^	11.82 ± 1.28^a^ ^,^ ^b^	12.45 + 1.26^a^ ^,^ ^b^	12.01 ± 1.52^a^ ^,^ ^b^
TUG (s)	PP-BWS	12.16 ± 2.83	11.20 ± 12.34^a^ ^,^ ^b^	11.02 ± 1.71^a^ ^,^ ^b^	11.00 ± 1.84^a^ ^,^ ^b^	11.00 + 1.66^a^ ^,^ ^b^	11.16 ± 2.09^a^ ^,^ ^b^
	PP-noBWS	12.42 ± 2.28	12.34 ± 2.52	12.13 ± 2.16	11.43 ± 1.91^a^ ^,^ ^b^	11.04 + 1.84^a^ ^,^ ^b^	11.28 ± 1.40^a^ ^,^ ^b^
	TT	13.34 ± 3.27	12.04 ± 2.42^a^ ^,^ ^b^	12.37 ± 2.37^a^ ^,^ ^b^	11.93 ± 1.79^a^ ^,^ ^b^	10.93 + 1.93^a^ ^,^ ^b^	11.94 ± 2.59^a^ ^,^ ^b^

^a^ Statistical significance detected in the primary analysis for within-group change from baseline.

^b^ Primary results remained statistically significant in the sensitivity analysis.

Comparison of clinical outcome measures, Berg Balance Scale (BBS), Functional Reach Test (FRT), and Timed Up and Go (TUG), across three study groups: (i) PP, treadmill training with 20% body weight support (PP-BWS), (ii) PP, treadmill training without body weight support (PP-noBWS), and (iii) traditional treadmill training without body weight support (TT).

#### 3.2.3 Time up and go (TUG)

**FIGURE 4:**
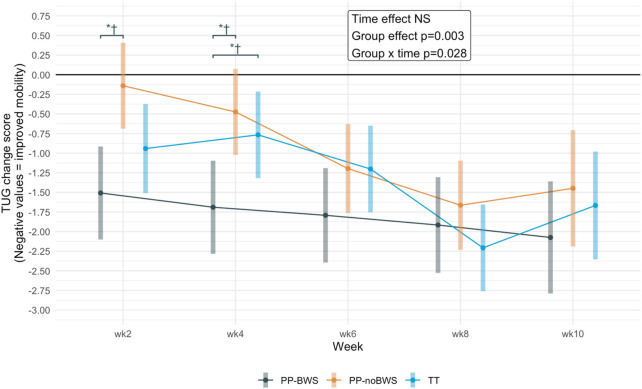
Change scores for the Timed Up and Go (TUG) in the three groups after 2, 4, 6, and 8 weeks of intervention, and at the 10-week follow-up. Significant between-group differences are marked with *, and the ones that are also significant in the sensitivity analysis are marked with †.

For the TUG, there was a significant effect of group (p = 0.002) and a significant group by time interaction (p = 0.028). Post hoc tests showed at weeks 2 and 4, there were statistically significant between-group differences when comparing PP-BWS, PP-noBWS and TT (p < 0.01), favouring PP-BWS; however, between-group differences were not apparent after week 4. These differences are illustrated in Figure 4. The sensitivity analysis upheld the statistical significance of these differences. The detailed analysis and results are provided in Supplementary Material 2.

#### 3.2.4 Force plate outcomes

For total distance wandered, *post hoc* tests found no between-group differences for the three easiest conditions: firm surface with eyes open, firm surface with eyes closed, and compliant surface with eyes open. However, for compliant surface with eyes closed, there was a between-group difference at week 4, but no differences after this (Supplementary Material S1). The statistical significance of this result was not upheld in the sensitivity analysis. The detailed analysis and results are provided in Supplementary Material 3.

**FIGURE 5:**
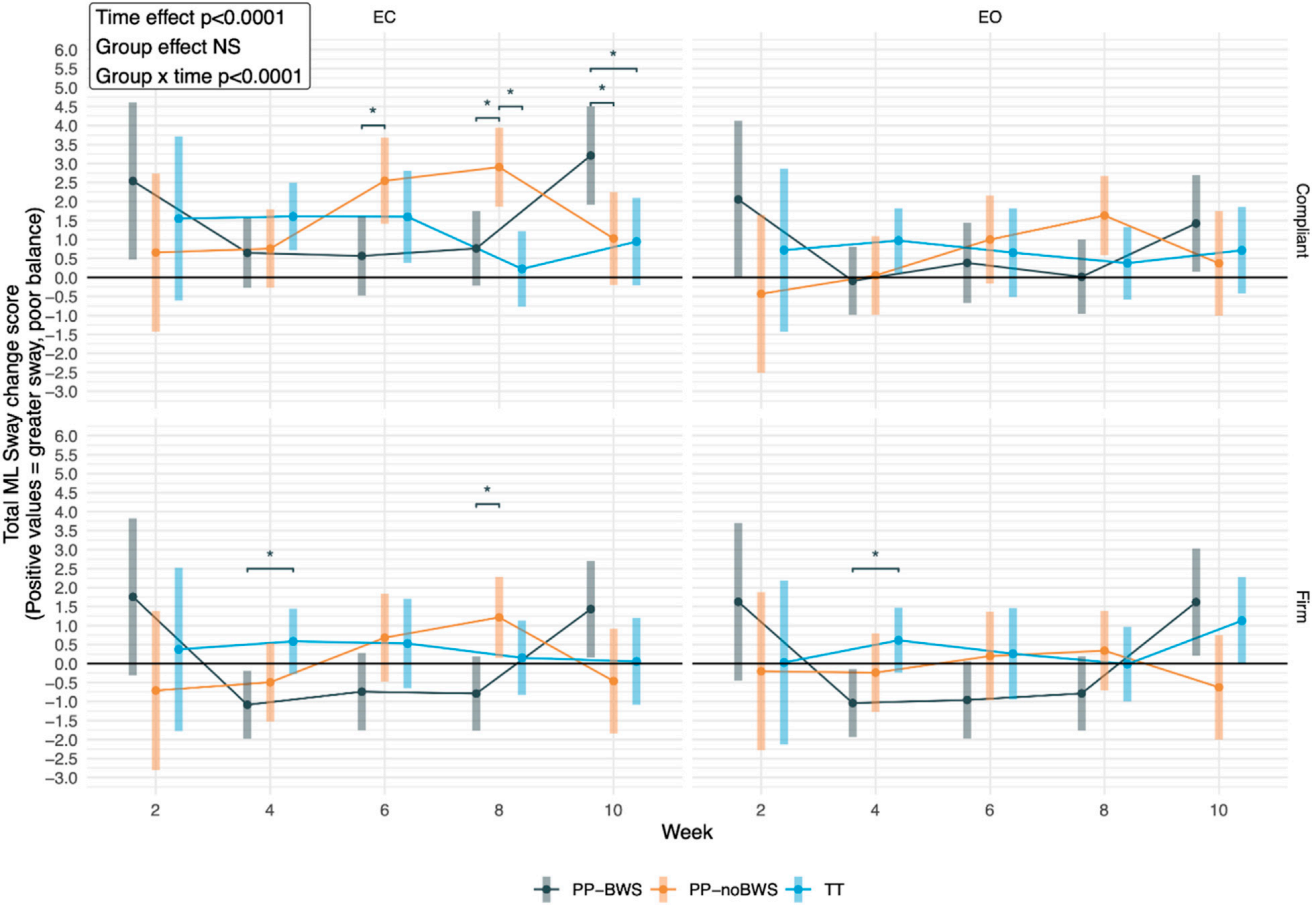
The differences in mediolateral (ML) sway among the three groups: Low body positive pressure with 20% body weight support (PP-BWS), Low body positive pressure without bodyweight support (PP-noBWS), and Traditional treadmill (TT), respectively, after 2, 4, 6, and 8 weeks of intervention, as well as at the 10-week follow-up. Significant between-group differences are marked with *.

For total ML sway, *post hoc* tests showed significant between-group differences for both eyes closed conditions at week 6 and 8. Specifically, PP-BWS swayed less than PP-noBWS under the compliant EC condition at weeks 6 and 8, and under the firm EC condition at week 8. Thus, changes in ML sway favored the PP-BWS group up to week 8, but these changes were not maintained at week 10, where instead, both the TT and PP-noBWS swayed less under the compliant EC condition. None of these between-group differences remained statistically significant in the sensitivity analyses (Figure 5). The detailed analysis and results are provided in Supplementary Material 3.

For AP sway, there were no significant effects of time, group, or a group by time interaction.

#### 3.2.5 Gait and balance app

**FIGURE 6:**
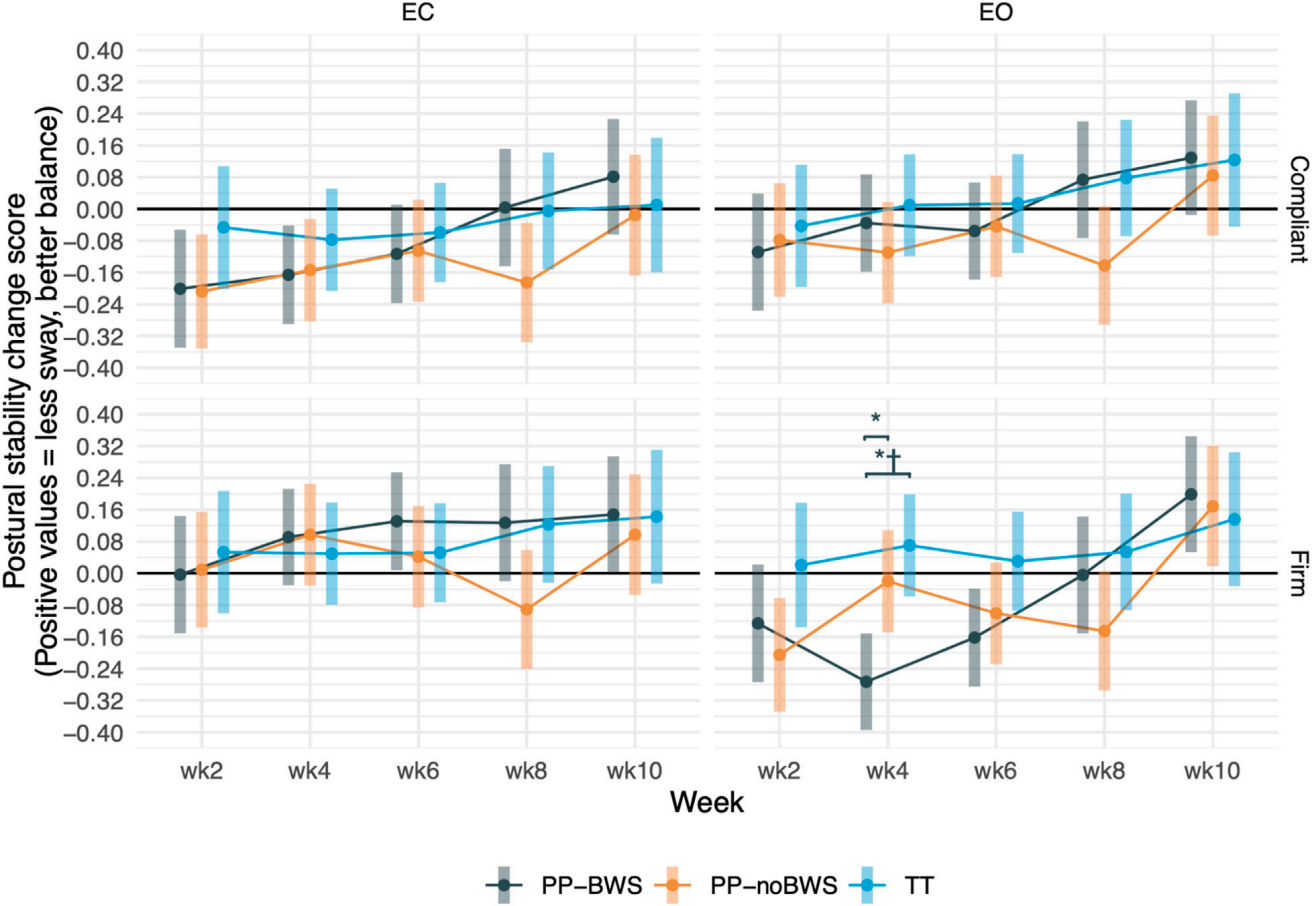
The differences in postural sturdiness among the three groups: Low body positive pressure with 20% body weight support (PP-BWS), Low body positive pressure without bodyweight support (PP-noBWS), and Traditional treadmill (TT), respectively, after 2, 4, 6, and 8 weeks of intervention, as well as at the 10-week follow-up. Significant between-group differences are marked with *, and the ones that are also significant in the sensitivity analysis are marked with †.

For the G&B app measures of postural sway (Postural Stability, AP stability, and ML stability), there was a significant effect of time for postural stability (p = 0.02) and AP stability and (p = 0.03); this is seen as gradual reduction in postural sway over time in Figure 6. There were significant effects of task by group by week for Postural stability (p = 0.02) and task by condition for ML stability (p = 0.04). However, *post hoc* between-group tests only showed a difference at week 4 for the easiest condition (Firm EO). This is shown in Figure 5 and can be seen as an outlier (where PP-BWS has increased sway at that one data point). Among G&B app outcomes, the only between-group difference that survived sensitivity analyses was PP-BWS vs. TT at week 4 on Firm EO for Postural Stability; the PP-BWS vs. PP-noBWS contrast at the same time/condition lost significance on sensitivity testing, and all other between-group contrasts remained non-significant. The detailed analysis and results are provided in Supplementary Material 4.

For G&B, walking outcomes of gait symmetry and walking speed, there were significant effects of time (p < 0.05) but not intervention group, and no interaction between group and time.

## 4 Discussion

This is the first study to explore the effectiveness of lower-body PP-BWS treadmill training, compared with traditional treadmill training and PP without body-weight support, in adults with mild balance impairment. While all three treadmill interventions resulted in within-group improvements in balance and mobility (Table 2), the primary analysis showed there were larger and more sustained gains in balance in the interventions without body weight support (traditional TT and PP-noBWS) compared with the novel PP-BWS intervention (see Figure 2). While this between-group difference was not sustained after drop outs were accounted for in the sensitivity analysis, the findings suggest that lower body PP-BWS treadmill training may be inferior to traditional TT in this adults with mild balance impairment population. Given our sample comprised Pakistani adults aged ≥50 years (an operational threshold reflecting local demographics), these findings should be extrapolated cautiously to populations defined by conventional gerontological cut-points (≥60–65 years).

The biomechanical demands of walking on a traditional treadmill are closer to over-ground walking than lower body PP training, where the waist and lower limbs are encased in an inflatable bag and body weight is supported via positive air pressure. Traditional treadmill walking requires the participant to support their full body weight, engaging the muscles and joints in a way that more closely resembles everyday activities. This functional relevance may result in more meaningful and transferable improvements in balance and mobility (Pai et al., 2014), as indicated by the higher BBS improvements in the TT group in the primary analysis. Balance measured with the FRT also improved more with traditional TT than PP-BWS, although this was not statistically significant (Figure 3). Traditional treadmill training may be more effective in enhancing balance in adults with mild balance impairment due to provision of more varied sensory feedback and the promotion of more normative muscle activation patterns. The full–weight-bearing treadmill conditions and constant belt speed will increase plantar and joint loading as well as head–trunk motion, increasing proprioceptive/vestibular challenges (Asghari et al., 2024) and necessitating more sensory reweighting (Cano Porras et al., 2020). In contrast, treadmill training with PP unloading may stabilise optic flow, reduce plantar pressure, (Mileti et al., 2020), and lower demands for rapid postural adjustments (Hodges-Long et al., 2020). This lower level of sensorimotor challenge may explain the trend toward greater improvements with traditional treadmill training over lower-body PP training.

Despite FRT scores improving up to week 8 in all groups, there was a decline across all groups at week 10 (see Figure 3), and for PP-BWS, the within-group improvements were no longer significant at week 10, suggesting improvements in balance control when reaching out of the BOS were short lasting. These results may reflect improved confidence during FRT testing during the intervention period (Schinkel-Ivy et al., 2016). Furthermore, limited FRT improvements at follow up may relate to the task-specific training effects (Weiner et al., 1992) of treadmill training, and the different domains of balance being trained versus those being tested with the FRT (Mehrholz et al., 2017). Treadmill training enhances dynamic gait stability and step coordination, but may not fully transfer to the FRT’s demand for anticipatory postural adjustments during self-initiated reaching (Wernick-Robinson et al., 1999; Moriyama et al., 2022). In contrast, the BBS assesses a broader spectrum of static and dynamic balance through 14 functional tasks (Steffen et al., 2002), and the significant between-group findings in the primary analysis suggest it may be more sensitive to the effects of treadmill training after the intervention has stopped. That said, caution is needed when interpreting the 10-week follow-up results, due to the significant dropout at week 10, and the loss of significant between-group BBS findings in the sensitivity analysis. While follow-up dropout was observed across all groups, with slightly higher attrition in the PP-noBWS and PP-BWS groups, all reported reasons were non-specific to the intervention (e.g., transport constraints, personal commitments). Nonetheless, this level of attrition may have influenced the observed effects at the 10-week mark, limiting the ability to draw firm conclusions about the sustainability of intervention effects.

The within-group tests showed that all interventions resulted in significant improvements on the TUG from baseline to post-intervention, and also at the 2-week follow-up (week 10). The earlier improvements in TUG seen in the PP-BWS group may be due to reduced joint loading and neuromuscular demand, allowing safer, more comfortable gait practice that enhances functional mobility early on (Pereira et al., 2020; Zafer et al., 2024). However, as training continued past week 4, sufficient mechanical loading likely became necessary to stimulate further neuromuscular adaptations, contributing to the plateau in TUG improvements. Thus, PP-BWS may facilitate early gains through safety and practice volume, while adequate loading supports sustained progress.

Improvements in the TUG across all groups were likely due to increased walking speed which was also seen in the G&B app data (time effect p = 0.04). While the present study did not explore underlying mechanisms, improvements in walking speed with all treadmill training interventions may relate to improvements in plantarflexion muscle strength or power (Tavakkoli Oskouei et al., 2021), improved aerobic fitness (Berryman et al., 2013), or enhanced neuromuscular activation through neural plasticity (Tavakkoli Oskouei et al., 2021). Repetitive movements are associated with cutaneous and proprioceptive impulses that may activate central pattern generators, potentiate the motor cortex, and facilitate motor learning (Asanuma and Keller, 1991). Compared with overground training, treadmill training may allow a greater number of gait cycles within each training session and enables optimization of training intensity by adjusting treadmill speed (Grecco et al., 2013). This can increase both the dose and challenge level of task-specific gait training (Mehrholz et al., 2017), providing a greater stimulus to drive changes in neural plasticity (Herrera et al., 2024), alongside adaptations in the musculoskeletal and cardiovascular systems (Berryman et al., 2013). For example, Hesse & Werner reported that stroke survivors performed up to 1,000 steps in a 20-min treadmill session compared with only 50–100 steps during a 20-min session of conventional physiotherapy (Hesse and Werner, 2003). While treadmill training offers an approach that can increase therapy dose and maximise challenge level, it is acknowledged that lasting neuroplastic changes that support walking mobility are likely to require additional training in a range of real-world environments that introduce novel and functionally-meaningful tasks (Thomas et al., 2012; Shi et al., 2025).

Even though the effects of lower body PP-BWS were not maintained at follow-up, this approach may still be useful for people who have pain or fear during traditional TT, such as those with knee osteoarthritis (Chen H.-X. et al., 2021) or a history of falls (Lazaro, 2020). Other populations that have benefited from lower body PP-BWS include those with requiring additional BWS, such as people with cerebral palsy (Alwhaibi et al., 2022) and stroke (Almutairi S., 2023; Almutairi S. M., 2023), although there is limited evidence of any superiority over other gait training methods (Almutairi S. M., 2023). To enhance the carry-over effect into functional mobility, lower body PP-BWS could be combined with overground training. This approach that has shown promise in stroke and spinal cord injury populations (Yang et al., 2022; Almutairi S., 2023), where lower body PP-BWS training combined with overground training was superior to PP-BWS alone, for improving gait symmetry, walking speed, and community ambulation.

### 4.1 Strengths and limitations

This study incorporated several methodological features that support the credibility of its findings. The control group was an attention and dose-matched traditional treadmill intervention to ensure the added benefit of “positive pressure training” could be investigated. Another strength is that we assessed both standardised clinical outcomes (e.g., BBS, TUG, FRT) and biomechanical measures (e.g., postural sway, gait parameters), providing a broader understanding of intervention effects. The sample size was determined using a power calculation for the primary outcome (BBS), which strengthened confidence in detecting meaningful changes for this measure. At the same time, we acknowledge that the study was not powered for secondary outcomes such as postural sway or gait, so these results should be interpreted with caution due to the potential for Type II error. Blinding of assessors and data analysts was implemented to help reduce assessment bias and improve internal validity. In addition, the intervention protocols were clearly defined, and validated outcome measures were employed, which enhances the reproducibility and transparency of the research.

The study included a relatively homogenous population of people with mild balance impairment, and therefore, the findings should not be generalised to populations with more severe gait and balance impairment. Future investigations may benefit from directly comparing these training modalities across different functional baselines and demographic groups. A key limitation of the study was the participant attrition by the 10-week follow-up. Although reasons were unrelated to the intervention itself. Future studies may benefit from strategies to improve follow-up compliance, such as more flexible assessment scheduling or transport assistance.

The study design included three different types of treadmill training to enable the investigation of the effects of the positive pressure component. This design did not include a no-exercise control group due to ethical concerns about withholding exercise from adults with mild balance impairment at mild fall risk. This may raise concerns that the within-group effects on balance and mobility could be attributed to a familiarization with the outcome measurements. However, this is thought to be unlikely, as previous older adult treadmill studies have failed to show significant within-group effects in no-exercise control groups (Pirouzi et al., 2014; Pereira et al., 2020). Furthermore, the within-group changes in BBS and TUG in the present study exceeded measurement error (Muir-Hunter et al., 2015; Smith et al., 2016), suggesting changes were more likely attributable to the effects of treadmill training rather than mere familiarization. However, to better distinguish treadmill training effects from natural progression or test familiarity, future research could include a no-exercise control group and address ethical concerns by offering control participants access to the intervention following the study conclusion. The sample had a predominance of females, which may limit generalizability to male populations, although sex was included as a covariate in the analyses.

## 5 Conclusion

Treadmill training interventions, regardless of whether body weight support was used, improved balance and mobility over the 10-week period in older adults with mild balance impairment with mild balance impairment. The primary between-group analysis highlighted the potential effectiveness of treadmill interventions without body weight support—traditional treadmill training and lower body PP treadmill training without BWS—over lower body PP-BWS treadmill training, for improving balance on the BBS. This may suggest that the altered gait mechanics and reduced sensory feedback with PP training may limit improvements in balance, however, these between-group differences were not upheld after dropouts were accounted for. Although lower-body PP training with 20% BWS showed initial benefits for walking mobility on the TUG, these gains were not apparent after week 4. This aligned with smartphone accelerometry outcomes, which showed no between-group differences for comfortable walking speed and gait symmetry. While significant between-group differences were not established over the 10 weeks in this study, the smaller sample size and large dropouts at follow up may have limited the detection of between-group differences. The trends supported traditional treadmill approaches over positive pressure training, but given all interventions resulted in within-group improvements, clinicians should balance both the potential unwanted effects of lower-body PP training, such as disrupted natural movement patterns, as well as its possible advantages, including pain relief and reduced fear of falling, when designing rehabilitation programs.

## Data Availability

Reasonable request for data can be requested from the corresponding author but we will need to seek ethics committee approval prior to sharing any data. Requests to access the datasets should be directed to dr.hinashafi.89@gmail.com.
