# Supplementary material for: Comparative effectiveness of lower body positive pressure and traditional treadmill training on adults with mild balance impairment
Source: Front Aging. 2025 Oct 22;6:1645026. doi: 10.3389/fragi.2025.1645026 (PMC12586058; doi:10.3389/fragi.2025.1645026)
Supplement: Supplementary file 1 [file DataSheet2.pdf]

AGT RCT: Clinical

Usman Rashid

13/09/2025

Contents

1 Baseline Score & Characteristics **2**

2 Missingness **3**

3 Statistical Analysis **4**

4 BBS **4**
4.1 Diagnostics 4
4.2 Anova 4
4.3 Between Group Difference in Change Score 5
4.4 Within Group Change Score 5
4.5 Change Score Plot 6

5 FRT **7**
5.1 Diagnostics 7
5.2 Anova 7
5.3 Between Group Difference in Change Score 8
5.4 Within Group Change Score 8
5.5 Change Score Plot 9

6 TUG **10**
6.1 Diagnostics 10
6.2 Anova 10
6.3 Between Group Difference in Change Score 11
6.4 Within Group Change Score 11
6.5 Change Score Plot 12

7 Sensitivity Analysis by Multiple-imputations **13**
7.1 BBS 13
7.1.1 Between Group Difference in Change Score 13
7.2 Within Group Change Score 13
7.3 FRT 14
7.3.1 Between Group Difference in Change Score 14
7.4 Within Group Change Score 14
7.5 TUG 15
7.5.1 Between Group Difference in Change Score 15
7.6 Within Group Change Score 15

1 Baseline Score & Characteristics

Variable	PP-BWS	PP-noBWS	TT
BBS_Baseline	50.8 ± 2.397	52 ± 1.556	51.25 ± 1.333
FRT_Baseline	8.77 ± 2.712	9.434 ± 2.408	10.645 ± 2.082
TUG_Baseline	12.161 ± 2.839	12.428 ± 2.29	13.341 ± 3.274
age	57.95 ± 6.32	54.65 ± 4.295	55.05 ± 5.453
bmi	29.343 ± 4.477	28.635 ± 6.872	28.885 ± 4.839
height_cm	158.9 ± 10.249	165 ± 8.639	164.45 ± 9.151
weight_kg	73.828 ± 13.804	77.38 ± 16.778	77.755 ± 11.956

2 Missingness

Timepoint	Group	Allocated	n	Missingness
wk2	PP-BWS	23	20	13.0
wk2	PP-noBWS	25	19	24.0
wk2	TT	24	18	25.0
wk4	PP-BWS	23	19	17.4
wk4	PP-noBWS	25	18	28.0
wk4	TT	24	20	16.7
wk6	PP-BWS	23	19	17.4
wk6	PP-noBWS	25	17	32.0
wk6	TT	24	20	16.7
wk8	PP-BWS	23	18	21.7
wk8	PP-noBWS	25	17	32.0
wk8	TT	24	20	16.7
wk10	PP-BWS	23	10	56.5
wk10	PP-noBWS	25	9	64.0
wk10	TT	24	10	58.3

3 Statistical Analysis

Statistical models are fitted to change scores (Post- - Pre-intervention values). Thus, a positive score implies that the post-intervention value is larger than the pre-intervention value. Benjamini-Hochberg adjustment is applied for between group differences.

4 BBS

```
mdl.bbs <- lme4::lmer((Outcome - OutcomePre) ~ OutcomePre + Group*Timepoint +
                        height_cm + bmi + age + Gender +
                        (1|Id),
                      subset(clinical.csv.long, Scale == "BBS"))
```

4.1 Diagnostics

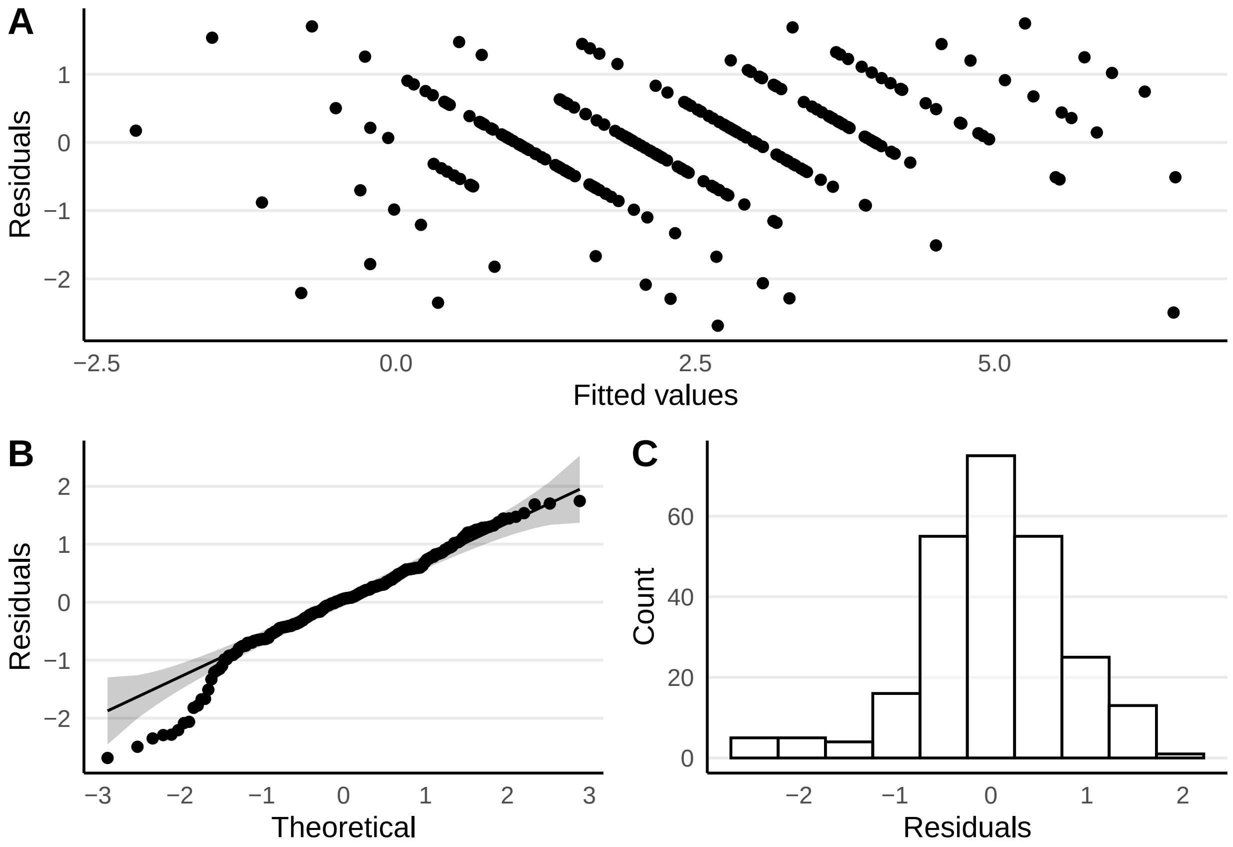

4.2 Anova

```
Analysis of Deviance Table (Type III Wald chisquare tests)

Response: (Outcome - OutcomePre)
                    Chisq Df Pr(>Chisq)
```

```
(Intercept)     66.9339  1  2.808e-16 ***
OutcomePre      86.5156  1  < 2.2e-16 ***
Group            2.6765  2   0.262300
Timepoint       24.9254  4  5.208e-05 ***
height_cm        0.2994  1   0.584263
bmi              0.7147  1   0.397887
age              1.9288  1   0.164885
Gender           5.9138  1   0.015023 *
Group:Timepoint 23.1525  8   0.003174 **
---
Signif. codes:  0 '***' 0.001 '**' 0.01 '*' 0.05 '.' 0.1 ' ' 1
```

4.3 Between Group Difference in Change Score

Contrast	Timepoint	Difference±SE [95% CI], SMD	t[df], p-value
(PP-BWS) - (PP-noBWS)	wk2	0.6±0.4 [-0.4, 1.6], 0.14	t[121.8]=1.541, 0.31
(PP-BWS) - TT	wk2	0.5±0.4 [-0.5, 1.5], 0.11	t[131.6]=1.269, 0.31
(PP-noBWS) - TT	wk2	-0.1±0.4 [-1.1, 0.8], -0.03	t[139.1]=-0.303, 0.763
(PP-BWS) - (PP-noBWS)	wk4	-0.3±0.4 [-1.3, 0.7], -0.07	t[125.4]=-0.752, 0.701
(PP-BWS) - TT	wk4	-0.3±0.4 [-1.2, 0.7], -0.06	t[127.5]=-0.729, 0.701
(PP-noBWS) - TT	wk4	0±0.4 [-0.9, 1], 0.01	t[135.1]=0.059, 0.953
(PP-BWS) - (PP-noBWS)	wk6	0.3±0.4 [-0.7, 1.3], 0.06	t[128.1]=0.673, 0.731
(PP-BWS) - TT	wk6	-0.1±0.4 [-1.1, 0.8], -0.03	t[127.5]=-0.344, 0.731
(PP-noBWS) - TT	wk6	-0.4±0.4 [-1.4, 0.5], -0.09	t[138.3]=-1.05, 0.731
(PP-BWS) - (PP-noBWS)	wk8	-0.3±0.4 [-1.3, 0.7], -0.06	t[129.7]=-0.737, 0.463
(PP-BWS) - TT	wk8	-0.8±0.4 [-1.8, 0.1], -0.19	t[129.5]=-2.152, 0.1
(PP-noBWS) - TT	wk8	-0.5±0.4 [-1.5, 0.4], -0.12	t[138.1]=-1.378, 0.256
(PP-BWS) - (PP-noBWS)	wk10	-1.2±0.5 [-2.5, 0], -0.17	t[194.5]=-2.435, 0.027
(PP-BWS) - TT	wk10	-1.2±0.5 [-2.3, 0], -0.17	t[197.9]=-2.382, 0.027
(PP-noBWS) - TT	wk10	0.1±0.5 [-1.1, 1.3], 0.01	t[207.5]=0.145, 0.884

4.4 Within Group Change Score

Group	Timepoint	Estimate±SE [95% CI], SMD	t[df], p-value
PP-BWS	wk2	2.1±0.3 [1.5, 2.7], 0.68	t[114.1]=7.275, <0.001
PP-noBWS	wk2	1.5±0.3 [0.9, 2], 0.46	t[131.9]=5.328, <0.001
TT	wk2	1.6±0.3 [1, 2.2], 0.48	t[135]=5.593, <0.001
PP-BWS	wk4	1.8±0.3 [1.2, 2.4], 0.56	t[118.9]=6.14, <0.001
PP-noBWS	wk4	2.1±0.3 [1.6, 2.7], 0.64	t[135.7]=7.469, <0.001
TT	wk4	2.1±0.3 [1.5, 2.6], 0.68	t[124.3]=7.573, <0.001
PP-BWS	wk6	2.8±0.3 [2.2, 3.4], 0.87	t[118.9]=9.541, <0.001
PP-noBWS	wk6	2.5±0.3 [2, 3.1], 0.73	t[142.5]=8.754, <0.001
TT	wk6	2.9±0.3 [2.4, 3.5], 0.96	t[124.3]=10.653, <0.001
PP-BWS	wk8	3±0.3 [2.5, 3.6], 0.92	t[123.2]=10.235, <0.001

Group	Timepoint	Estimate±SE [95% CI], SMD	t[df], p-value
PP-noBWS	wk8	3.4±0.3 [2.8, 3.9], 0.97	t[142]=11.615, <0.001
TT	wk8	3.9±0.3 [3.3, 4.4], 1.26	t[124.3]=14.096, <0.001
PP-BWS	wk10	2.2±0.4 [1.5, 2.9], 0.45	t[184.1]=6.097, <0.001
PP-noBWS	wk10	3.4±0.4 [2.7, 4.2], 0.65	t[209.5]=9.385, <0.001
TT	wk10	3.4±0.4 [2.7, 4.1], 0.68	t[198]=9.572, <0.001

4.5 Change Score Plot

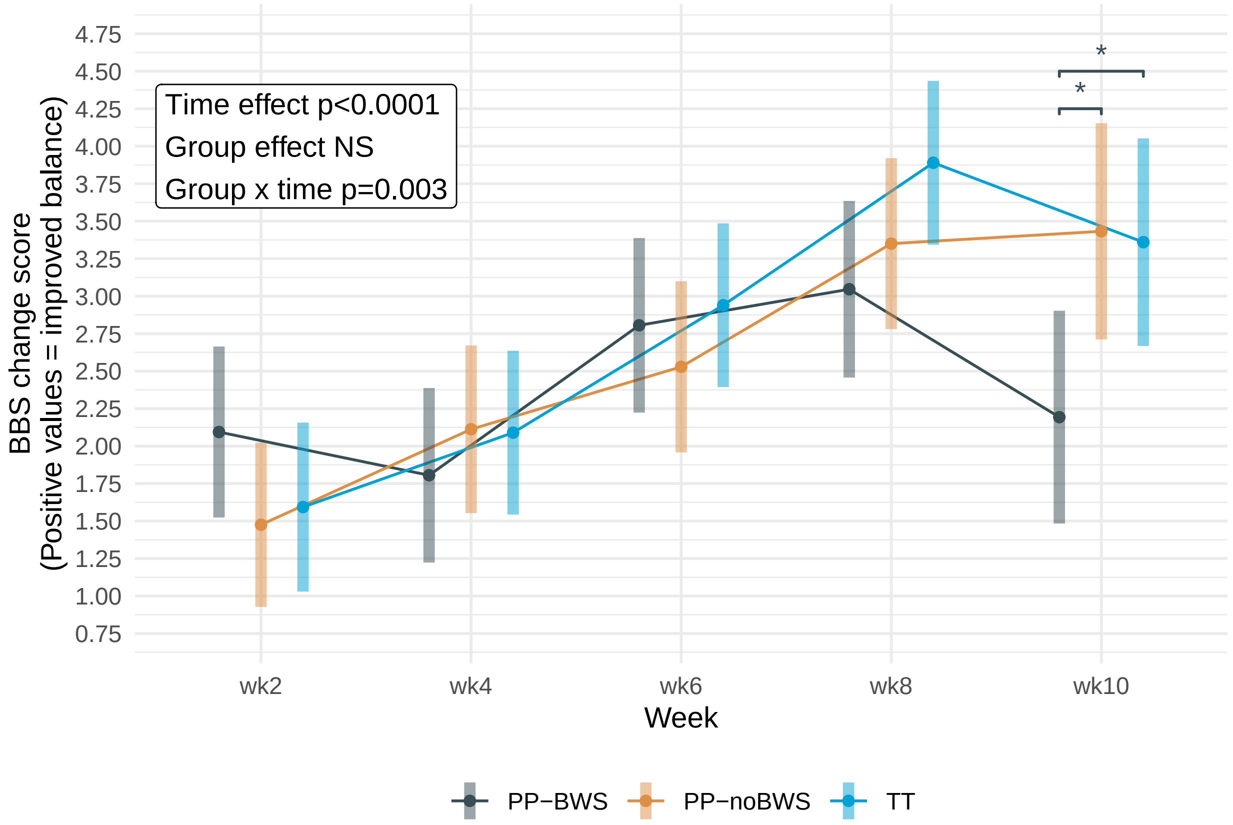

5 FRT

```
mdl.frt <- lme4::lmer((Outcome - OutcomePre) ~ OutcomePre + Group*Timepoint +
                        height_cm + bmi + age + Gender +
                        (1|Id),
                      subset(clinical.csv.long, Scale == "FRT"))
```

5.1 Diagnostics

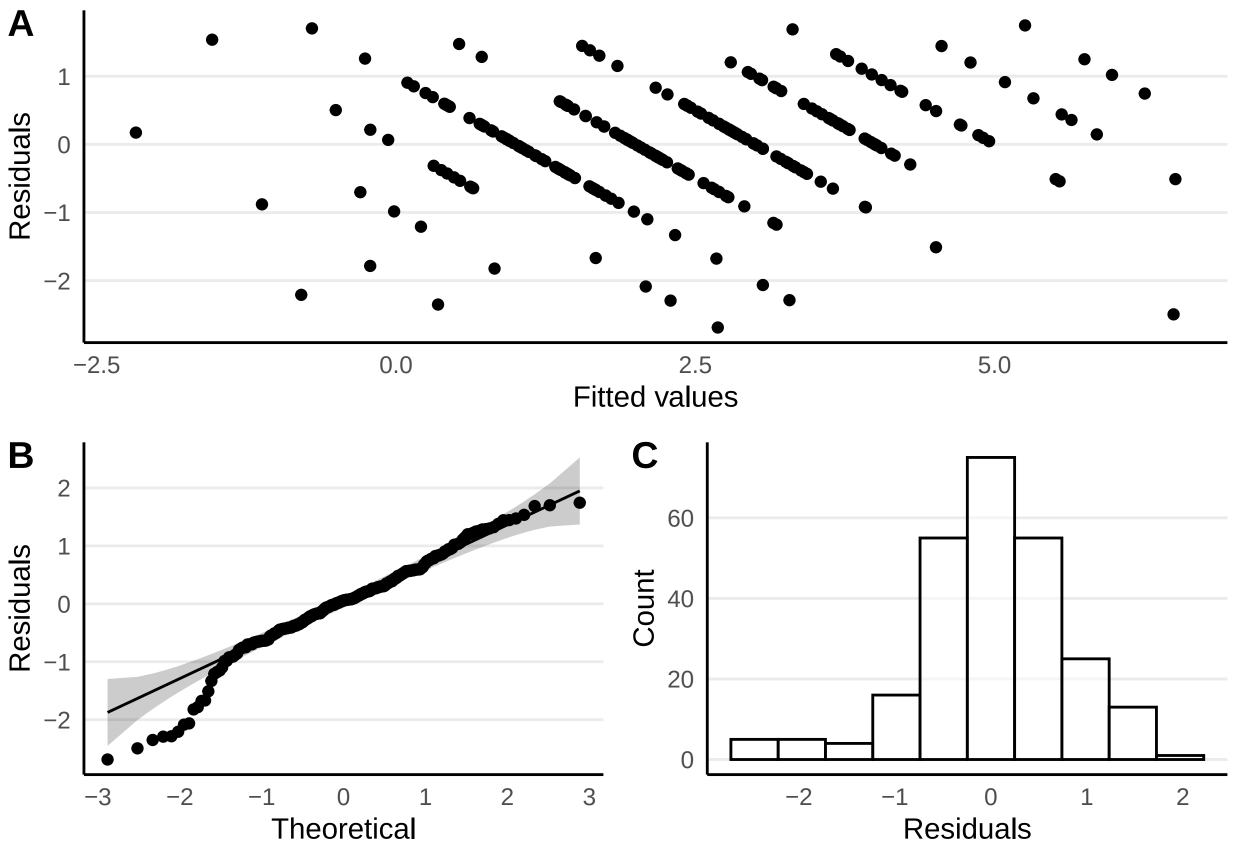

5.2 Anova

```
Analysis of Deviance Table (Type III Wald chisquare tests)

Response: (Outcome - OutcomePre)
                  Chisq Df Pr(>Chisq)
(Intercept)      0.3647  1    0.54588
OutcomePre      51.1352  1  8.622e-13 ***
Group            0.4632  2    0.79328
Timepoint        9.9778  4    0.04080 *
height_cm        4.2335  1    0.03963 *
bmi              0.0099  1    0.92056
age              4.6663  1    0.03076 *
```

```
Gender           0.2596  1    0.61042
Group:Timepoint  7.1380  8    0.52182
---
Signif. codes:  0 '***' 0.001 '**' 0.01 '*' 0.05 '.' 0.1 ' ' 1
```

5.3 Between Group Difference in Change Score

Contrast	Timepoint	Difference±SE [95% CI], SMD	t[df], p-value
(PP-BWS) - (PP-noBWS)	wk2	0.2±0.6 [-1.2, 1.6], 0.03	t[100.3]=0.303, 0.762
(PP-BWS) - TT	wk2	-0.2±0.6 [-1.7, 1.2], -0.04	t[104.6]=-0.37, 0.762
(PP-noBWS) - TT	wk2	-0.4±0.6 [-1.8, 1], -0.06	t[111.3]=-0.68, 0.762
(PP-BWS) - (PP-noBWS)	wk4	-0.5±0.6 [-1.9, 0.9], -0.08	t[99.9]=-0.815, 0.626
(PP-BWS) - TT	wk4	-0.5±0.6 [-2, 0.9], -0.09	t[99.3]=-0.914, 0.626
(PP-noBWS) - TT	wk4	-0.1±0.6 [-1.5, 1.3], -0.01	t[106.5]=-0.113, 0.91
(PP-BWS) - (PP-noBWS)	wk6	0.2±0.6 [-1.2, 1.7], 0.04	t[106.7]=0.385, 0.701
(PP-BWS) - TT	wk6	-0.5±0.6 [-1.9, 1], -0.08	t[101]=-0.823, 0.619
(PP-noBWS) - TT	wk6	-0.7±0.6 [-2.1, 0.7], -0.11	t[112.5]=-1.216, 0.619
(PP-BWS) - (PP-noBWS)	wk8	-0.4±0.6 [-1.9, 1], -0.07	t[106.7]=-0.709, 0.48
(PP-BWS) - TT	wk8	-1±0.6 [-2.4, 0.5], -0.17	t[101]=-1.663, 0.298
(PP-noBWS) - TT	wk8	-0.6±0.6 [-2, 0.9], -0.09	t[112.5]=-0.958, 0.48
(PP-BWS) - (PP-noBWS)	wk10	-0.7±0.7 [-2.4, 1], -0.08	t[166.7]=-1.014, 0.312
(PP-BWS) - TT	wk10	-1.6±0.7 [-3.3, 0.2], -0.16	t[170.2]=-2.147, 0.1
(PP-noBWS) - TT	wk10	-0.8±0.7 [-2.6, 0.9], -0.08	t[184]=-1.15, 0.312

5.4 Within Group Change Score

Group	Timepoint	Estimate±SE [95% CI], SMD	t[df], p-value
PP-BWS	wk2	0.8±0.4 [0, 1.6], 0.2	t[93.1]=1.888, 0.062
PP-noBWS	wk2	0.6±0.4 [-0.2, 1.4], 0.15	t[108.8]=1.568, 0.12
TT	wk2	1±0.4 [0.1, 1.9], 0.22	t[107.7]=2.305, 0.023
PP-BWS	wk4	0.6±0.4 [-0.2, 1.5], 0.16	t[93.1]=1.499, 0.137
PP-noBWS	wk4	1.1±0.4 [0.3, 1.9], 0.27	t[108.8]=2.779, 0.006
TT	wk4	1.2±0.4 [0.3, 2], 0.27	t[98.7]=2.725, 0.008
PP-BWS	wk6	1.4±0.4 [0.5, 2.2], 0.32	t[97.3]=3.191, 0.002
PP-noBWS	wk6	1.1±0.4 [0.3, 2], 0.25	t[120.2]=2.764, 0.007
TT	wk6	1.9±0.4 [1, 2.7], 0.43	t[98.7]=4.319, <0.001
PP-BWS	wk8	1.5±0.4 [0.6, 2.3], 0.34	t[97.3]=3.395, <0.001
PP-noBWS	wk8	1.9±0.4 [1.1, 2.7], 0.42	t[120]=4.552, <0.001
TT	wk8	2.4±0.4 [1.6, 3.3], 0.57	t[98.7]=5.679, <0.001
PP-BWS	wk10	0.4±0.5 [-0.6, 1.4], 0.06	t[153.4]=0.748, 0.456
PP-noBWS	wk10	1.1±0.5 [0.1, 2.1], 0.16	t[185.7]=2.173, 0.031
TT	wk10	1.9±0.5 [0.9, 3], 0.27	t[175.3]=3.577, <0.001

5.5 Change Score Plot

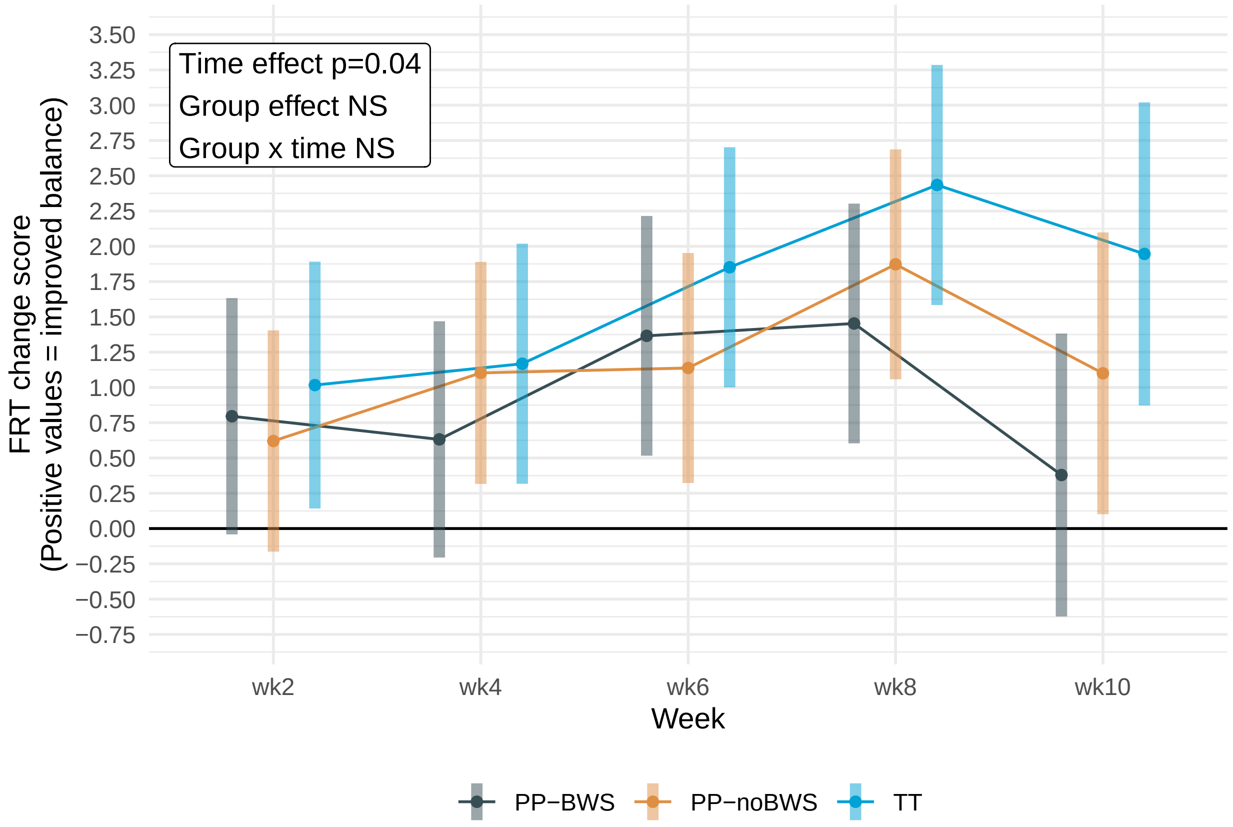

6 TUG

```
mdl.tug <- lme4::lmer((Outcome - OutcomePre) ~ OutcomePre + Group*Timepoint +
                          height_cm + bmi + age + Gender +
                          (1|Id),
                        subset(clinical.csv.long, Scale == "TUG"))
```

6.1 Diagnostics

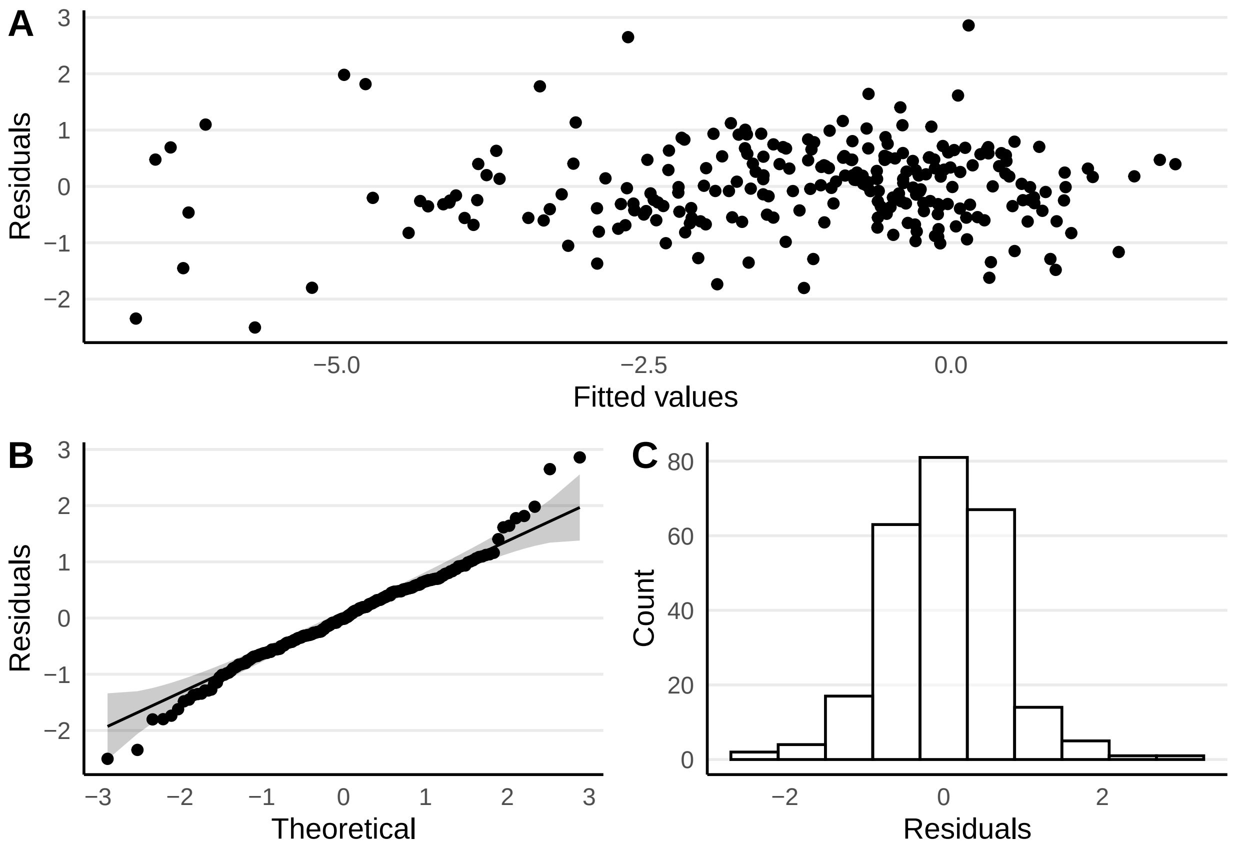

6.2 Anova

```
Analysis of Deviance Table (Type III Wald chisquare tests)

Response: (Outcome - OutcomePre)
                  Chisq Df Pr(>Chisq)
(Intercept)      1.6367  1   0.200782
OutcomePre      98.1512  1  < 2.2e-16 ***
Group           11.6732  2   0.002919 **
Timepoint        3.7237  4   0.444686
height_cm        0.0014  1   0.969932
bmi              9.0254  1   0.002663 **
age              4.8573  1   0.027529 *
```

```
Gender           2.7102  1   0.099707 .
Group:Timepoint 17.1787  8   0.028301 *
---
Signif. codes:  0 '***' 0.001 '**' 0.01 '*' 0.05 '.' 0.1 ' ' 1
```

6.3 Between Group Difference in Change Score

Contrast	Timepoint	Difference±SE [95% CI], SMD	t[df], p-value
(PP-BWS) - (PP-noBWS)	wk2	-1.4±0.4 [-2.3, -0.4], -0.32	t[112.5]=-3.383, 0.003
(PP-BWS) - TT	wk2	-0.6±0.4 [-1.5, 0.4], -0.13	t[116.8]=-1.403, 0.163
(PP-noBWS) - TT	wk2	0.8±0.4 [-0.1, 1.7], 0.18	t[128.3]=2.054, 0.063
(PP-BWS) - (PP-noBWS)	wk4	-1.2±0.4 [-2.2, -0.2], -0.28	t[111.8]=-3.01, 0.01
(PP-BWS) - TT	wk4	-0.9±0.4 [-1.9, 0], -0.22	t[111.8]=-2.319, 0.033
(PP-noBWS) - TT	wk4	0.3±0.4 [-0.6, 1.2], 0.07	t[122.8]=0.758, 0.45
(PP-BWS) - (PP-noBWS)	wk6	-0.6±0.4 [-1.6, 0.4], -0.13	t[119.1]=-1.441, 0.228
(PP-BWS) - TT	wk6	-0.6±0.4 [-1.6, 0.4], -0.14	t[113.5]=-1.476, 0.228
(PP-noBWS) - TT	wk6	0±0.4 [-0.9, 1], 0	t[128.6]=0.014, 0.989
(PP-BWS) - (PP-noBWS)	wk8	-0.3±0.4 [-1.3, 0.8], -0.05	t[121.8]=-0.605, 0.546
(PP-BWS) - TT	wk8	0.3±0.4 [-0.7, 1.3], 0.07	t[116.3]=0.721, 0.546
(PP-noBWS) - TT	wk8	0.5±0.4 [-0.4, 1.5], 0.12	t[128.5]=1.387, 0.504
(PP-BWS) - (PP-noBWS)	wk10	-0.6±0.5 [-1.9, 0.6], -0.09	t[190.6]=-1.216, 0.611
(PP-BWS) - TT	wk10	-0.4±0.5 [-1.6, 0.8], -0.06	t[181.6]=-0.831, 0.611
(PP-noBWS) - TT	wk10	0.2±0.5 [-1, 1.4], 0.03	t[205.5]=0.433, 0.666

6.4 Within Group Change Score

Group	Timepoint	Estimate±SE [95% CI], SMD	t[df], p-value
PP-BWS	wk2	-1.5±0.3 [-2.1, -0.9], -0.5	t[101.2]=-5.04, <0.001
PP-noBWS	wk2	-0.1±0.3 [-0.7, 0.4], -0.05	t[122.8]=-0.507, 0.613
TT	wk2	-0.9±0.3 [-1.5, -0.4], -0.3	t[123.4]=-3.278, 0.001
PP-BWS	wk4	-1.7±0.3 [-2.3, -1.1], -0.56	t[101.2]=-5.647, <0.001
PP-noBWS	wk4	-0.5±0.3 [-1, 0.1], -0.15	t[122.3]=-1.713, 0.089
TT	wk4	-0.8±0.3 [-1.3, -0.2], -0.26	t[113.7]=-2.749, 0.007
PP-BWS	wk6	-1.8±0.3 [-2.4, -1.2], -0.57	t[105.7]=-5.902, <0.001
PP-noBWS	wk6	-1.2±0.3 [-1.8, -0.6], -0.36	t[134.1]=-4.159, <0.001
TT	wk6	-1.2±0.3 [-1.8, -0.6], -0.4	t[113.7]=-4.311, <0.001
PP-BWS	wk8	-1.9±0.3 [-2.5, -1.3], -0.59	t[110.1]=-6.221, <0.001
PP-noBWS	wk8	-1.7±0.3 [-2.2, -1.1], -0.5	t[133.7]=-5.79, <0.001
TT	wk8	-2.2±0.3 [-2.8, -1.7], -0.74	t[113.7]=-7.915, <0.001
PP-BWS	wk10	-2.1±0.4 [-2.8, -1.4], -0.45	t[164.8]=-5.733, <0.001
PP-noBWS	wk10	-1.4±0.4 [-2.2, -0.7], -0.26	t[212.3]=-3.849, <0.001
TT	wk10	-1.7±0.3 [-2.4, -1], -0.35	t[186.7]=-4.784, <0.001

6.5 Change Score Plot

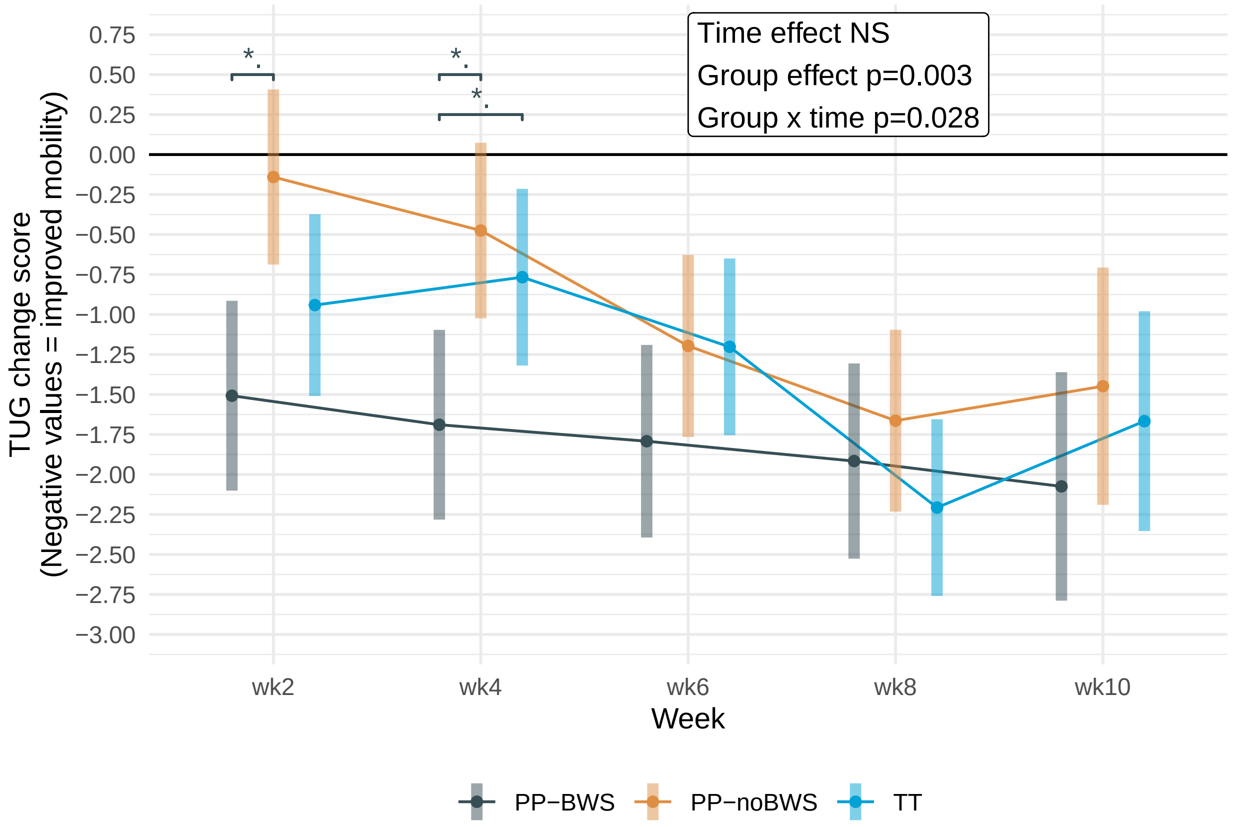

7 Sensitivity Analysis by Multiple-imputations

7.1 BBS

7.1.1 Between Group Difference in Change Score

Contrast	Timepoint	Difference±SE [95% CI], SMD	t[df], p-value
(PP-BWS) - (PP-noBWS)	wk2	0.6±0.4 [-0.4, 1.6], 0.12	t[172.4]=1.512, 0.373
(PP-BWS) - TT	wk2	0.5±0.4 [-0.5, 1.4], 0.09	t[163.6]=1.157, 0.373
(PP-noBWS) - TT	wk2	-0.1±0.4 [-1.1, 0.8], -0.03	t[165.2]=-0.333, 0.739
(PP-BWS) - (PP-noBWS)	wk4	-0.3±0.4 [-1.3, 0.7], -0.05	t[159.6]=-0.659, 0.766
(PP-BWS) - TT	wk4	-0.3±0.4 [-1.2, 0.6], -0.06	t[182.1]=-0.81, 0.766
(PP-noBWS) - TT	wk4	0±0.4 [-1, 0.9], -0.01	t[178.7]=-0.116, 0.908
(PP-BWS) - (PP-noBWS)	wk6	0.2±0.4 [-0.8, 1.2], 0.04	t[150.2]=0.509, 0.655
(PP-BWS) - TT	wk6	-0.2±0.4 [-1.1, 0.8], -0.03	t[181.3]=-0.448, 0.655
(PP-noBWS) - TT	wk6	-0.4±0.4 [-1.3, 0.6], -0.07	t[171.8]=-0.976, 0.655
(PP-BWS) - (PP-noBWS)	wk8	-0.2±0.4 [-1.2, 0.7], -0.05	t[165.8]=-0.611, 0.542
(PP-BWS) - TT	wk8	-0.8±0.4 [-1.8, 0.1], -0.16	t[178.4]=-2.128, 0.104
(PP-noBWS) - TT	wk8	-0.6±0.4 [-1.5, 0.3], -0.11	t[185.6]=-1.524, 0.194
(PP-BWS) - (PP-noBWS)	wk10	-0.4±0.5 [-1.7, 0.9], -0.09	t[75.1]=-0.781, 0.437
(PP-BWS) - TT	wk10	-0.8±0.5 [-2, 0.5], -0.18	t[73.2]=-1.54, 0.384
(PP-noBWS) - TT	wk10	-0.4±0.4 [-1.5, 0.7], -0.08	t[117.5]=-0.884, 0.437

7.2 Within Group Change Score

Group	Timepoint	Estimate±SE [95% CI], SMD	t[df], p-value
PP-BWS	wk2	2±0.3 [1.5, 2.6], 0.56	t[169]=7.24, <0.001
PP-noBWS	wk2	1.4±0.3 [0.9, 2], 0.39	t[180.9]=5.262, <0.001
TT	wk2	1.6±0.3 [1, 2.2], 0.44	t[143.9]=5.324, <0.001
PP-BWS	wk4	1.7±0.3 [1.2, 2.3], 0.48	t[158.1]=5.984, <0.001
PP-noBWS	wk4	2±0.3 [1.4, 2.6], 0.55	t[164.5]=7.064, <0.001
TT	wk4	2±0.3 [1.5, 2.6], 0.55	t[183.6]=7.517, <0.001
PP-BWS	wk6	2.7±0.3 [2.1, 3.3], 0.75	t[156.1]=9.353, <0.001
PP-noBWS	wk6	2.5±0.3 [1.9, 3.1], 0.71	t[145]=8.535, <0.001
TT	wk6	2.9±0.3 [2.4, 3.4], 0.78	t[183.6]=10.635, <0.001
PP-BWS	wk8	3±0.3 [2.4, 3.6], 0.83	t[151.7]=10.258, <0.001
PP-noBWS	wk8	3.3±0.3 [2.7, 3.8], 0.89	t[169.1]=11.609, <0.001
TT	wk8	3.8±0.3 [3.3, 4.4], 1.04	t[183.6]=14.121, <0.001
PP-BWS	wk10	2.6±0.4 [1.9, 3.4], 0.85	t[66.8]=6.947, <0.001
PP-noBWS	wk10	3±0.3 [2.4, 3.7], 0.96	t[83.2]=8.778, <0.001
TT	wk10	3.4±0.3 [2.8, 4.1], 1.05	t[97.4]=10.356, <0.001

7.3 FRT

7.3.1 Between Group Difference in Change Score

Contrast	Timepoint	Difference±SE [95% CI], SMD	t[df], p-value
(PP-BWS) - (PP-noBWS)	wk2	0.2±0.6 [-1.1, 1.6], 0.04	t[125.3]=0.43, 0.668
(PP-BWS) - TT	wk2	-0.5±0.6 [-1.8, 0.9], -0.07	t[128.1]=-0.845, 0.599
(PP-noBWS) - TT	wk2	-0.7±0.6 [-2.1, 0.6], -0.11	t[128.6]=-1.284, 0.599
(PP-BWS) - (PP-noBWS)	wk4	-0.4±0.6 [-1.8, 0.9], -0.07	t[127.7]=-0.778, 0.643
(PP-BWS) - TT	wk4	-0.7±0.6 [-2, 0.7], -0.11	t[131.4]=-1.245, 0.643
(PP-noBWS) - TT	wk4	-0.3±0.5 [-1.6, 1.1], -0.04	t[135.3]=-0.464, 0.643
(PP-BWS) - (PP-noBWS)	wk6	0.3±0.6 [-1.1, 1.7], 0.05	t[120.1]=0.559, 0.577
(PP-BWS) - TT	wk6	-0.6±0.6 [-2, 0.8], -0.1	t[128]=-1.08, 0.423
(PP-noBWS) - TT	wk6	-0.9±0.6 [-2.3, 0.4], -0.15	t[128.9]=-1.661, 0.298
(PP-BWS) - (PP-noBWS)	wk8	-0.3±0.6 [-1.7, 1.1], -0.04	t[124.7]=-0.488, 0.626
(PP-BWS) - TT	wk8	-1.1±0.6 [-2.5, 0.3], -0.17	t[127.6]=-1.973, 0.152
(PP-noBWS) - TT	wk8	-0.8±0.6 [-2.2, 0.5], -0.13	t[132.2]=-1.503, 0.203
(PP-BWS) - (PP-noBWS)	wk10	-0.2±0.7 [-1.9, 1.5], -0.03	t[70.9]=-0.29, 0.773
(PP-BWS) - TT	wk10	-1.2±0.7 [-2.8, 0.4], -0.21	t[81.1]=-1.896, 0.185
(PP-noBWS) - TT	wk10	-1±0.7 [-2.7, 0.6], -0.18	t[69.8]=-1.524, 0.198

7.4 Within Group Change Score

Group	Timepoint	Estimate±SE [95% CI], SMD	t[df], p-value
PP-BWS	wk2	0.7±0.4 [-0.1, 1.5], 0.15	t[121.8]=1.668, 0.098
PP-noBWS	wk2	0.4±0.4 [-0.3, 1.2], 0.1	t[130.6]=1.106, 0.271
TT	wk2	1.1±0.4 [0.3, 2], 0.26	t[120.8]=2.831, 0.005
PP-BWS	wk4	0.5±0.4 [-0.3, 1.3], 0.11	t[121.8]=1.264, 0.209
PP-noBWS	wk4	0.9±0.4 [0.2, 1.7], 0.21	t[134.1]=2.448, 0.016
TT	wk4	1.2±0.4 [0.4, 2], 0.27	t[126.8]=3.011, 0.003
PP-BWS	wk6	1.2±0.4 [0.4, 2.1], 0.28	t[113.9]=2.949, 0.004
PP-noBWS	wk6	0.9±0.4 [0.1, 1.7], 0.21	t[119.3]=2.246, 0.027
TT	wk6	1.8±0.4 [1, 2.6], 0.41	t[127]=4.613, <0.001
PP-BWS	wk8	1.3±0.4 [0.5, 2.2], 0.3	t[113.4]=3.185, 0.002
PP-noBWS	wk8	1.6±0.4 [0.8, 2.4], 0.36	t[127.5]=4.068, <0.001
TT	wk8	2.4±0.4 [1.6, 3.2], 0.54	t[126.2]=6.102, <0.001
PP-BWS	wk10	0.8±0.5 [-0.2, 1.9], 0.21	t[59.5]=1.595, 0.116
PP-noBWS	wk10	1±0.5 [0, 2], 0.26	t[64.8]=2.085, 0.041
TT	wk10	2.1±0.5 [1.1, 3], 0.5	t[80.1]=4.44, <0.001

7.5 TUG

7.5.1 Between Group Difference in Change Score

Contrast	Timepoint	Difference±SE [95% CI], SMD	t[df], p-value
(PP-BWS) - (PP-noBWS)	wk2	-1.4±0.4 [-2.3, -0.4], -0.3	t[143.6]=-3.549, 0.002
(PP-BWS) - TT	wk2	-0.6±0.4 [-1.6, 0.3], -0.13	t[141.4]=-1.581, 0.116
(PP-noBWS) - TT	wk2	0.8±0.4 [-0.2, 1.7], 0.16	t[147.9]=1.983, 0.074
(PP-BWS) - (PP-noBWS)	wk4	-1.2±0.4 [-2.2, -0.3], -0.26	t[143]=-3.168, 0.006
(PP-BWS) - TT	wk4	-0.9±0.4 [-1.9, 0], -0.2	t[148.5]=-2.423, 0.025
(PP-noBWS) - TT	wk4	0.3±0.4 [-0.6, 1.2], 0.06	t[158.5]=0.818, 0.415
(PP-BWS) - (PP-noBWS)	wk6	-0.7±0.4 [-1.6, 0.3], -0.14	t[133.6]=-1.645, 0.172
(PP-BWS) - TT	wk6	-0.6±0.4 [-1.5, 0.3], -0.13	t[147]=-1.587, 0.172
(PP-noBWS) - TT	wk6	0±0.4 [-0.9, 1], 0.01	t[146.6]=0.124, 0.902
(PP-BWS) - (PP-noBWS)	wk8	-0.3±0.4 [-1.3, 0.6], -0.07	t[128.8]=-0.848, 0.463
(PP-BWS) - TT	wk8	0.3±0.4 [-0.7, 1.2], 0.06	t[145.9]=0.736, 0.463
(PP-noBWS) - TT	wk8	0.6±0.4 [-0.3, 1.6], 0.13	t[148.1]=1.637, 0.311
(PP-BWS) - (PP-noBWS)	wk10	-0.6±0.4 [-1.7, 0.5], -0.14	t[97.4]=-1.411, 0.485
(PP-BWS) - TT	wk10	-0.4±0.5 [-1.5, 0.7], -0.1	t[91.3]=-0.917, 0.543
(PP-noBWS) - TT	wk10	0.2±0.5 [-0.9, 1.3], 0.05	t[88.7]=0.471, 0.639

7.6 Within Group Change Score

Group	Timepoint	Estimate±SE [95% CI], SMD	t[df], p-value
PP-BWS	wk2	-1.5±0.3 [-2, -0.9], -0.45	t[132.7]=-5.197, <0.001
PP-noBWS	wk2	-0.1±0.3 [-0.6, 0.4], -0.03	t[151.3]=-0.366, 0.715
TT	wk2	-0.9±0.3 [-1.4, -0.3], -0.26	t[134]=-3.049, 0.003
PP-BWS	wk4	-1.7±0.3 [-2.2, -1.1], -0.51	t[132.7]=-5.833, <0.001
PP-noBWS	wk4	-0.4±0.3 [-1, 0.1], -0.13	t[151.8]=-1.581, 0.116
TT	wk4	-0.7±0.3 [-1.3, -0.2], -0.22	t[151.6]=-2.72, 0.007
PP-BWS	wk6	-1.8±0.3 [-2.4, -1.2], -0.54	t[129]=-6.162, <0.001
PP-noBWS	wk6	-1.1±0.3 [-1.7, -0.6], -0.35	t[128.3]=-3.921, <0.001
TT	wk6	-1.2±0.3 [-1.7, -0.6], -0.35	t[151.6]=-4.334, <0.001
PP-BWS	wk8	-1.9±0.3 [-2.5, -1.3], -0.58	t[127.5]=-6.496, <0.001
PP-noBWS	wk8	-1.5±0.3 [-2.1, -1], -0.47	t[132.3]=-5.449, <0.001
TT	wk8	-2.2±0.3 [-2.7, -1.6], -0.65	t[151.6]=-8.059, <0.001
PP-BWS	wk10	-2.1±0.3 [-2.8, -1.5], -0.68	t[90.5]=-6.465, <0.001
PP-noBWS	wk10	-1.5±0.4 [-2.2, -0.8], -0.52	t[65.8]=-4.202, <0.001
TT	wk10	-1.7±0.4 [-2.4, -1], -0.59	t[63.2]=-4.704, <0.001
