# Supplementary material for: Comparative effectiveness of lower body positive pressure and traditional treadmill training on adults with mild balance impairment
Source: Front Aging. 2025 Oct 22;6:1645026. doi: 10.3389/fragi.2025.1645026 (PMC12586058; doi:10.3389/fragi.2025.1645026)
Supplement: Supplementary file 2 [file DataSheet4.pdf]

AGT RCT G&B Analysis

Usman Rashid

13/09/2025

Contents

1 Statistical Analysis **2**

2 Results **2**
2.1 Static Tasks . . . 2
2.1.1 Postural Stability (PS) . . . 2
2.1.1.1 Model Diagnostics . . . 2
2.1.1.2 Anova . . . 2
2.1.1.3 Between Group Differences . . . 3
2.1.1.4 Change over Time . . . 5
2.1.2 Postural Stability Mediolateral (PS.ML) . . . 6
2.1.2.1 Model Diagnostics . . . 6
2.1.2.2 Anova . . . 6
2.1.2.3 Change over Time . . . 7
2.1.3 Postural Stability Anterior-posterior (PS.AP) . . . 8
2.1.3.1 Model Diagnostics . . . 8
2.1.3.2 Anova . . . 8
2.1.3.3 Change over Time . . . 9
2.2 Gait Tasks . . . 10
2.2.1 Gait Symmetry . . . 10
2.2.1.1 Model Diagnostics . . . 10
2.2.1.2 Anova . . . 10
2.2.1.3 Change over Time . . . 11
2.2.2 Walking Speed . . . 12
2.2.2.1 Model Diagnostics . . . 12
2.2.2.2 Anova . . . 12
2.2.2.3 Change over Time . . . 13
2.2.3 Step length . . . 14
2.2.3.1 Model Diagnostics . . . 14
2.2.3.2 Anova . . . 14
2.2.3.3 Change over Time . . . 15
2.2.4 Step time . . . 16
2.2.4.1 Model Diagnostics . . . 16
2.2.4.2 Anova . . . 16
2.2.4.3 Change over Time . . . 17

3 Sensitivity Analysis by Multiple-imputations **18**
3.1 Postural Stability (PS) . . . 18
3.1.1 Between Group Difference in Change Score . . . 18

1 Statistical Analysis

Statistical models are fitted to change scores (Post- - Pre-intervention values). Thus, a positive score implies that the post-intervention value is larger than the pre-intervention value. Benjamini-Hochberg adjustment is applied for between group differences.

2 Results

2.1 Static Tasks

2.1.1 Postural Stability (PS)

```
## boundary (singular) fit: see help('isSingular')
```

2.1.1.1 Model Diagnostics

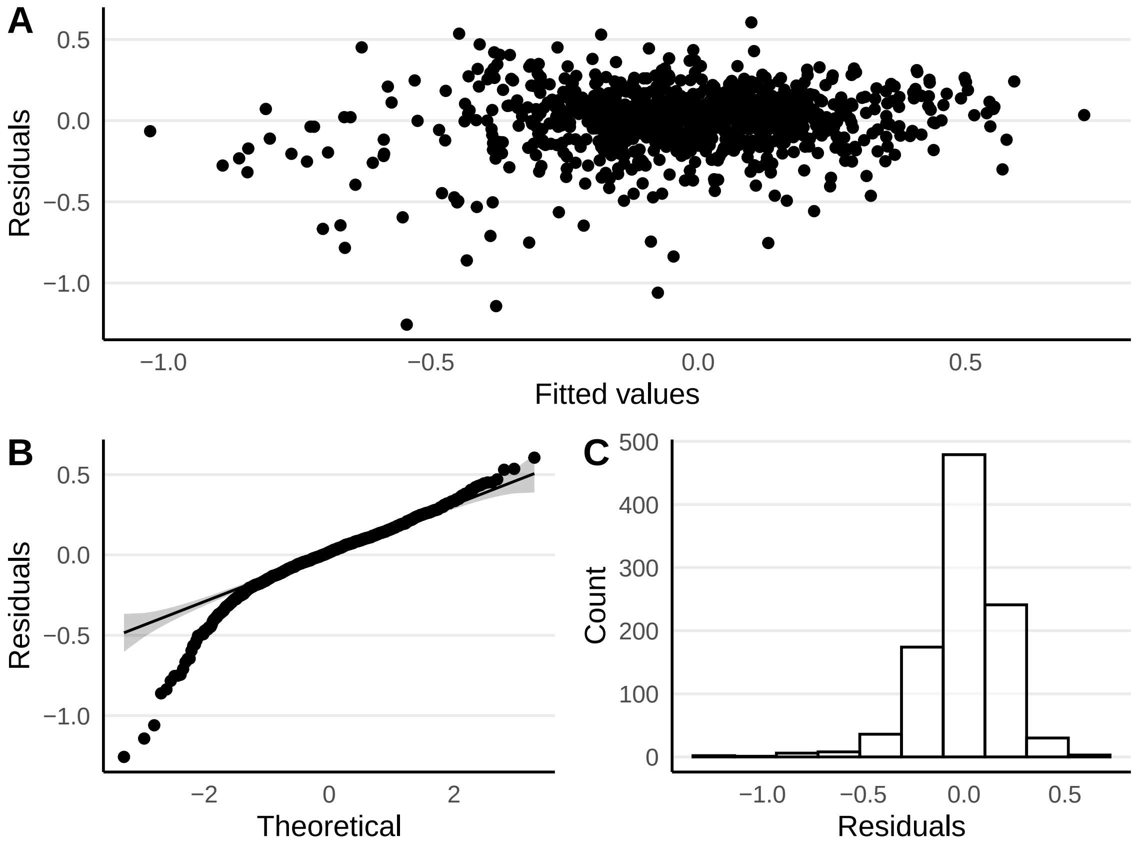

2.1.1.2 Anova

```
Analysis of Deviance Table (Type III Wald chisquare tests)

Response: (PS.R - PS.R.pre)
                              Chisq Df Pr(>Chisq)
```

```
(Intercept)           17.2724  1  3.239e-05 ***
PS.R.pre             240.9010  1  < 2.2e-16 ***
Age_years             15.8834  1  6.737e-05 ***
Height_cm              1.4059  1   0.235742
BMI                    0.6999  1   0.402808
Task                   7.2842  1   0.006957 **
Qual                   1.6695  1   0.196330
Group                  2.8752  2   0.237499
Week                  11.3182  4   0.023211 *
Task:Qual              4.5488  1   0.032942 *
Task:Group             1.4483  2   0.484726
Qual:Group             1.5638  2   0.457529
Task:Week              4.0737  4   0.396124
Qual:Week              0.7636  4   0.943255
Group:Week             5.7895  8   0.670797
Task:Qual:Group        4.4529  2   0.107911
Task:Qual:Week        10.7700  4   0.029274 *
Task:Group:Week        3.2993  8   0.914197
Qual:Group:Week        2.5912  8   0.957342
Task:Qual:Group:Week  10.7401  8   0.216865
---
Signif. codes:  0 '***' 0.001 '**' 0.01 '*' 0.05 '.' 0.1 ' ' 1
```

2.1.1.3 Between Group Differences

Contrast	Qual	Week	Task	Difference±SE [95% CI], SMD	t[df], p-value
(PP-BWS) - (PP-noBWS)	EC	wk2	Compliant	0±0.1 [-0.2, 0.3], 0	t[121.2]=0.066, 0.947
(PP-BWS) - TT	EC	wk2	Compliant	-0.2±0.1 [-0.4, 0.1], -0.1	t[123.4]=-1.427, 0.234
(PP-noBWS) - TT	EC	wk2	Compliant	-0.2±0.1 [-0.4, 0.1], -0.1	t[121]=-1.524, 0.234
(PP-BWS) - (PP-noBWS)	EO	wk2	Compliant	0±0.1 [-0.3, 0.2], 0	t[121.2]=-0.293, 0.77
(PP-BWS) - TT	EO	wk2	Compliant	-0.1±0.1 [-0.3, 0.2], -0.1	t[123.3]=-0.612, 0.77
(PP-noBWS) - TT	EO	wk2	Compliant	0±0.1 [-0.3, 0.2], 0	t[121]=-0.337, 0.77
(PP-BWS) - (PP-noBWS)	EC	wk4	Compliant	-0.01±0.09 [-0.23, 0.21], 0	t[189.3]=-0.133, 0.894
(PP-BWS) - TT	EC	wk4	Compliant	-0.09±0.09 [-0.31, 0.13], -0.1	t[187]=-0.976, 0.606
(PP-noBWS) - TT	EC	wk4	Compliant	-0.08±0.09 [-0.3, 0.14], -0.1	t[193.6]=-0.836, 0.606
(PP-BWS) - (PP-noBWS)	EO	wk4	Compliant	0.07±0.09 [-0.14, 0.29], 0.1	t[189.5]=0.824, 0.617
(PP-BWS) - TT	EO	wk4	Compliant	-0.04±0.09 [-0.26, 0.17], 0	t[186.9]=-0.496, 0.621
(PP-noBWS) - TT	EO	wk4	Compliant	-0.12±0.09 [-0.34, 0.1], -0.1	t[194]=-1.302, 0.584
(PP-BWS) - (PP-noBWS)	EC	wk6	Compliant	-0.01±0.09 [-0.23, 0.21], 0	t[193.5]=-0.085, 0.932
(PP-BWS) - TT	EC	wk6	Compliant	-0.05±0.09 [-0.27, 0.16], 0	t[186.6]=-0.607, 0.91
(PP-noBWS) - TT	EC	wk6	Compliant	-0.05±0.09 [-0.26, 0.17], 0	t[193.1]=-0.515, 0.91
(PP-BWS) - (PP-noBWS)	EO	wk6	Compliant	-0.01±0.09 [-0.23, 0.21], 0	t[193.5]=-0.129, 0.897
(PP-BWS) - TT	EO	wk6	Compliant	-0.07±0.09 [-0.28, 0.15], -0.1	t[186.5]=-0.779, 0.783
(PP-noBWS) - TT	EO	wk6	Compliant	-0.06±0.09 [-0.27, 0.16], 0	t[193.1]=-0.641, 0.783
(PP-BWS) - (PP-noBWS)	EC	wk8	Compliant	0.2±0.1 [-0.1, 0.4], 0.2	t[129.1]=1.781, 0.134
(PP-BWS) - TT	EC	wk8	Compliant	0±0.1 [-0.2, 0.3], 0	t[123.8]=0.081, 0.935

Contrast	Qual	Week	Task	Difference±SE [95% CI], SMD	t[df], p-value
(PP-noBWS) - TT	EC	wk8	Compliant	-0.2±0.1 [-0.4, 0.1], -0.2	t[124.9]=-1.714, 0.134
(PP-BWS) - (PP-noBWS)	EO	wk8	Compliant	0.2±0.1 [0, 0.5], 0.2	t[129.1]=2.035, 0.066
(PP-BWS) - TT	EO	wk8	Compliant	0±0.1 [-0.3, 0.3], 0	t[123.8]=-0.042, 0.966
(PP-noBWS) - TT	EO	wk8	Compliant	-0.2±0.1 [-0.5, 0], -0.2	t[124.8]=-2.094, 0.066
(PP-BWS) - (PP-noBWS)	EC	wk10	Compliant	0.1±0.1 [-0.2, 0.4], 0.1	t[198.8]=0.914, 0.79
(PP-BWS) - TT	EC	wk10	Compliant	0.1±0.1 [-0.2, 0.3], 0	t[205.5]=0.634, 0.79
(PP-noBWS) - TT	EC	wk10	Compliant	0±0.1 [-0.3, 0.3], 0	t[206.5]=-0.225, 0.822
(PP-BWS) - (PP-noBWS)	EO	wk10	Compliant	0±0.1 [-0.2, 0.3], 0	t[198.9]=0.422, 0.961
(PP-BWS) - TT	EO	wk10	Compliant	0±0.1 [-0.3, 0.3], 0	t[205.4]=0.05, 0.961
(PP-noBWS) - TT	EO	wk10	Compliant	0±0.1 [-0.3, 0.2], 0	t[206.6]=-0.342, 0.961
(PP-BWS) - (PP-noBWS)	EC	wk2	Firm	0±0.1 [-0.3, 0.2], 0	t[124.5]=-0.118, 0.906
(PP-BWS) - TT	EC	wk2	Firm	-0.1±0.1 [-0.3, 0.2], 0	t[123.3]=-0.525, 0.906
(PP-noBWS) - TT	EC	wk2	Firm	0±0.1 [-0.3, 0.2], 0	t[123.9]=-0.416, 0.906
(PP-BWS) - (PP-noBWS)	EO	wk2	Firm	0.1±0.1 [-0.2, 0.3], 0.1	t[121.3]=0.761, 0.448
(PP-BWS) - TT	EO	wk2	Firm	-0.1±0.1 [-0.4, 0.1], -0.1	t[127.4]=-1.345, 0.272
(PP-noBWS) - TT	EO	wk2	Firm	-0.2±0.1 [-0.5, 0], -0.2	t[125]=-2.117, 0.109
(PP-BWS) - (PP-noBWS)	EC	wk4	Firm	-0.01±0.09 [-0.22, 0.21], 0	t[183.6]=-0.067, 0.947
(PP-BWS) - TT	EC	wk4	Firm	0.04±0.09 [-0.18, 0.26], 0	t[181.9]=0.465, 0.947
(PP-noBWS) - TT	EC	wk4	Firm	0.05±0.09 [-0.17, 0.27], 0	t[194.2]=0.522, 0.947
(PP-BWS) - (PP-noBWS)	EO	wk4	Firm	-0.25±0.09 [-0.47, -0.04], -0.2	t[183.7]=-2.831, 0.008*
(PP-BWS) - TT	EO	wk4	Firm	-0.34±0.09 [-0.56, -0.13], -0.3	t[182.1]=-3.817, <0.001*
(PP-noBWS) - TT	EO	wk4	Firm	-0.09±0.09 [-0.31, 0.13], -0.1	t[194.7]=-0.988, 0.325
(PP-BWS) - (PP-noBWS)	EC	wk6	Firm	0.09±0.09 [-0.13, 0.31], 0.1	t[193.5]=0.992, 0.562
(PP-BWS) - TT	EC	wk6	Firm	0.08±0.09 [-0.14, 0.29], 0.1	t[186.7]=0.89, 0.562
(PP-noBWS) - TT	EC	wk6	Firm	-0.01±0.09 [-0.23, 0.21], 0	t[193.1]=-0.112, 0.911
(PP-BWS) - (PP-noBWS)	EO	wk6	Firm	-0.06±0.09 [-0.28, 0.16], 0	t[193.6]=-0.679, 0.498
(PP-BWS) - TT	EO	wk6	Firm	-0.19±0.09 [-0.41, 0.02], -0.2	t[186.8]=-2.161, 0.096
(PP-noBWS) - TT	EO	wk6	Firm	-0.13±0.09 [-0.35, 0.09], -0.1	t[193.1]=-1.459, 0.219
(PP-BWS) - (PP-noBWS)	EC	wk8	Firm	0.2±0.1 [0, 0.5], 0.2	t[129.2]=2.054, 0.066
(PP-BWS) - TT	EC	wk8	Firm	0±0.1 [-0.3, 0.3], 0	t[123.8]=0.04, 0.968
(PP-noBWS) - TT	EC	wk8	Firm	-0.2±0.1 [-0.5, 0], -0.2	t[124.7]=-2.032, 0.066
(PP-BWS) - (PP-noBWS)	EO	wk8	Firm	0.1±0.1 [-0.1, 0.4], 0.1	t[129.2]=1.328, 0.28
(PP-BWS) - TT	EO	wk8	Firm	-0.1±0.1 [-0.3, 0.2], 0	t[123.9]=-0.554, 0.58
(PP-noBWS) - TT	EO	wk8	Firm	-0.2±0.1 [-0.5, 0.1], -0.2	t[124.8]=-1.893, 0.182
(PP-BWS) - (PP-noBWS)	EC	wk10	Firm	0.1±0.1 [-0.2, 0.3], 0	t[199.4]=0.479, 0.961
(PP-BWS) - TT	EC	wk10	Firm	0±0.1 [-0.3, 0.3], 0	t[206]=0.049, 0.961
(PP-noBWS) - TT	EC	wk10	Firm	0±0.1 [-0.3, 0.2], 0	t[206.5]=-0.396, 0.961
(PP-BWS) - (PP-noBWS)	EO	wk10	Firm	0±0.1 [-0.2, 0.3], 0	t[199.7]=0.281, 0.779

Contrast	Qual	Week	Task	Difference±SE [95% CI], SMD	t[df], p-value
(PP-BWS) - TT	EO	wk10	Firm	0.1±0.1 [-0.2, 0.3], 0	t[205.6]=0.559, 0.779
(PP-noBWS) - TT	EO	wk10	Firm	0±0.1 [-0.2, 0.3], 0	t[206.6]=0.287, 0.779

2.1.1.4 Change over Time

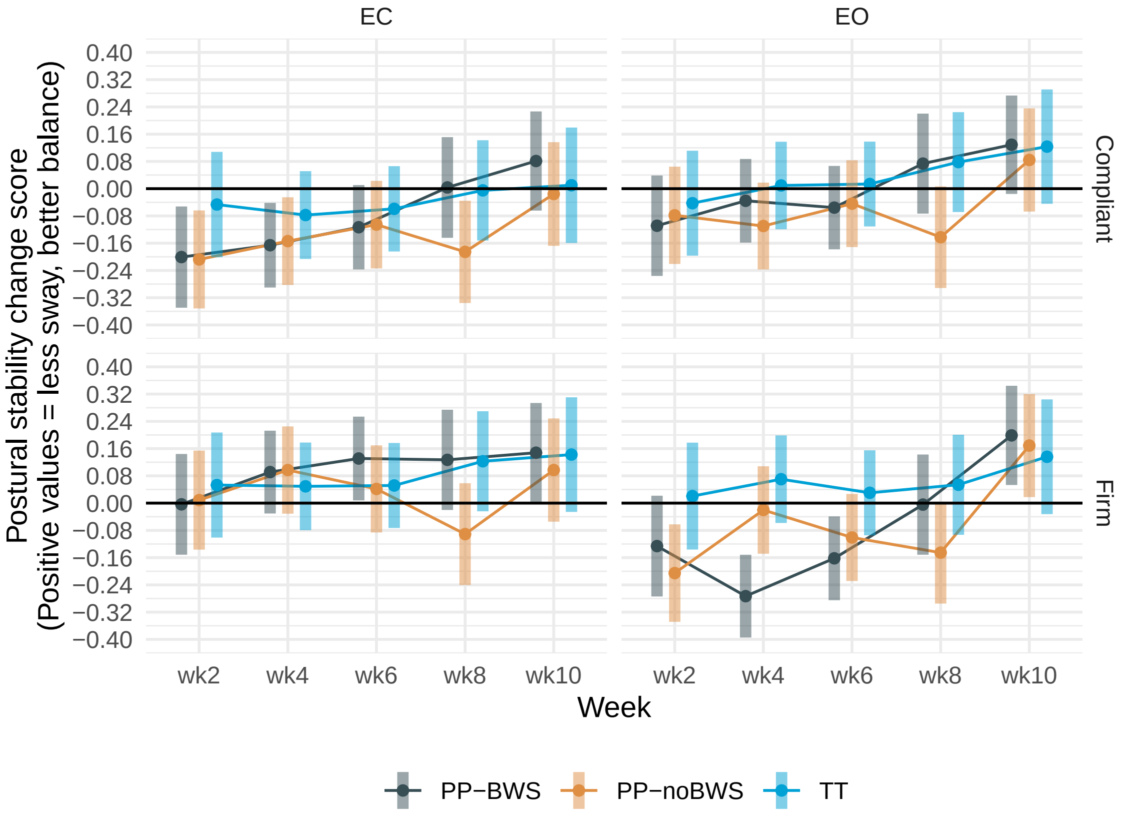

2.1.2 Postural Stability Mediolateral (PS.ML)

```
## boundary (singular) fit: see help('isSingular')
```

2.1.2.1 Model Diagnostics

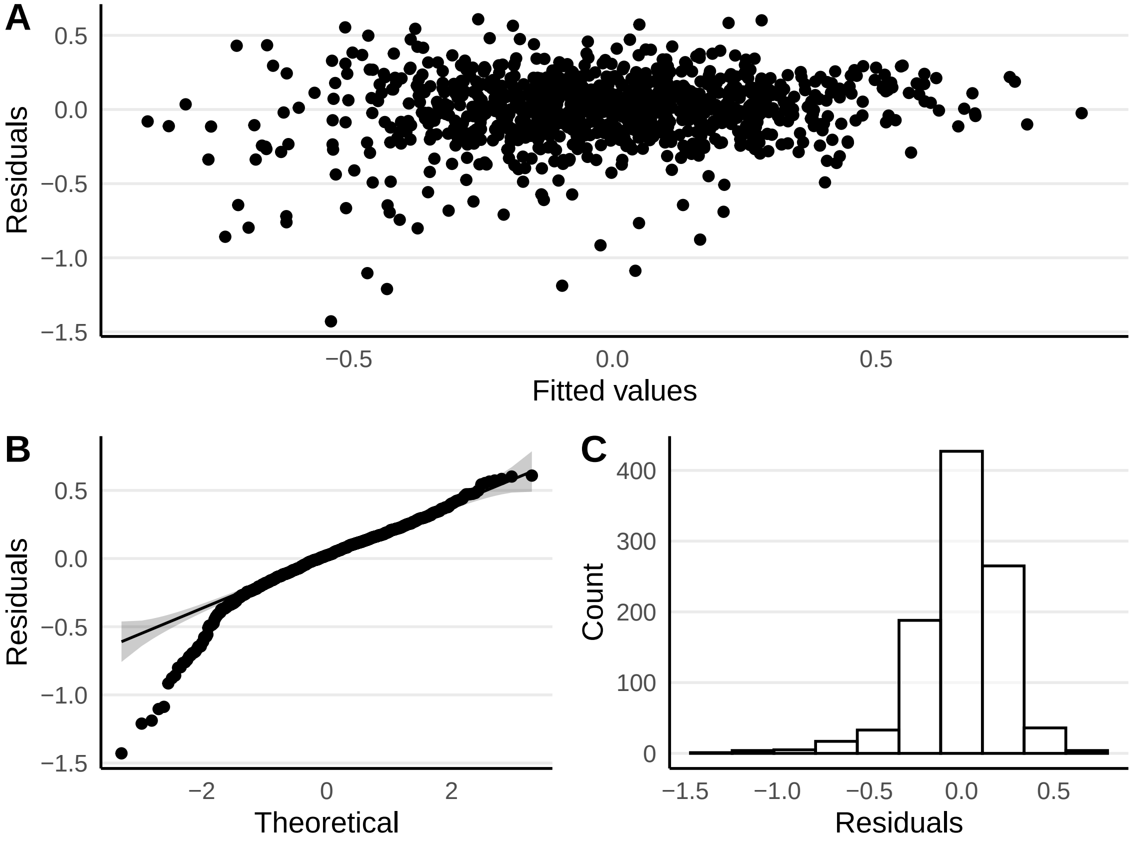

2.1.2.2 Anova

```
Analysis of Deviance Table (Type III Wald chisquare tests)

Response: (PS.ML - PS.ML.pre)
                       Chisq Df Pr(>Chisq)
(Intercept)          17.7829  1  2.476e-05 ***
PS.ML.pre           279.4273  1  < 2.2e-16 ***
Age_years            11.8955  1  0.0005627 ***
Height_cm             2.8490  1  0.0914285 .
BMI                   1.1015  1  0.2939306
Task                  4.2839  1  0.0384746 *
Qual                  0.8756  1  0.3494088
Group                 1.9617  2  0.3749932
Week                  7.1436  4  0.1284936
Task:Qual             3.5993  1  0.0578031 .
Task:Group            2.1505  2  0.3412083
Qual:Group            2.1277  2  0.3451203
```

```
Task:Week               1.8053  4  0.7715177
Qual:Week               1.4110  4  0.8422797
Group:Week              3.1732  8  0.9230228
Task:Qual:Group         6.3127  2  0.0425809 *
Task:Qual:Week          8.8519  4  0.0649088 .
Task:Group:Week         3.5736  8  0.8934043
Qual:Group:Week         3.7333  8  0.8803517
Task:Qual:Group:Week   10.6858  8  0.2201482
---
Signif. codes:  0 '***' 0.001 '**' 0.01 '*' 0.05 '.' 0.1 ' ' 1
```

2.1.2.3 Change over Time

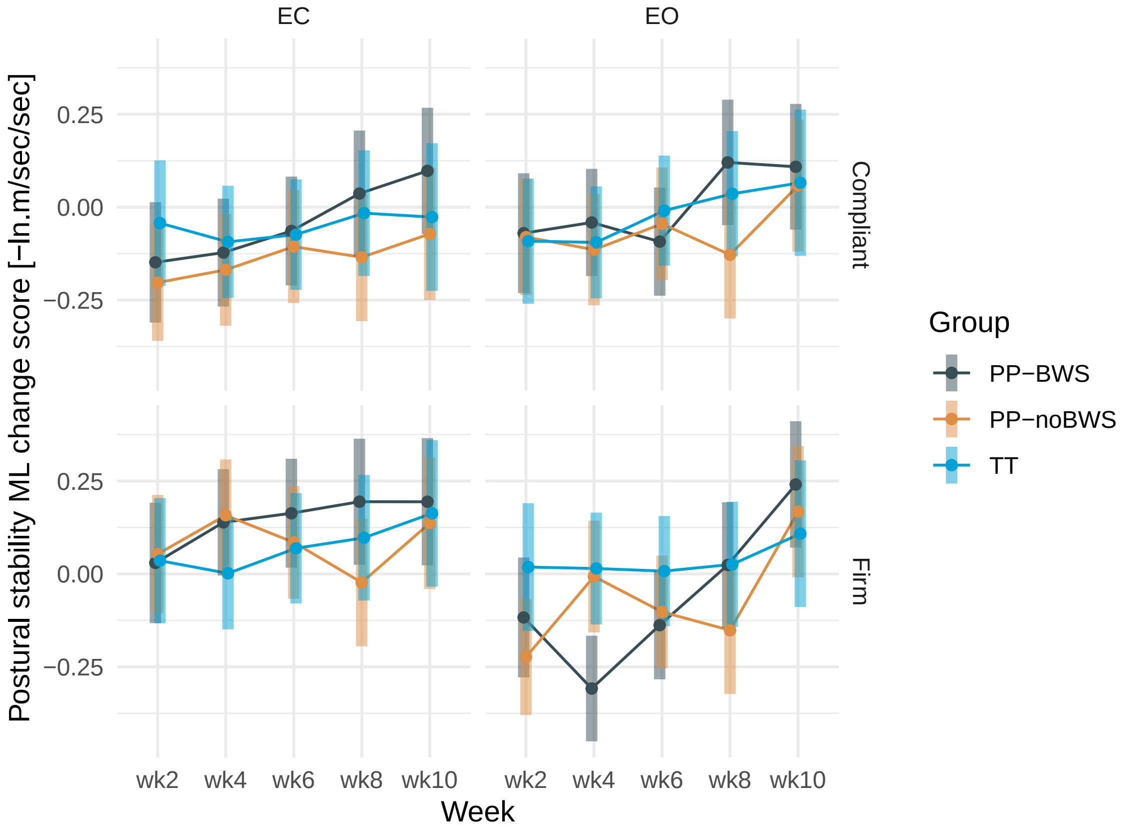

2.1.3 Postural Stability Anterior-posterior (PS.AP)

```
## boundary (singular) fit: see help('isSingular')
```

2.1.3.1 Model Diagnostics

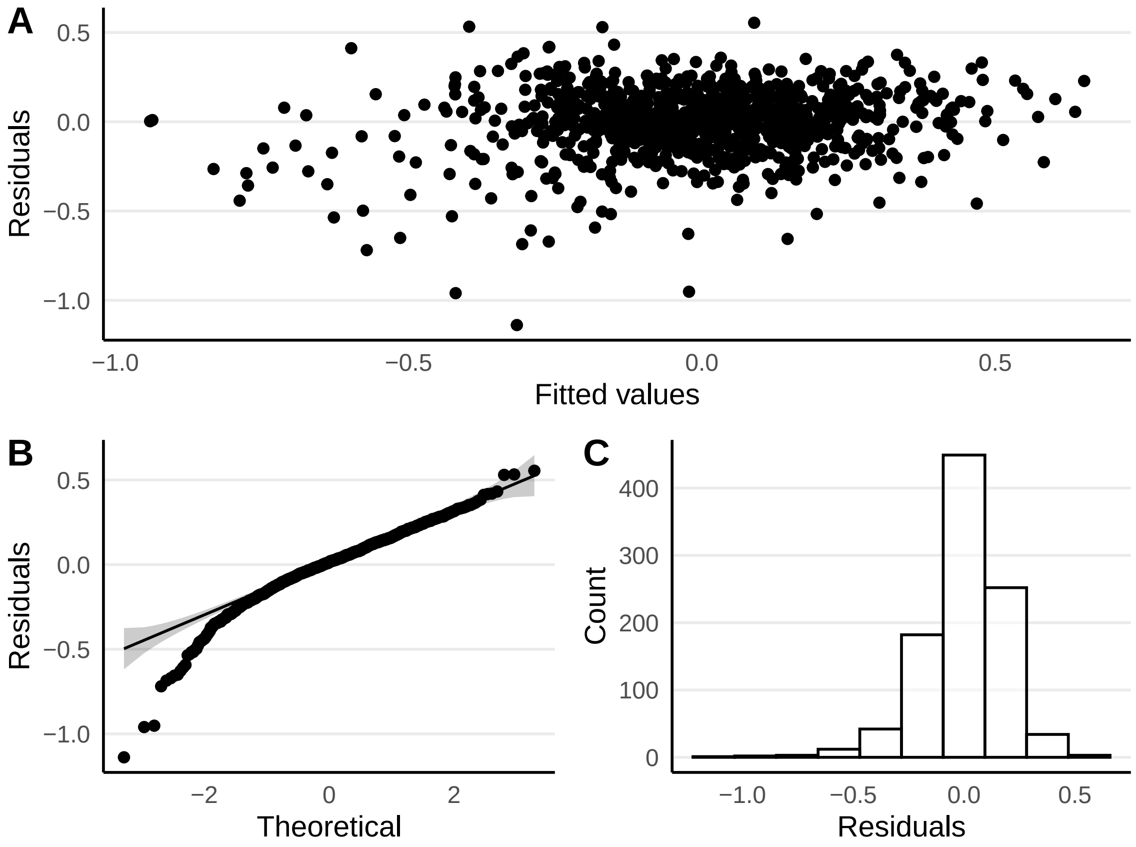

2.1.3.2 Anova

```
Analysis of Deviance Table (Type III Wald chisquare tests)

Response: (PS.AP - PS.AP.pre)
                        Chisq Df Pr(>Chisq)
(Intercept)           28.9264  1  7.518e-08 ***
PS.AP.pre            288.8861  1  < 2.2e-16 ***
Age_years              9.2682  1   0.002332 **
Height_cm              0.2094  1   0.647206
BMI                    0.1162  1   0.733217
Task                   6.2048  1   0.012740 *
Qual                   2.4072  1   0.120781
Group                  2.2292  2   0.328054
Week                  10.5489  4   0.032130 *
Task:Qual              3.6936  1   0.054623 .
Task:Group             0.6153  2   0.735164
Qual:Group             0.6422  2   0.725341
```

```
Task:Week               4.5044  4   0.342027
Qual:Week               1.1814  4   0.881153
Group:Week              4.2068  8   0.838000
Task:Qual:Group         2.5506  2   0.279344
Task:Qual:Week         10.8002  4   0.028904 *
Task:Group:Week         3.1886  8   0.921973
Qual:Group:Week         2.5483  8   0.959438
Task:Qual:Group:Week    9.4345  8   0.306977
---
Signif. codes:  0 '***' 0.001 '**' 0.01 '*' 0.05 '.' 0.1 ' ' 1
```

2.1.3.3 Change over Time

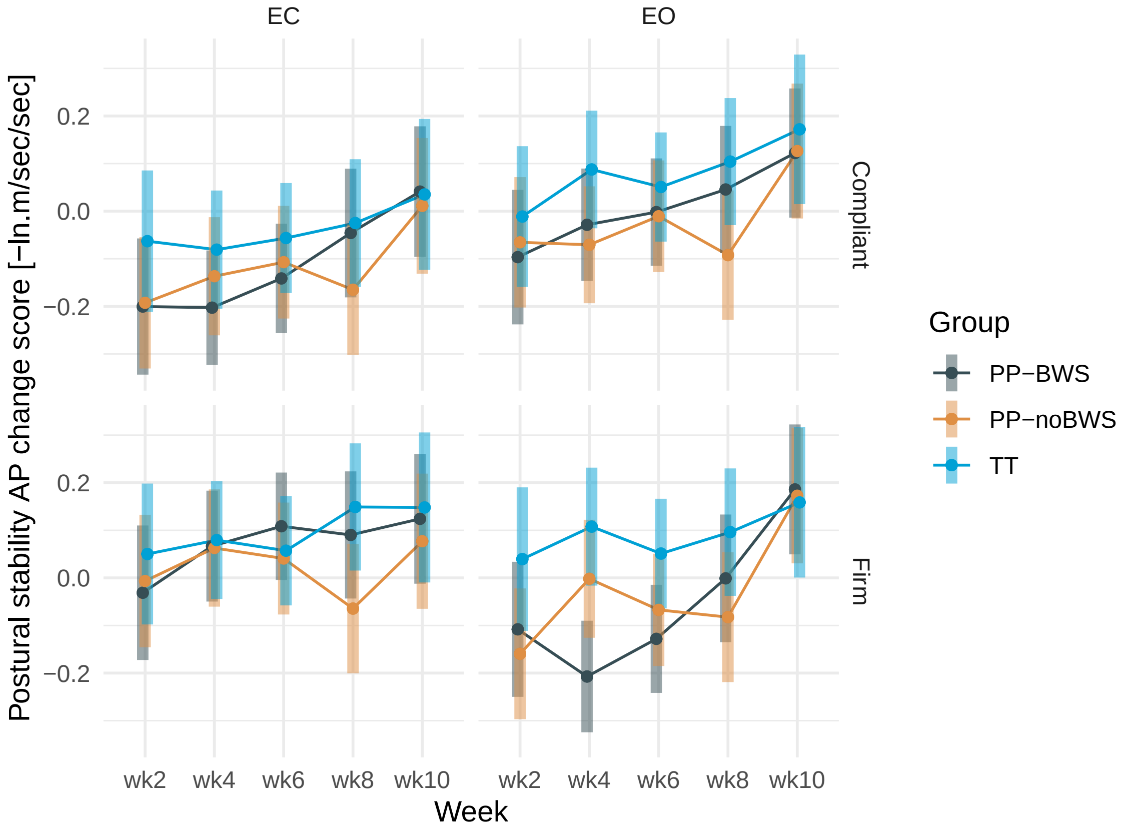

2.2 Gait Tasks

2.2.1 Gait Symmetry

```
## boundary (singular) fit: see help('isSingular')
```

2.2.1.1 Model Diagnostics

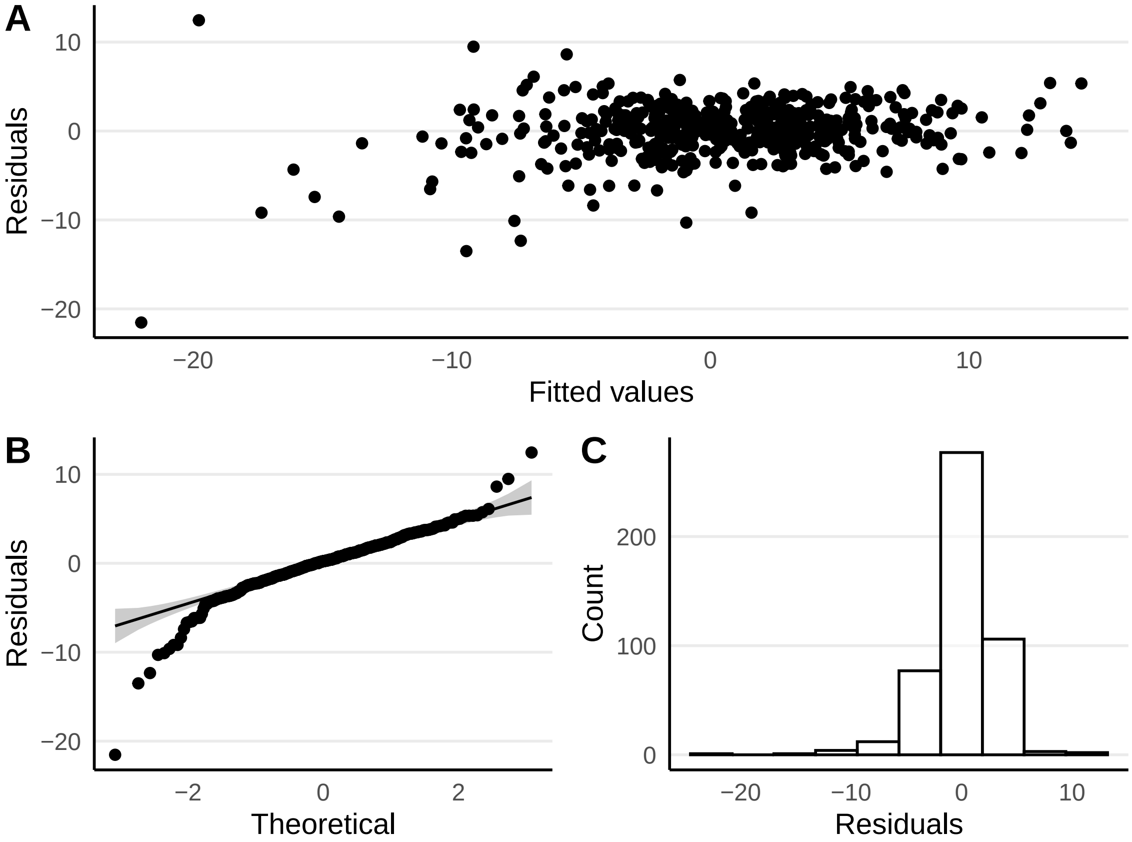

2.2.1.2 Anova

```
Analysis of Deviance Table (Type III Wald chisquare tests)

Response: (Gait.symmetry - Gait.symmetry.pre)
                     Chisq Df Pr(>Chisq)
(Intercept)        18.8211  1  1.436e-05 ***
Gait.symmetry.pre 186.5114  1  < 2.2e-16 ***
Age_years           2.4710  1    0.11596
Height_cm           0.0261  1    0.87157
BMI                 0.2407  1    0.62368
Qual                1.2305  1    0.26730
Group               0.2584  2    0.87880
Week               10.5750  4    0.03178 *
Qual:Group          0.0535  2    0.97361
Qual:Week           6.7164  4    0.15166
```

```
Group:Week          8.3407  8    0.40092
Qual:Group:Week     7.9299  8    0.44035
---
Signif. codes:  0 '***' 0.001 '**' 0.01 '*' 0.05 '.' 0.1 ' ' 1
```

2.2.1.3 Change over Time

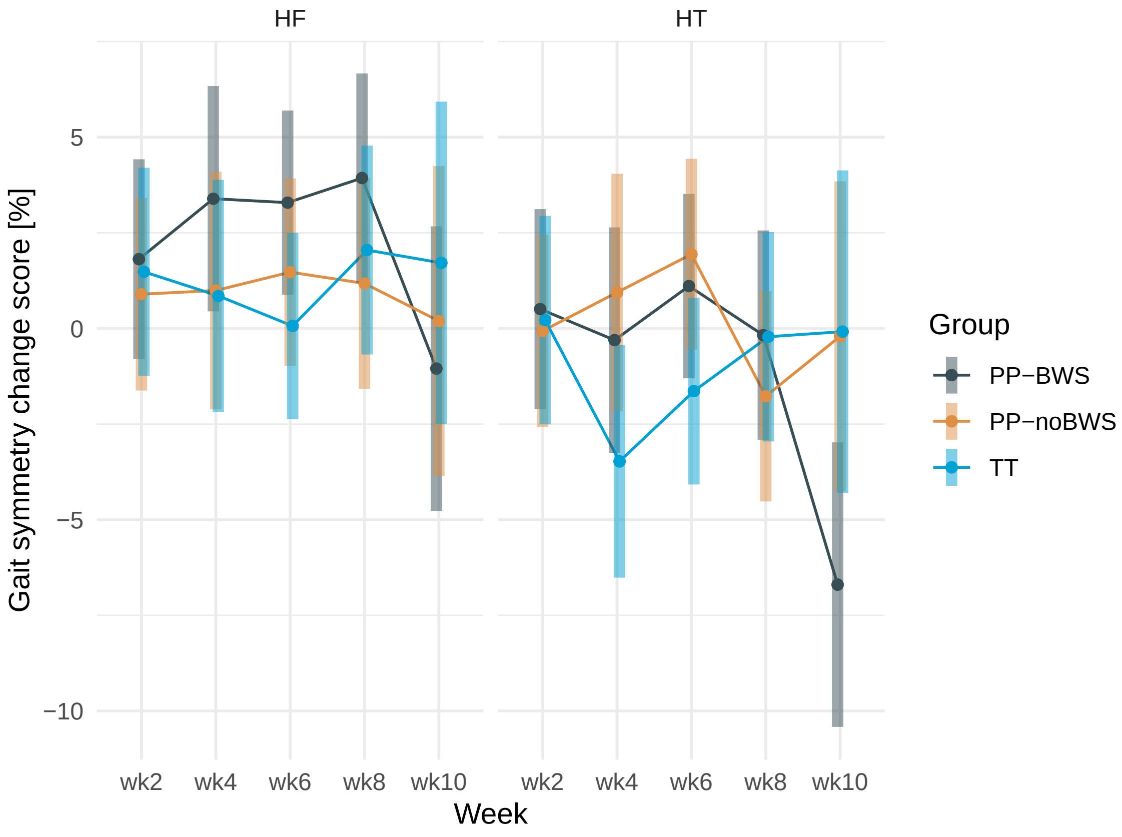

2.2.2 Walking Speed

2.2.2.1 Model Diagnostics

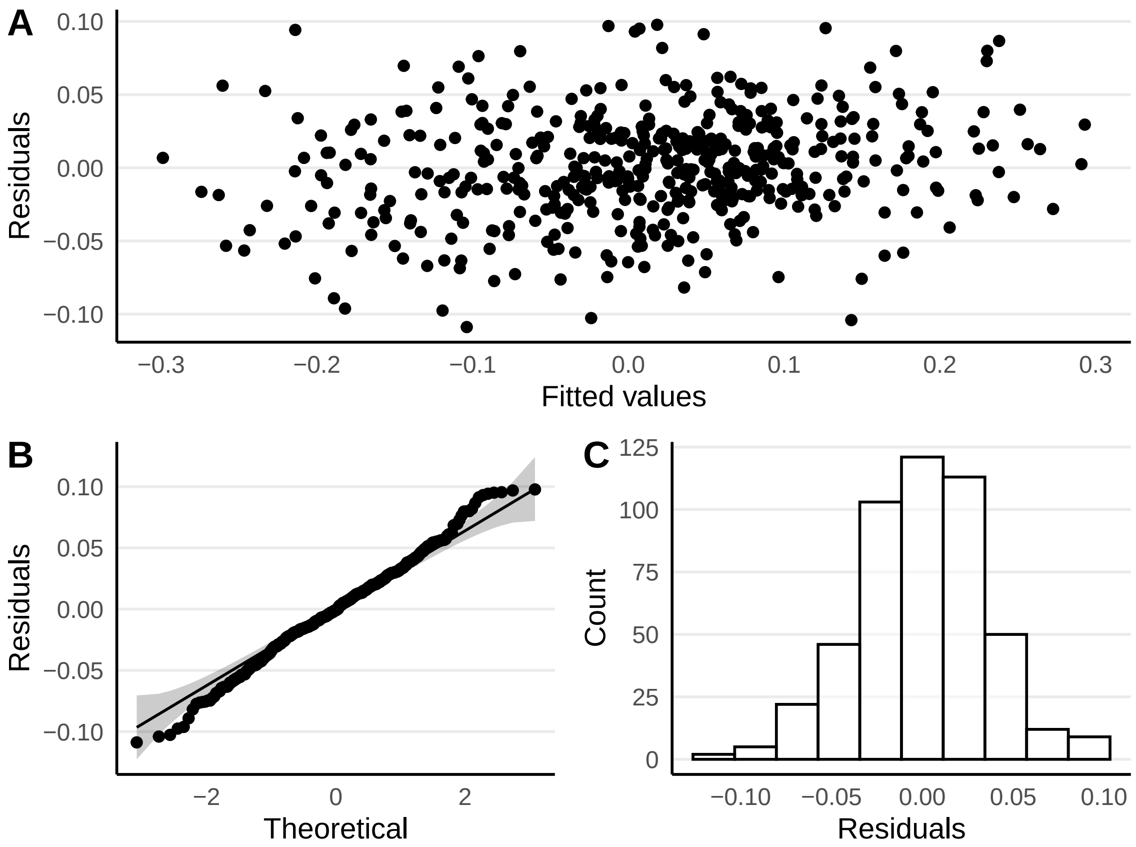

2.2.2.2 Anova

```
Analysis of Deviance Table (Type III Wald chisquare tests)

Response: (Step.velocity - Step.velocity.pre)
                      Chisq Df Pr(>Chisq)
(Intercept)          1.0985  1    0.29459
Step.velocity.pre 184.7184  1    < 2e-16 ***
Age_years            0.3313  1    0.56492
Height_cm            2.0076  1    0.15651
BMI                  0.9383  1    0.33273
Qual                 2.5692  1    0.10896
Group                0.6858  2    0.70970
Week                 9.8500  4    0.04303 *
Qual:Group           0.8639  2    0.64923
Qual:Week            1.8403  4    0.76510
Group:Week           8.9640  8    0.34534
Qual:Group:Week      1.7804  8    0.98702
---
Signif. codes:  0 '***' 0.001 '**' 0.01 '*' 0.05 '.' 0.1 ' ' 1
```

2.2.2.3 Change over Time

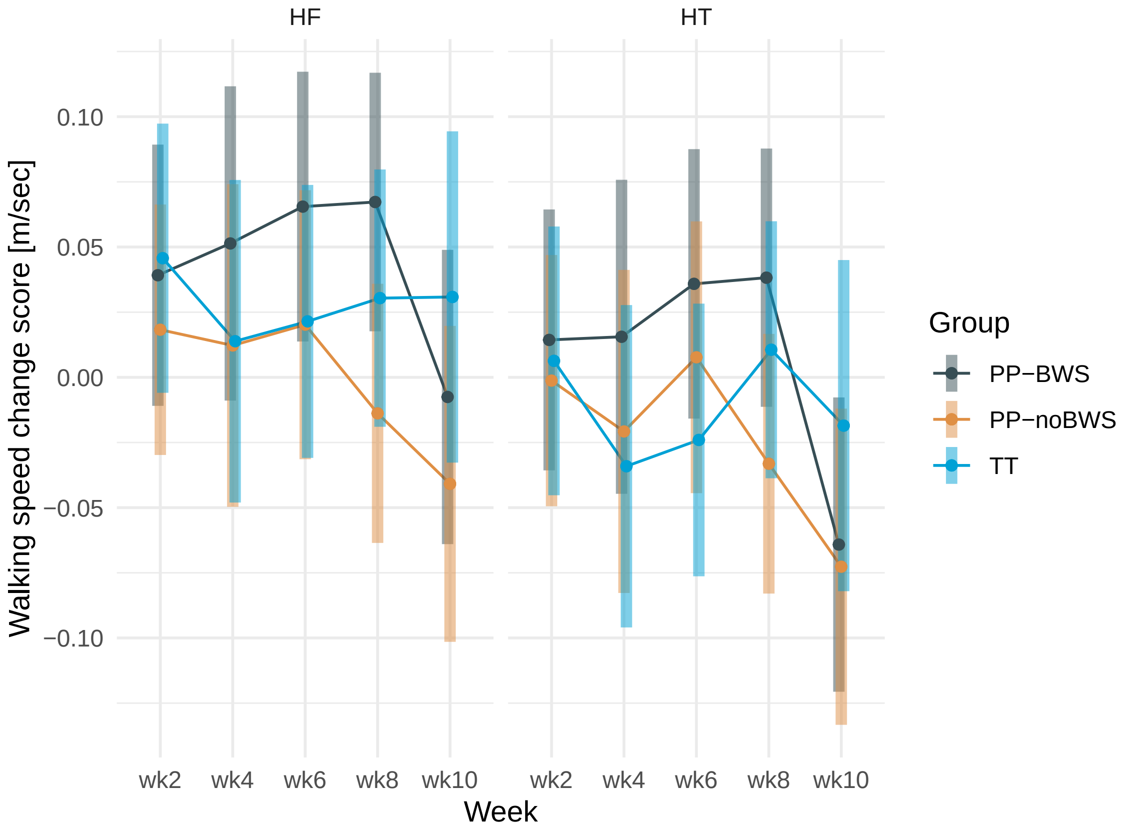

2.2.3 Step length

2.2.3.1 Model Diagnostics

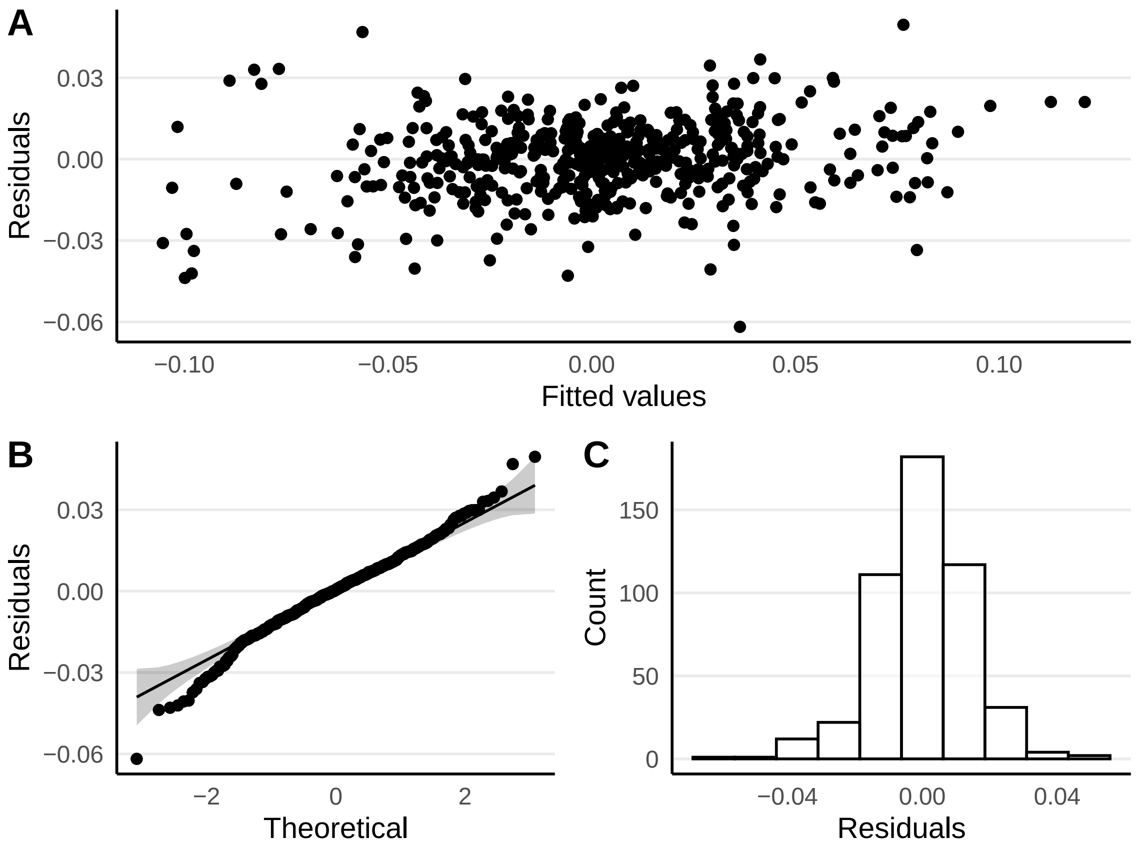

2.2.3.2 Anova

```
Analysis of Deviance Table (Type III Wald chisquare tests)

Response: (Step.length - Step.length.pre)
                    Chisq Df Pr(>Chisq)
(Intercept)        0.4648  1   0.495405
Step.length.pre  137.4548  1  < 2.2e-16 ***
Age_years          0.6666  1   0.414247
Height_cm         12.1573  1   0.000489 ***
BMI                0.2310  1   0.630812
Qual               0.7470  1   0.387437
Group              0.7171  2   0.698691
Week               6.5048  4   0.164489
Qual:Group         0.0250  2   0.987564
Qual:Week          0.9179  4   0.921979
Group:Week         6.1881  8   0.626172
Qual:Group:Week    3.0386  8   0.931910
---
Signif. codes:  0 '***' 0.001 '**' 0.01 '*' 0.05 '.' 0.1 ' ' 1
```

2.2.3.3 Change over Time

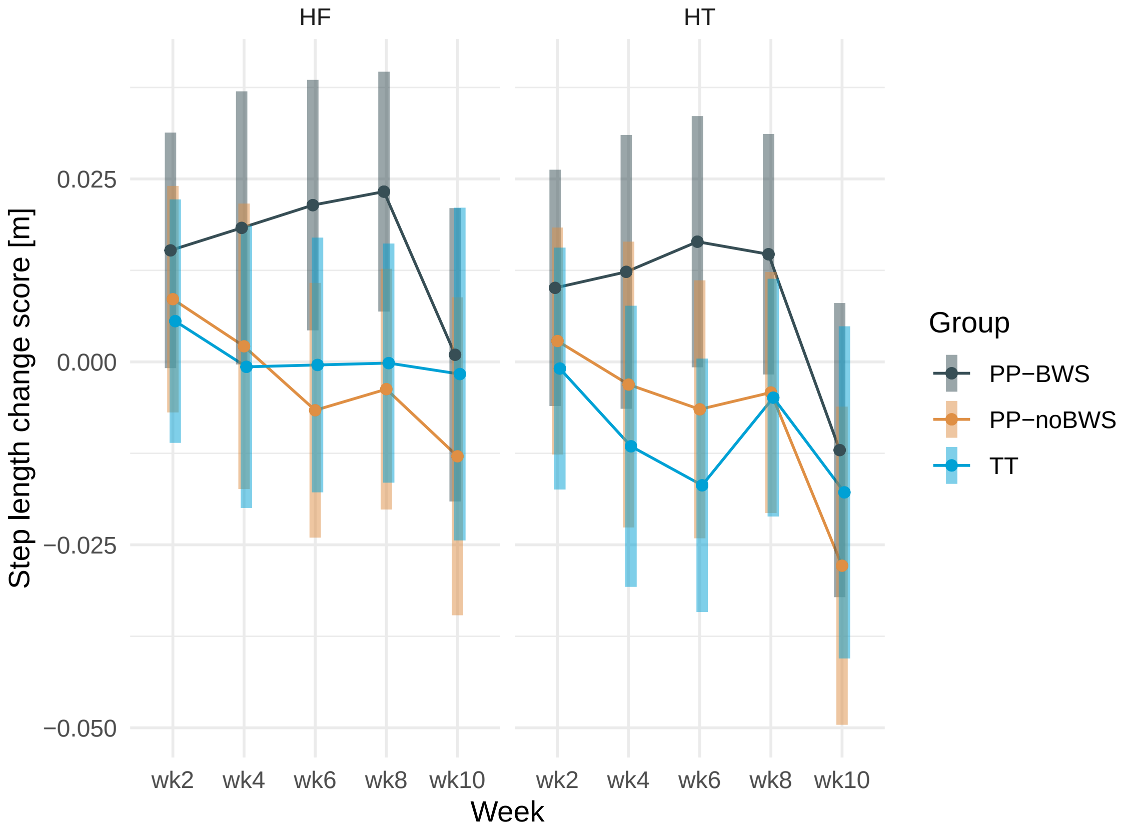

2.2.4 Step time

```
## boundary (singular) fit: see help('isSingular')
```

2.2.4.1 Model Diagnostics

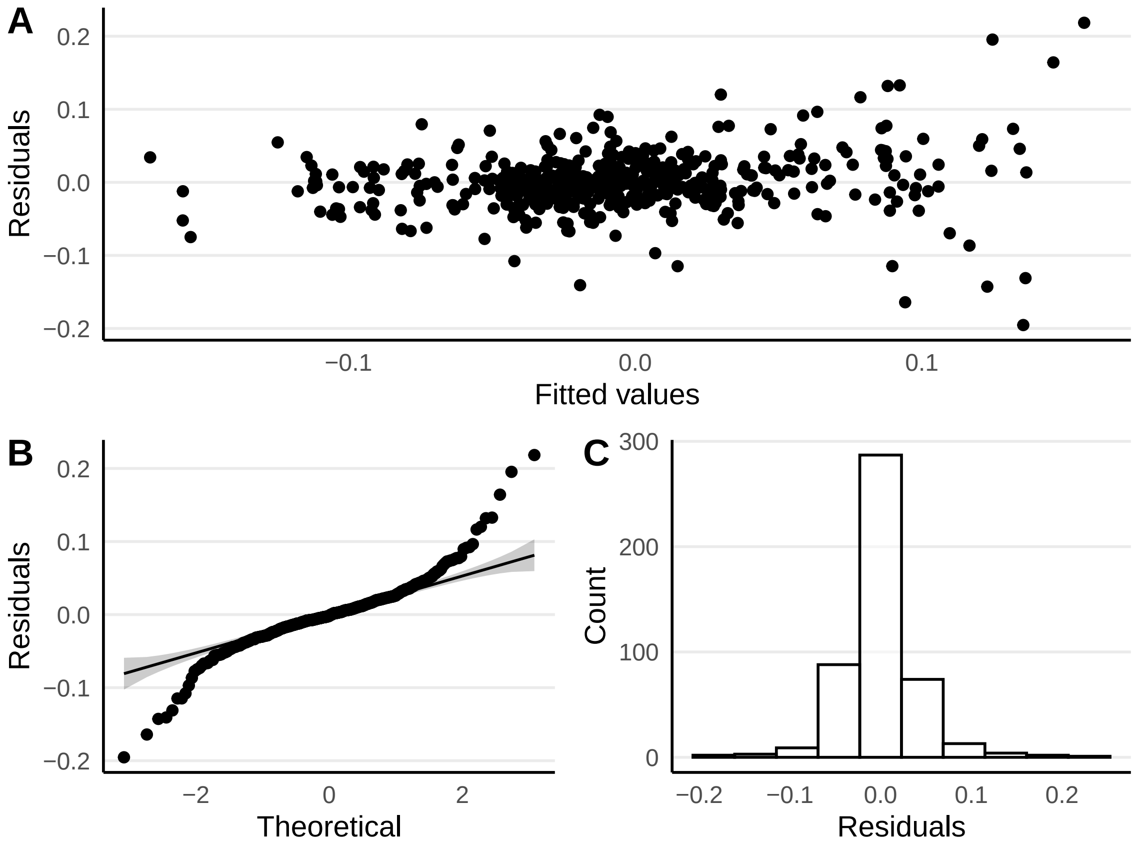

2.2.4.2 Anova

```
Analysis of Deviance Table (Type III Wald chisquare tests)

Response: (Step.time - Step.time.pre)
                  Chisq Df Pr(>Chisq)
(Intercept)      1.6139  1     0.2039
Step.length.pre 0.0460  1     0.8301
Age_years        0.0146  1     0.9040
Height_cm        0.9914  1     0.3194
BMI              2.6013  1     0.1068
Qual             0.0000  1     0.9962
Group            0.1188  2     0.9423
Week             1.4331  4     0.8384
Qual:Group       0.0103  2     0.9949
Qual:Week        1.2126  4     0.8760
Group:Week       5.1149  8     0.7452
Qual:Group:Week 1.1113  8     0.9974
```

2.2.4.3 Change over Time

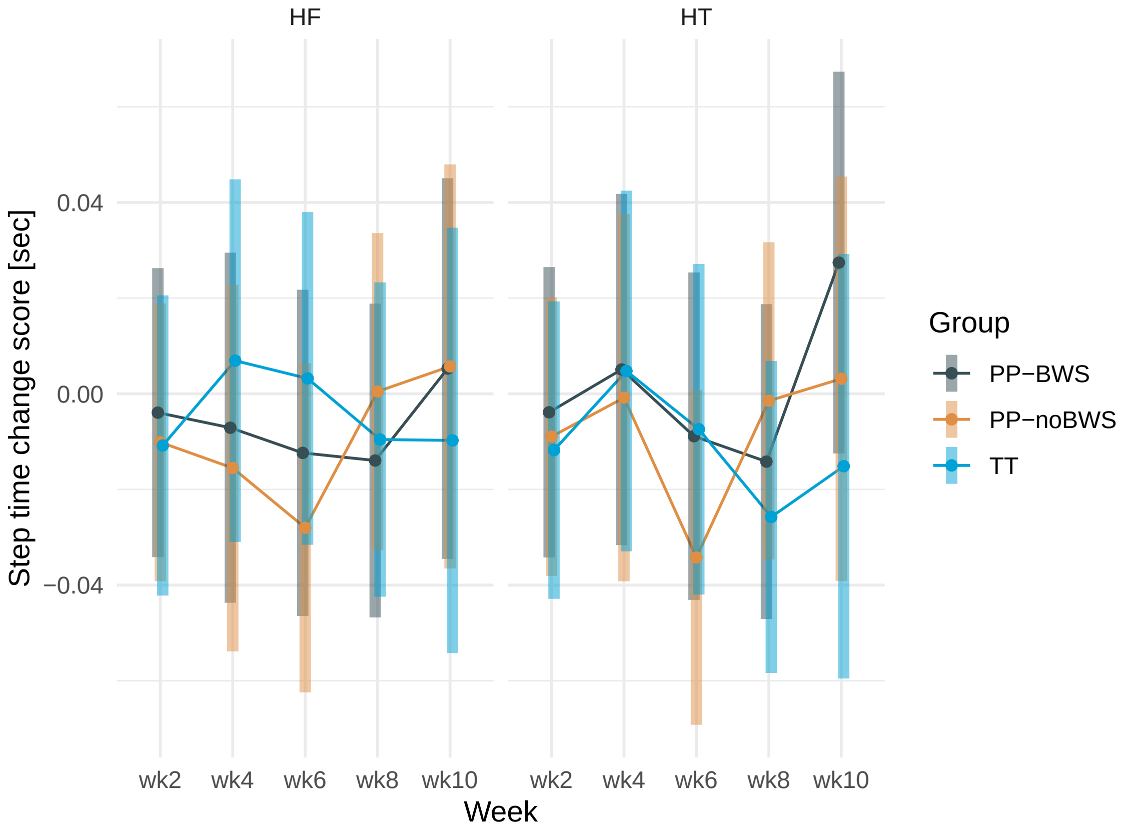

3 Sensitivity Analysis by Multiple-imputations

3.1 Postural Stability (PS)

3.1.1 Between Group Difference in Change Score

```
## Registered S3 method overwritten by 'rchiro':
##   method             from
##   is.nan.data.frame mice
```

Contrast	Task	Qual	Week	Difference±SE [95% CI], SMD	t[df], p-value
(PP-BWS) - (PP-noBWS)	Compliant	EC	wk2	0±0.1 [-0.3, 0.3], -0.01	t[121.5]=-0.072, 0.943
(PP-BWS) - TT	Compliant	EC	wk2	-0.2±0.1 [-0.5, 0.1], -0.14	t[110.4]=-1.482, 0.223
(PP-noBWS) - TT	Compliant	EC	wk2	-0.2±0.1 [-0.4, 0.1], -0.14	t[109.6]=-1.454, 0.223
(PP-BWS) - (PP-noBWS)	Firm	EC	wk2	0±0.1 [-0.3, 0.3], 0	t[109.4]=0.014, 0.989
(PP-BWS) - TT	Firm	EC	wk2	0±0.1 [-0.3, 0.2], -0.04	t[107.3]=-0.366, 0.989
(PP-noBWS) - TT	Firm	EC	wk2	0±0.1 [-0.3, 0.2], -0.04	t[101.7]=-0.384, 0.989
(PP-BWS) - (PP-noBWS)	Compliant	EO	wk2	0±0.1 [-0.3, 0.2], -0.04	t[121.5]=-0.418, 0.731
(PP-BWS) - TT	Compliant	EO	wk2	-0.1±0.1 [-0.4, 0.2], -0.07	t[107.6]=-0.724, 0.731
(PP-noBWS) - TT	Compliant	EO	wk2	0±0.1 [-0.3, 0.2], -0.03	t[107.2]=-0.345, 0.731
(PP-BWS) - (PP-noBWS)	Firm	EO	wk2	0.1±0.1 [-0.2, 0.3], 0.06	t[121.6]=0.712, 0.478
(PP-BWS) - TT	Firm	EO	wk2	-0.1±0.1 [-0.5, 0.2], -0.11	t[74.9]=-0.918, 0.478
(PP-noBWS) - TT	Firm	EO	wk2	-0.2±0.1 [-0.5, 0.1], -0.18	t[74.2]=-1.544, 0.381
(PP-BWS) - (PP-noBWS)	Compliant	EC	wk4	0±0.1 [-0.4, 0.3], -0.04	t[54.2]=-0.276, 0.784
(PP-BWS) - TT	Compliant	EC	wk4	-0.1±0.1 [-0.4, 0.2], -0.1	t[101.1]=-1.023, 0.784
(PP-noBWS) - TT	Compliant	EC	wk4	-0.1±0.1 [-0.4, 0.2], -0.08	t[61.8]=-0.602, 0.784
(PP-BWS) - (PP-noBWS)	Firm	EC	wk4	0±0.1 [-0.3, 0.4], 0.02	t[53.6]=0.165, 0.869
(PP-BWS) - TT	Firm	EC	wk4	0.1±0.1 [-0.2, 0.3], 0.05	t[97.6]=0.463, 0.869
(PP-noBWS) - TT	Firm	EC	wk4	0±0.1 [-0.3, 0.4], 0.03	t[60.6]=0.229, 0.869
(PP-BWS) - (PP-noBWS)	Compliant	EO	wk4	0.1±0.1 [-0.3, 0.4], 0.05	t[56.4]=0.369, 0.713
(PP-BWS) - TT	Compliant	EO	wk4	-0.1±0.1 [-0.3, 0.2], -0.05	t[98.5]=-0.451, 0.713
(PP-noBWS) - TT	Compliant	EO	wk4	-0.1±0.1 [-0.4, 0.2], -0.1	t[64.6]=-0.794, 0.713
(PP-BWS) - (PP-noBWS)	Firm	EO	wk4	-0.3±0.1 [-0.6, 0.1], -0.27	t[54.4]=-1.968, 0.081
(PP-BWS) - TT	Firm	EO	wk4	-0.3±0.1 [-0.6, 0], -0.29	t[97.8]=-2.869, 0.015
(PP-noBWS) - TT	Firm	EO	wk4	-0.1±0.1 [-0.4, 0.3], -0.06	t[61.9]=-0.45, 0.654
(PP-BWS) - (PP-noBWS)	Compliant	EC	wk6	0±0.1 [-0.3, 0.3], -0.02	t[58.6]=-0.139, 0.89
(PP-BWS) - TT	Compliant	EC	wk6	-0.1±0.1 [-0.3, 0.2], -0.07	t[117.9]=-0.71, 0.89
(PP-noBWS) - TT	Compliant	EC	wk6	-0.1±0.1 [-0.4, 0.3], -0.06	t[54.3]=-0.422, 0.89
(PP-BWS) - (PP-noBWS)	Firm	EC	wk6	0.1±0.1 [-0.2, 0.5], 0.13	t[59.4]=0.986, 0.687
(PP-BWS) - TT	Firm	EC	wk6	0.1±0.1 [-0.2, 0.3], 0.07	t[117.8]=0.713, 0.687
(PP-noBWS) - TT	Firm	EC	wk6	-0.1±0.1 [-0.4, 0.3], -0.05	t[55.2]=-0.405, 0.687

Contrast	Task	Qual	Week	Difference±SE [95% CI], SMD	t[df], p-value
(PP-BWS) - (PP-noBWS)	Compliant	EO	wk6	0±0.1 [-0.3, 0.3], 0.01	t[55.4]=0.04, 0.968
(PP-BWS) - TT	Compliant	EO	wk6	-0.1±0.1 [-0.3, 0.2], -0.07	t[117.8]=-0.793, 0.777
(PP-noBWS) - TT	Compliant	EO	wk6	-0.1±0.1 [-0.4, 0.2], -0.09	t[51.7]=-0.651, 0.777
(PP-BWS) - (PP-noBWS)	Firm	EO	wk6	0±0.1 [-0.4, 0.3], -0.05	t[58.8]=-0.363, 0.718
(PP-BWS) - TT	Firm	EO	wk6	-0.2±0.1 [-0.4, 0.1], -0.17	t[117.9]=-1.847, 0.202
(PP-noBWS) - TT	Firm	EO	wk6	-0.1±0.1 [-0.5, 0.2], -0.15	t[54.6]=-1.097, 0.416
(PP-BWS) - (PP-noBWS)	Compliant	EC	wk8	0.2±0.1 [-0.1, 0.5], 0.18	t[69.8]=1.47, 0.297
(PP-BWS) - TT	Compliant	EC	wk8	0±0.1 [-0.3, 0.3], 0.01	t[94.3]=0.061, 0.952
(PP-noBWS) - TT	Compliant	EC	wk8	-0.2±0.1 [-0.6, 0.2], -0.18	t[54.2]=-1.303, 0.297
(PP-BWS) - (PP-noBWS)	Firm	EC	wk8	0.2±0.1 [-0.1, 0.6], 0.22	t[71.8]=1.839, 0.131
(PP-BWS) - TT	Firm	EC	wk8	0±0.1 [-0.3, 0.3], 0	t[95.1]=-0.044, 0.965
(PP-noBWS) - TT	Firm	EC	wk8	-0.3±0.1 [-0.6, 0.1], -0.23	t[56.3]=-1.74, 0.131
(PP-BWS) - (PP-noBWS)	Compliant	EO	wk8	0.2±0.1 [-0.1, 0.6], 0.2	t[69.2]=1.633, 0.166
(PP-BWS) - TT	Compliant	EO	wk8	0±0.1 [-0.3, 0.3], -0.01	t[92.1]=-0.141, 0.889
(PP-noBWS) - TT	Compliant	EO	wk8	-0.2±0.1 [-0.6, 0.1], -0.22	t[53.4]=-1.622, 0.166
(PP-BWS) - (PP-noBWS)	Firm	EO	wk8	0.2±0.1 [-0.2, 0.5], 0.13	t[71.7]=1.116, 0.402
(PP-BWS) - TT	Firm	EO	wk8	-0.1±0.1 [-0.4, 0.2], -0.05	t[94.6]=-0.48, 0.632
(PP-noBWS) - TT	Firm	EO	wk8	-0.2±0.1 [-0.6, 0.2], -0.19	t[54.4]=-1.429, 0.402
(PP-BWS) - (PP-noBWS)	Compliant	EC	wk10	0.1±0.2 [-0.4, 0.6], 0.07	t[37.6]=0.41, 0.772
(PP-BWS) - TT	Compliant	EC	wk10	0.1±0.2 [-0.4, 0.6], 0.1	t[41.7]=0.676, 0.772
(PP-noBWS) - TT	Compliant	EC	wk10	0.1±0.2 [-0.4, 0.5], 0.04	t[52.6]=0.292, 0.772
(PP-BWS) - (PP-noBWS)	Firm	EC	wk10	0.1±0.2 [-0.4, 0.6], 0.07	t[35.6]=0.446, 0.893
(PP-BWS) - TT	Firm	EC	wk10	0.1±0.2 [-0.4, 0.6], 0.09	t[41]=0.583, 0.893
(PP-noBWS) - TT	Firm	EC	wk10	0±0.2 [-0.4, 0.5], 0.02	t[48.3]=0.135, 0.893
(PP-BWS) - (PP-noBWS)	Compliant	EO	wk10	0.1±0.2 [-0.4, 0.6], 0.06	t[38.1]=0.376, 0.986
(PP-BWS) - TT	Compliant	EO	wk10	0.1±0.2 [-0.4, 0.6], 0.06	t[38.7]=0.385, 0.986
(PP-noBWS) - TT	Compliant	EO	wk10	0±0.2 [-0.4, 0.4], 0	t[54.2]=0.018, 0.986
(PP-BWS) - (PP-noBWS)	Firm	EO	wk10	0.1±0.2 [-0.4, 0.6], 0.06	t[36.5]=0.383, 0.79
(PP-BWS) - TT	Firm	EO	wk10	0.1±0.2 [-0.4, 0.6], 0.1	t[41.6]=0.633, 0.79
(PP-noBWS) - TT	Firm	EO	wk10	0±0.2 [-0.4, 0.5], 0.04	t[51.5]=0.268, 0.79
