# Supplementary material for: Comparative effectiveness of lower body positive pressure and traditional treadmill training on adults with mild balance impairment
Source: Front Aging. 2025 Oct 22;6:1645026. doi: 10.3389/fragi.2025.1645026 (PMC12586058; doi:10.3389/fragi.2025.1645026)
Supplement: Supplementary file 3 [file DataSheet3.pdf]

AGT RCT FP Analysis

Usman Rashid

13/09/2025

Contents

1 Statistical Analysis 1

2 Results 1
- 2.1 Total Distance Wandered . . . 1
  - 2.1.1 Model Diagnostics . . . 2
  - 2.1.2 Anova . . . 2
  - 2.1.3 Between Group Differences . . . 3
  - 2.1.4 Change over Time . . . 5
- 2.2 Total ML Sway . . . 6
  - 2.2.1 Model Diagnostics . . . 6
  - 2.2.2 Anova . . . 6
  - 2.2.3 Between Group Differences . . . 7
  - 2.2.4 Change over Time . . . 9
- 2.3 Total AP Sway . . . 10
  - 2.3.1 Model Diagnostics . . . 10
  - 2.3.2 Anova . . . 10
  - 2.3.3 Change over Time . . . 11

3 Sensitivity Analysis by Multiple-imputations 12
- 3.1 Total Distance Wandered . . . 12
  - 3.1.1 Between Group Difference in Change Score . . . 12
- 3.2 Total ML Sway . . . 14
  - 3.2.1 Between Group Difference in Change Score . . . 14

1 Statistical Analysis

Statistical models are fitted to change scores (Post- - Pre-intervention values). Thus, a positive score implies that the post-intervention value is larger than the pre-intervention value. Benjamini-Hochberg adjustment is applied for between group differences.

2 Results

2.1 Total Distance Wandered

```
## boundary (singular) fit: see help('isSingular')
```

```
## [1] 5949.549
```

2.1.1 Model Diagnostics

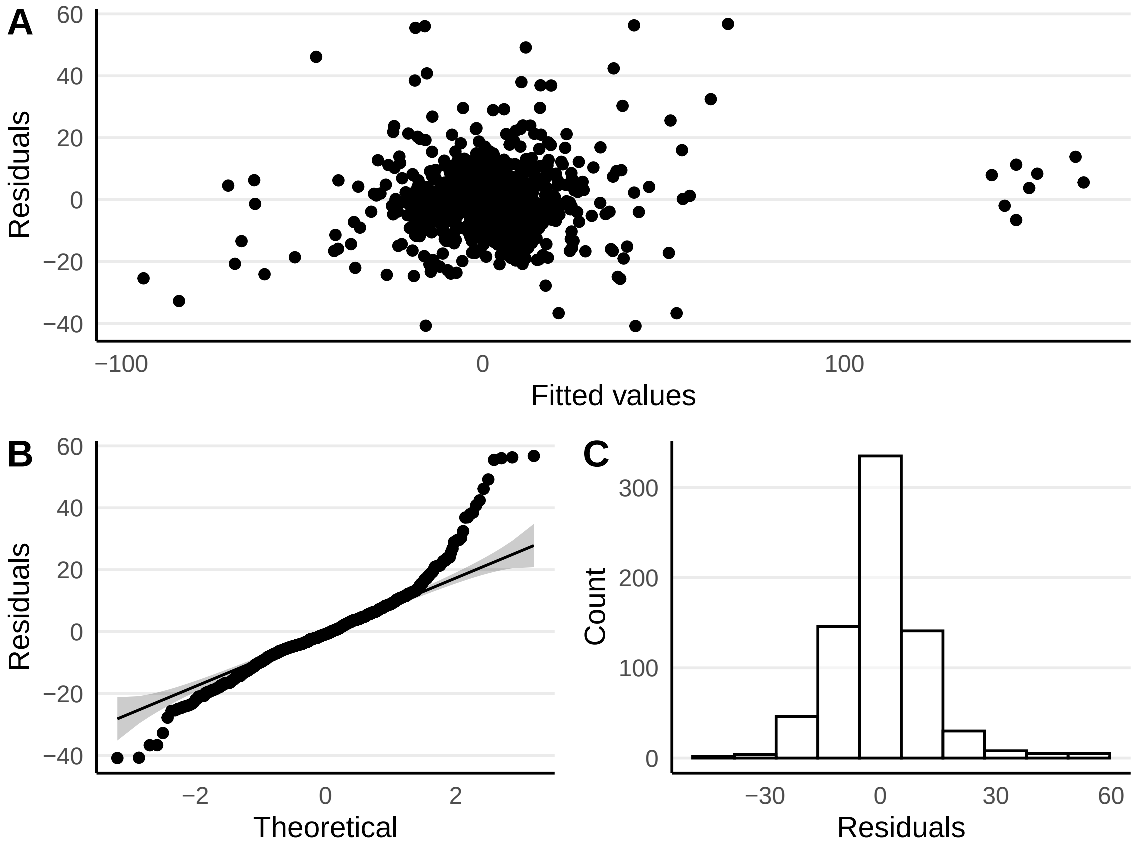

2.1.2 Anova

```
Analysis of Deviance Table (Type III Wald chisquare tests)

Response: (Value - Value.pre)
                       Chisq Df Pr(>Chisq)
(Intercept)           0.5615  1   0.453639
Value.pre           338.5363  1  < 2.2e-16 ***
Age_years             1.5644  1   0.211027
Height_cm             0.0266  1   0.870488
BMI                   0.0699  1   0.791503
Task                  0.0669  1   0.795936
Qual                  2.2846  1   0.130661
Group                 2.8195  2   0.244207
Week                  2.4897  4   0.646487
Task:Qual             0.0444  1   0.833074
Task:Group            9.8462  2   0.007277 **
Qual:Group            5.4940  2   0.064119 .
Task:Week             8.6300  4   0.071044 .
Qual:Week             1.9698  4   0.741309
```

```
Group:Week             25.0784  8   0.001508 **
Task:Qual:Group         0.7131  2   0.700075
Task:Qual:Week          1.7142  4   0.788139
Task:Group:Week        22.9174  8   0.003472 **
Qual:Group:Week        10.9158  8   0.206514
Task:Qual:Group:Week    4.4270  8   0.816688
---
Signif. codes:  0 '***' 0.001 '**' 0.01 '*' 0.05 '.' 0.1 ' ' 1
```

2.1.3 Between Group Differences

Contrast	Task	Qual	Week	Difference±SE [95% CI], SMD	t[df], p-value
(PP-BWS) - (PP-noBWS)	Compliant	EC	2	20±10 [-20, 50], 0.2	t[51.5]=1.213, 0.346
(PP-BWS) - TT	Compliant	EC	2	-10±10 [-40, 30], -0.1	t[51.8]=-0.405, 0.687
(PP-noBWS) - TT	Compliant	EC	2	-20±10 [-60, 10], -0.2	t[52.3]=-1.587, 0.346
(PP-BWS) - (PP-noBWS)	Firm	EC	2	20±10 [-10, 60], 0.2	t[52.1]=1.656, 0.311
(PP-BWS) - TT	Firm	EC	2	20±10 [-20, 50], 0.2	t[51.1]=1.204, 0.351
(PP-noBWS) - TT	Firm	EC	2	-10±10 [-40, 30], -0.1	t[52.4]=-0.423, 0.674
(PP-BWS) - (PP-noBWS)	Compliant	EO	2	20±10 [-20, 50], 0.2	t[51.4]=1.151, 0.629
(PP-BWS) - TT	Compliant	EO	2	10±10 [-30, 40], 0.1	t[50.7]=0.643, 0.629
(PP-noBWS) - TT	Compliant	EO	2	-10±10 [-40, 30], -0.1	t[51.2]=-0.487, 0.629
(PP-BWS) - (PP-noBWS)	Firm	EO	2	20±10 [-10, 60], 0.2	t[51.9]=1.599, 0.174
(PP-BWS) - TT	Firm	EO	2	20±10 [-10, 60], 0.2	t[52.4]=1.689, 0.174
(PP-noBWS) - TT	Firm	EO	2	0±10 [-30, 40], 0	t[52.2]=0.128, 0.899
(PP-BWS) - (PP-noBWS)	Compliant	EC	4	11±7 [-6, 28], 0.1	t[150.7]=1.577, 0.175
(PP-BWS) - TT	Compliant	EC	4	-5±6 [-21, 10], -0.1	t[141.9]=-0.842, 0.401
(PP-noBWS) - TT	Compliant	EC	4	-16±7 [-33, 0], -0.2	t[150.7]=-2.421, 0.05
(PP-BWS) - (PP-noBWS)	Firm	EC	4	3±7 [-14, 20], 0	t[144.7]=0.43, 0.668
(PP-BWS) - TT	Firm	EC	4	-6±6 [-21, 9], -0.1	t[131.2]=-0.966, 0.504
(PP-noBWS) - TT	Firm	EC	4	-9±7 [-25, 7], -0.1	t[146.9]=-1.341, 0.504
(PP-BWS) - (PP-noBWS)	Compliant	EO	4	6±7 [-11, 23], 0.1	t[144.8]=0.862, 0.39
(PP-BWS) - TT	Compliant	EO	4	-9±6 [-24, 6], -0.1	t[127.1]=-1.488, 0.209
(PP-noBWS) - TT	Compliant	EO	4	-15±7 [-31, 1], -0.2	t[143.5]=-2.274, 0.073
(PP-BWS) - (PP-noBWS)	Firm	EO	4	0±7 [-17, 17], 0	t[144.9]=-0.007, 0.995
(PP-BWS) - TT	Firm	EO	4	-7±6 [-23, 8], -0.1	t[131.4]=-1.199, 0.405
(PP-noBWS) - TT	Firm	EO	4	-7±7 [-24, 9], -0.1	t[147.5]=-1.108, 0.405
(PP-BWS) - (PP-noBWS)	Compliant	EC	6	-20±8 [-40, 0], -0.3	t[80.9]=-2.41, 0.055
(PP-BWS) - TT	Compliant	EC	6	-8±9 [-28, 13], -0.1	t[87.2]=-0.887, 0.377
(PP-noBWS) - TT	Compliant	EC	6	13±9 [-9, 34], 0.2	t[85.7]=1.426, 0.236
(PP-BWS) - (PP-noBWS)	Firm	EC	6	-1±8 [-21, 20], 0	t[81]=-0.095, 0.925
(PP-BWS) - TT	Firm	EC	6	-6±8 [-26, 14], -0.1	t[79.3]=-0.723, 0.829

Contrast	Task	Qual	Week	Difference±SE [95% CI], SMD	t[df], p-value
(PP-noBWS) - TT	Firm	EC	6	-5±9 [-27, 16], -0.1	t[85.3]=-0.596, 0.829
(PP-BWS) - (PP-noBWS)	Compliant	EO	6	-9±8 [-29, 12], -0.1	t[85.8]=-1.011, 0.648
(PP-BWS) - TT	Compliant	EO	6	-4±8 [-24, 17], -0.1	t[81.4]=-0.458, 0.648
(PP-noBWS) - TT	Compliant	EO	6	5±9 [-17, 26], 0.1	t[83.1]=0.541, 0.648
(PP-BWS) - (PP-noBWS)	Firm	EO	6	-2±8 [-22, 19], 0	t[81]=-0.201, 0.962
(PP-BWS) - TT	Firm	EO	6	-2±8 [-23, 18], 0	t[83]=-0.249, 0.962
(PP-noBWS) - TT	Firm	EO	6	0±9 [-22, 21], 0	t[88.8]=-0.048, 0.962
(PP-BWS) - (PP-noBWS)	Compliant	EC	8	-3±6 [-18, 12], 0	t[181.1]=-0.478, 0.633
(PP-BWS) - TT	Compliant	EC	8	10±6 [-4, 24], 0.1	t[189.7]=1.695, 0.138
(PP-noBWS) - TT	Compliant	EC	8	13±6 [-2, 27], 0.2	t[195.6]=2.137, 0.102
(PP-BWS) - (PP-noBWS)	Firm	EC	8	-13±6 [-28, 2], -0.2	t[187.1]=-2.146, 0.1
(PP-BWS) - TT	Firm	EC	8	-4±6 [-18, 10], 0	t[185.4]=-0.68, 0.497
(PP-noBWS) - TT	Firm	EC	8	9±6 [-6, 24], 0.1	t[203.8]=1.503, 0.202
(PP-BWS) - (PP-noBWS)	Compliant	EO	8	-6±6 [-21, 8], -0.1	t[180.6]=-1.061, 0.435
(PP-BWS) - TT	Compliant	EO	8	1±6 [-13, 14], 0	t[173.4]=0.094, 0.925
(PP-noBWS) - TT	Compliant	EO	8	7±6 [-7, 21], 0.1	t[182.1]=1.183, 0.435
(PP-BWS) - (PP-noBWS)	Firm	EO	8	-7±6 [-21, 8], -0.1	t[180.3]=-1.08, 0.427
(PP-BWS) - TT	Firm	EO	8	0±6 [-14, 14], 0	t[187]=-0.013, 0.99
(PP-noBWS) - TT	Firm	EO	8	6±6 [-8, 21], 0.1	t[195.6]=1.072, 0.427
(PP-BWS) - (PP-noBWS)	Compliant	EC	10	0±10 [-30, 40], 0.1	t[57.5]=0.392, 0.785
(PP-BWS) - TT	Compliant	EC	10	0±10 [-30, 30], 0	t[55.8]=-0.275, 0.785
(PP-noBWS) - TT	Compliant	EC	10	-10±10 [-40, 20], -0.1	t[55.5]=-0.68, 0.785
(PP-BWS) - (PP-noBWS)	Firm	EC	10	0±10 [-30, 30], 0	t[60.9]=-0.106, 0.916
(PP-BWS) - TT	Firm	EC	10	-10±10 [-30, 20], -0.1	t[55.6]=-0.493, 0.916
(PP-noBWS) - TT	Firm	EC	10	0±10 [-40, 30], 0	t[60.4]=-0.353, 0.916
(PP-BWS) - (PP-noBWS)	Compliant	EO	10	0±10 [-30, 30], 0	t[61.3]=-0.116, 0.908
(PP-BWS) - TT	Compliant	EO	10	0±10 [-30, 30], 0	t[55.6]=0.233, 0.908
(PP-noBWS) - TT	Compliant	EO	10	0±10 [-30, 30], 0	t[60.6]=0.338, 0.908
(PP-BWS) - (PP-noBWS)	Firm	EO	10	0±10 [-30, 30], 0	t[66.9]=0.033, 0.974
(PP-BWS) - TT	Firm	EO	10	10±10 [-20, 40], 0.1	t[62.9]=0.687, 0.789
(PP-noBWS) - TT	Firm	EO	10	10±10 [-20, 40], 0.1	t[60.4]=0.637, 0.789

2.1.4 Change over Time

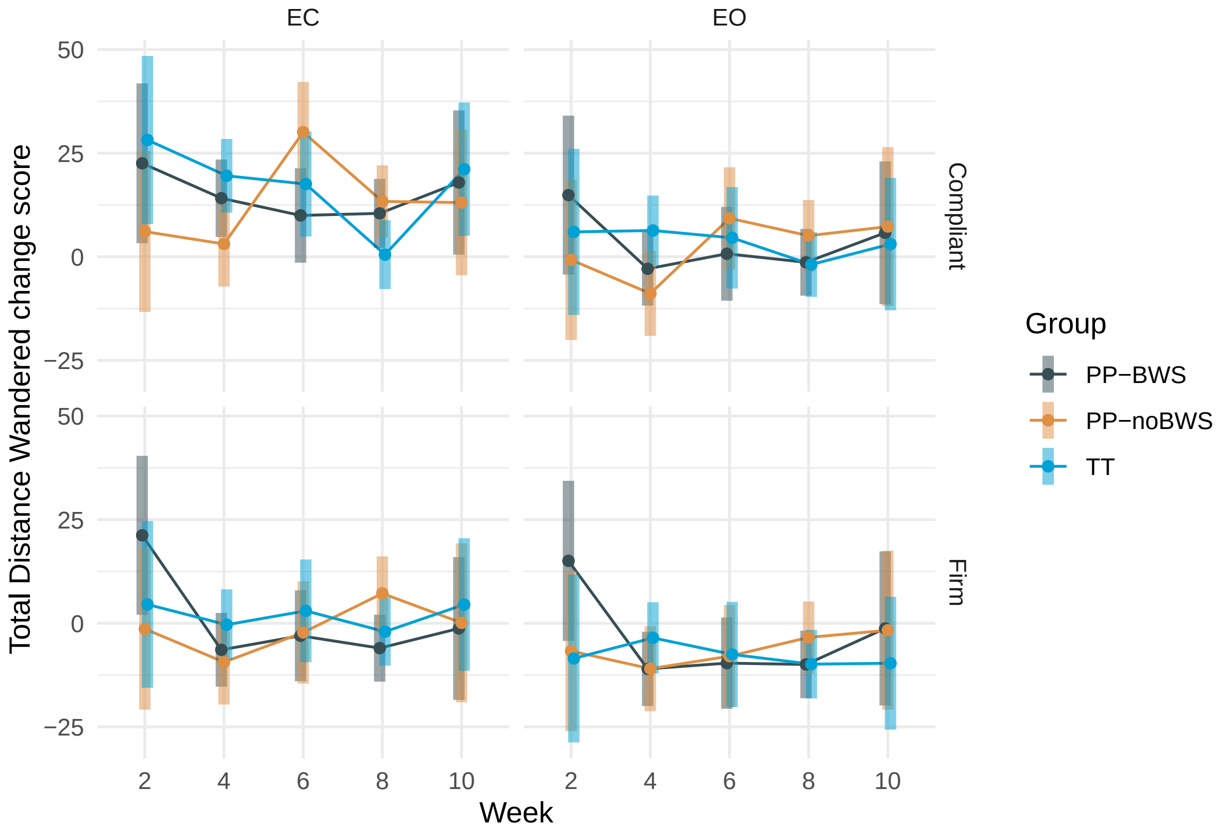

2.2 Total ML Sway

2.2.1 Model Diagnostics

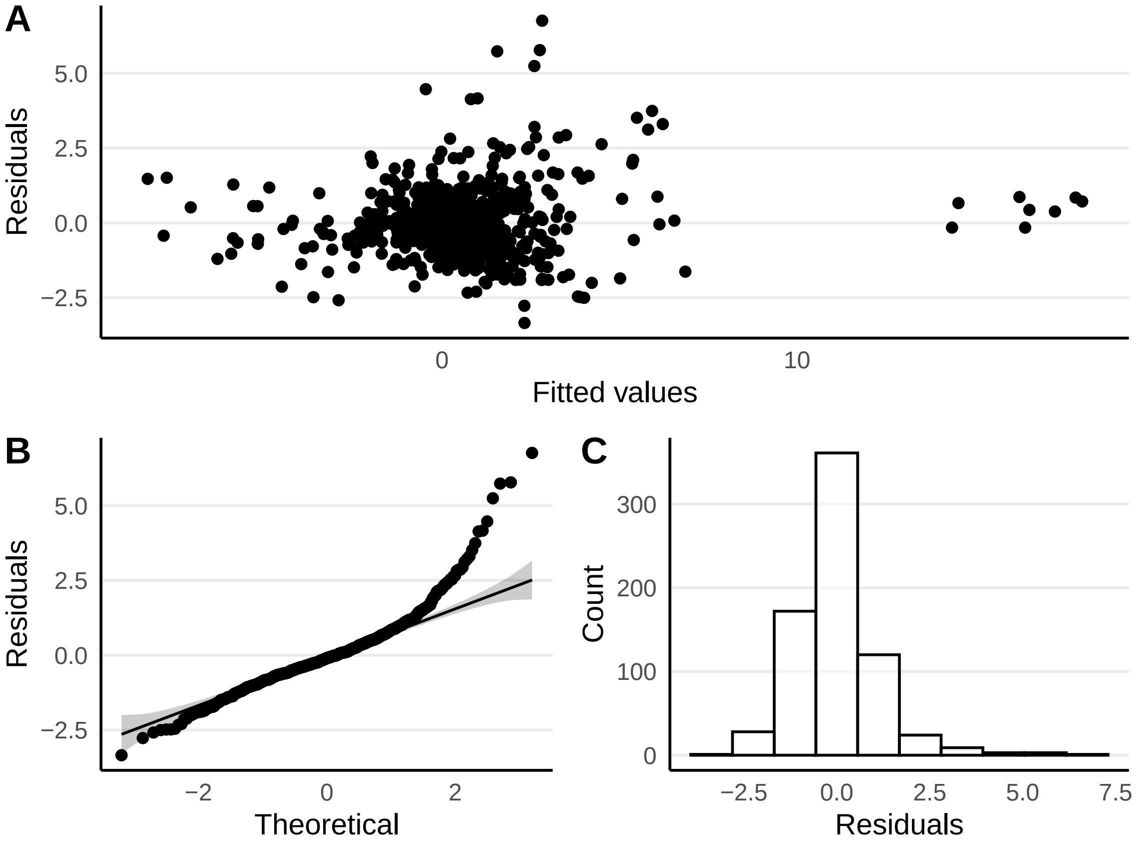

2.2.2 Anova

```
Analysis of Deviance Table (Type III Wald chisquare tests)

Response: (Value - Value.pre)
                        Chisq Df Pr(>Chisq)
(Intercept)            0.7821  1   0.376503
Value.pre            388.6382  1  < 2.2e-16 ***
Age_years              2.2711  1   0.131808
Height_cm              0.0034  1   0.953683
BMI                    0.0022  1   0.962670
Task                   2.7641  1   0.096401 .
Qual                   1.0920  1   0.296037
Group                  1.6771  2   0.432345
Week                  17.5257  4   0.001527 **
Task:Qual              0.2868  1   0.592273
Task:Group             0.7868  2   0.674749
Qual:Group             0.8257  2   0.661771
Task:Week              2.6233  4   0.622696
Qual:Week              3.1459  4   0.533719
Group:Week            39.2195  8  4.475e-06 ***
```

```
Task:Qual:Group        1.9775  2   0.372035
Task:Qual:Week         2.5588  4   0.634133
Task:Group:Week        5.7241  8   0.678101
Qual:Group:Week        7.6388  8   0.469521
Task:Qual:Group:Week   4.1128  8   0.846806
---
Signif. codes:  0 '***' 0.001 '**' 0.01 '*' 0.05 '.' 0.1 ' ' 1
```

2.2.3 Between Group Differences

Contrast	Task	Qual	Week	Difference±SE [95% CI], SMD	t[df], p-value
(PP-BWS) - (PP-noBWS)	Compliant	EC	2	2±1 [-2, 6], 0.2	t[48.7]=1.288, 0.552
(PP-BWS) - TT	Compliant	EC	2	1±1 [-3, 5], 0.1	t[49.5]=0.664, 0.552
(PP-noBWS) - TT	Compliant	EC	2	-1±1 [-5, 3], -0.1	t[49.9]=-0.599, 0.552
(PP-BWS) - (PP-noBWS)	Firm	EC	2	2±1 [-1, 6], 0.2	t[49.3]=1.684, 0.296
(PP-BWS) - TT	Firm	EC	2	1±1 [-2, 5], 0.1	t[49]=0.932, 0.472
(PP-noBWS) - TT	Firm	EC	2	-1±1 [-5, 3], -0.1	t[49.9]=-0.725, 0.472
(PP-BWS) - (PP-noBWS)	Compliant	EO	2	2±1 [-1, 6], 0.2	t[48.8]=1.701, 0.286
(PP-BWS) - TT	Compliant	EO	2	1±1 [-2, 5], 0.1	t[48.7]=0.899, 0.442
(PP-noBWS) - TT	Compliant	EO	2	-1±1 [-5, 3], -0.1	t[49.2]=-0.775, 0.442
(PP-BWS) - (PP-noBWS)	Firm	EO	2	2±1 [-2, 5], 0.2	t[49]=1.251, 0.434
(PP-BWS) - TT	Firm	EO	2	2±1 [-2, 5], 0.2	t[50]=1.071, 0.434
(PP-noBWS) - TT	Firm	EO	2	0±1 [-4, 3], 0	t[49.8]=-0.155, 0.877
(PP-BWS) - (PP-noBWS)	Compliant	EC	4	-0.1±0.7 [-1.8, 1.6], 0	t[121.3]=-0.158, 0.875
(PP-BWS) - TT	Compliant	EC	4	-1±0.6 [-2.5, 0.6], -0.1	t[115.3]=-1.485, 0.328
(PP-noBWS) - TT	Compliant	EC	4	-0.8±0.7 [-2.5, 0.8], -0.1	t[123.4]=-1.237, 0.328
(PP-BWS) - (PP-noBWS)	Firm	EC	4	-0.6±0.7 [-2.3, 1.1], -0.1	t[116.7]=-0.853, 0.395
(PP-BWS) - TT	Firm	EC	4	-1.7±0.6 [-3.2, -0.1], -0.3	t[107.1]=-2.653, 0.028*
(PP-noBWS) - TT	Firm	EC	4	-1.1±0.7 [-2.7, 0.6], -0.1	t[118.8]=-1.598, 0.169
(PP-BWS) - (PP-noBWS)	Compliant	EO	4	-0.1±0.7 [-1.8, 1.5], 0	t[117.1]=-0.207, 0.837
(PP-BWS) - TT	Compliant	EO	4	-1.1±0.6 [-2.6, 0.5], -0.2	t[104.3]=-1.698, 0.262
(PP-noBWS) - TT	Compliant	EO	4	-0.9±0.7 [-2.6, 0.7], -0.1	t[117.1]=-1.366, 0.262
(PP-BWS) - (PP-noBWS)	Firm	EO	4	-0.8±0.7 [-2.5, 0.9], -0.1	t[116.6]=-1.155, 0.25
(PP-BWS) - TT	Firm	EO	4	-1.7±0.6 [-3.2, -0.1], -0.3	t[107]=-2.63, 0.029*
(PP-noBWS) - TT	Firm	EO	4	-0.9±0.7 [-2.5, 0.8], -0.1	t[118.9]=-1.263, 0.25
(PP-BWS) - (PP-noBWS)	Compliant	EC	6	-2±0.8 [-3.9, -0.1], -0.3	t[78.2]=-2.535, 0.04*
(PP-BWS) - TT	Compliant	EC	6	-1±0.8 [-3, 0.9], -0.1	t[80.3]=-1.285, 0.257
(PP-noBWS) - TT	Compliant	EC	6	0.9±0.8 [-1.1, 3], 0.1	t[79.4]=1.141, 0.257
(PP-BWS) - (PP-noBWS)	Firm	EC	6	-1.4±0.8 [-3.3, 0.5], -0.2	t[78]=-1.822, 0.164
(PP-BWS) - TT	Firm	EC	6	-1.3±0.8 [-3.2, 0.6], -0.2	t[73.9]=-1.62, 0.164
(PP-noBWS) - TT	Firm	EC	6	0.2±0.8 [-1.9, 2.2], 0	t[78.3]=0.185, 0.854

Contrast	Task	Qual	Week	Difference±SE [95% CI], SMD	t[df], p-value
(PP-BWS) - (PP-noBWS)	Compliant	EO	6	-0.6±0.8 [-2.6, 1.3], -0.1	t[83.5]=-0.773, 0.733
(PP-BWS) - TT	Compliant	EO	6	-0.3±0.8 [-2.2, 1.7], 0	t[75.8]=-0.342, 0.733
(PP-noBWS) - TT	Compliant	EO	6	0.3±0.8 [-1.7, 2.4], 0	t[77.3]=0.417, 0.733
(PP-BWS) - (PP-noBWS)	Firm	EO	6	-1.2±0.8 [-3.1, 0.8], -0.2	t[78.1]=-1.481, 0.214
(PP-BWS) - TT	Firm	EO	6	-1.2±0.8 [-3.2, 0.7], -0.2	t[77.4]=-1.537, 0.214
(PP-noBWS) - TT	Firm	EO	6	-0.1±0.8 [-2.1, 2], 0	t[81.7]=-0.075, 0.941
(PP-BWS) - (PP-noBWS)	Compliant	EC	8	-2.1±0.7 [-3.9, -0.4], -0.3	t[94.2]=-2.948, 0.006*
(PP-BWS) - TT	Compliant	EC	8	0.5±0.7 [-1.2, 2.2], 0.1	t[95.2]=0.768, 0.445
(PP-noBWS) - TT	Compliant	EC	8	2.7±0.7 [0.9, 4.4], 0.4	t[98]=3.715, 0.001*
(PP-BWS) - (PP-noBWS)	Firm	EC	8	-2±0.7 [-3.8, -0.2], -0.3	t[97.6]=-2.733, 0.022*
(PP-BWS) - TT	Firm	EC	8	-0.9±0.7 [-2.6, 0.8], -0.1	t[94.5]=-1.343, 0.182
(PP-noBWS) - TT	Firm	EC	8	1.1±0.7 [-0.7, 2.8], 0.1	t[101.2]=1.461, 0.182
(PP-BWS) - (PP-noBWS)	Compliant	EO	8	-1.6±0.7 [-3.4, 0.2], -0.2	t[94.7]=-2.218, 0.087
(PP-BWS) - TT	Compliant	EO	8	-0.4±0.7 [-2, 1.3], -0.1	t[89.5]=-0.521, 0.604
(PP-noBWS) - TT	Compliant	EO	8	1.3±0.7 [-0.5, 3], 0.2	t[92.6]=1.764, 0.122
(PP-BWS) - (PP-noBWS)	Firm	EO	8	-1.1±0.7 [-2.9, 0.6], -0.2	t[94]=-1.551, 0.373
(PP-BWS) - TT	Firm	EO	8	-0.8±0.7 [-2.5, 0.9], -0.1	t[94.8]=-1.1, 0.411
(PP-noBWS) - TT	Firm	EO	8	0.4±0.7 [-1.4, 2.1], 0	t[96.9]=0.492, 0.624
(PP-BWS) - (PP-noBWS)	Compliant	EC	10	2.2±0.9 [0, 4.4], 0.2	t[127.3]=2.414, 0.026*
(PP-BWS) - TT	Compliant	EC	10	2.3±0.9 [0.2, 4.4], 0.2	t[126.7]=2.634, 0.026*
(PP-noBWS) - TT	Compliant	EC	10	0.1±0.9 [-2, 2.2], 0	t[125.3]=0.096, 0.924
(PP-BWS) - (PP-noBWS)	Firm	EC	10	1.9±0.9 [-0.4, 4.2], 0.2	t[125.3]=2.006, 0.141
(PP-BWS) - TT	Firm	EC	10	1.4±0.9 [-0.7, 3.5], 0.1	t[126.1]=1.605, 0.166
(PP-noBWS) - TT	Firm	EC	10	-0.5±0.9 [-2.7, 1.7], -0.1	t[128.6]=-0.569, 0.57
(PP-BWS) - (PP-noBWS)	Compliant	EO	10	1.1±0.9 [-1.2, 3.3], 0.1	t[126.6]=1.109, 0.617
(PP-BWS) - TT	Compliant	EO	10	0.7±0.9 [-1.4, 2.8], 0.1	t[126.5]=0.824, 0.617
(PP-noBWS) - TT	Compliant	EO	10	-0.3±0.9 [-2.5, 1.9], 0	t[128.5]=-0.377, 0.707
(PP-BWS) - (PP-noBWS)	Firm	EO	10	2.2±1 [-0.2, 4.6], 0.2	t[121.9]=2.264, 0.076
(PP-BWS) - TT	Firm	EO	10	0.5±0.9 [-1.7, 2.7], 0	t[129.4]=0.535, 0.594
(PP-noBWS) - TT	Firm	EO	10	-1.8±0.9 [-4, 0.4], -0.2	t[127.6]=-1.933, 0.083

2.2.4 Change over Time

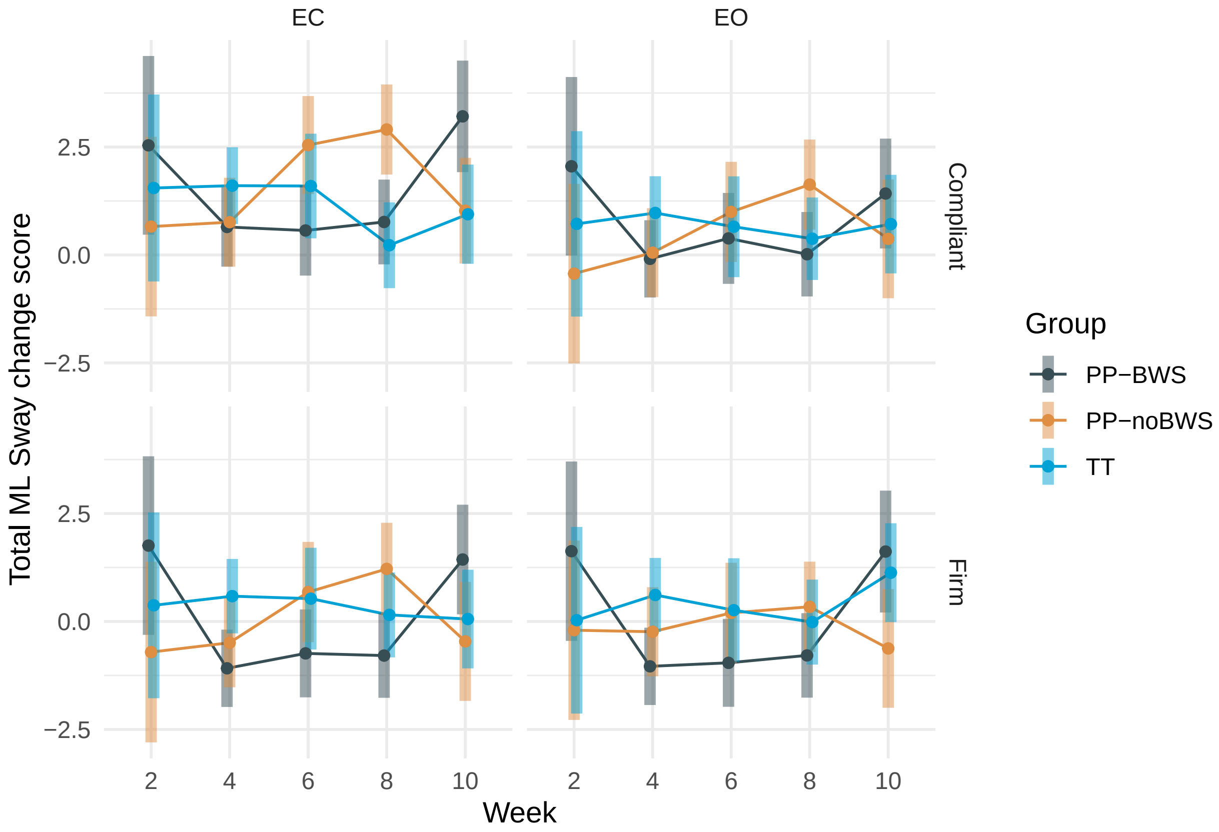

2.3 Total AP Sway

```
## boundary (singular) fit: see help('isSingular')
```

2.3.1 Model Diagnostics

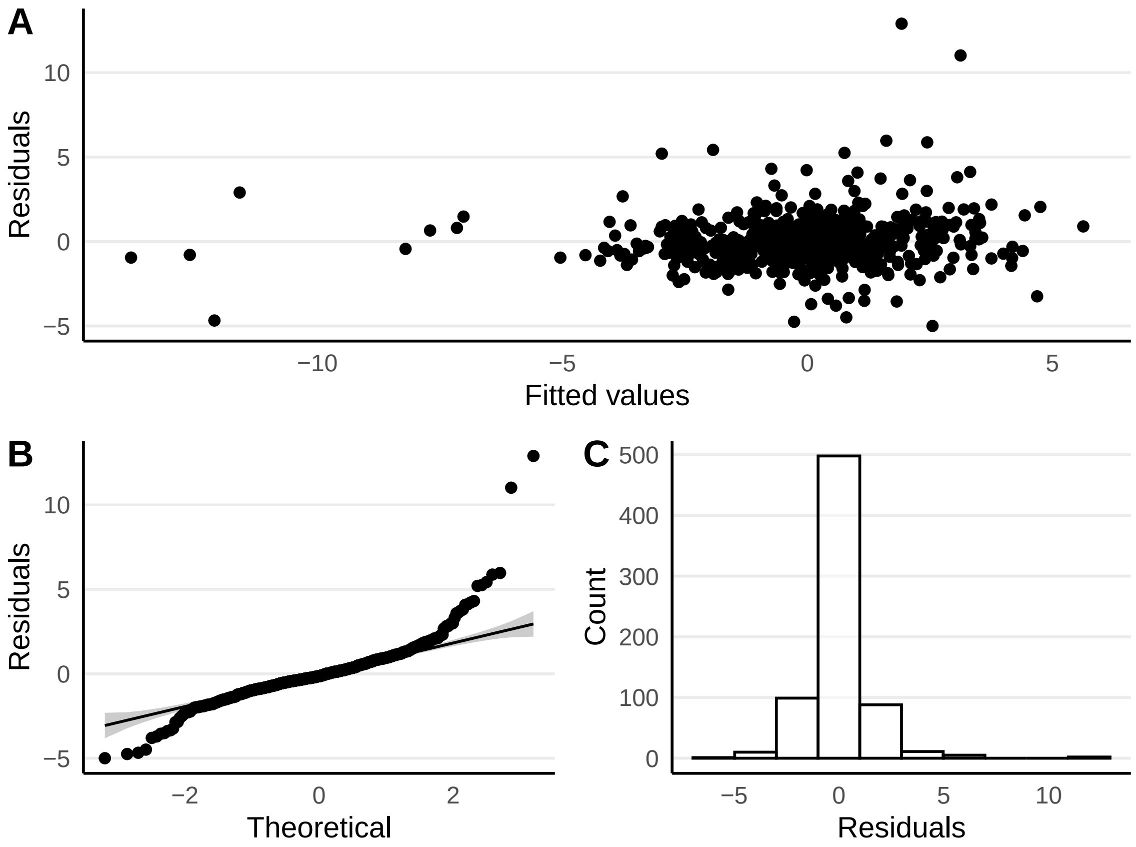

2.3.2 Anova

```
Analysis of Deviance Table (Type III Wald chisquare tests)

Response: (Value - Value.pre)
                        Chisq Df Pr(>Chisq)
(Intercept)            0.6671  1    0.41408
Value.pre            508.8494  1    < 2e-16 ***
Age_years              1.2222  1    0.26893
Height_cm              1.4302  1    0.23174
BMI                    2.1283  1    0.14460
Task                   1.1000  1    0.29427
Qual                   2.2320  1    0.13518
Group                  1.5541  2    0.45976
Week                   0.6609  4    0.95606
Task:Qual              0.1890  1    0.66372
Task:Group             2.5111  2    0.28493
Qual:Group             0.7726  2    0.67957
```

```
Task:Week              5.4251  4    0.24639
Qual:Week              0.5769  4    0.96559
Group:Week             2.8560  8    0.94306
Task:Qual:Group        1.1802  2    0.55429
Task:Qual:Week         0.3496  4    0.98639
Task:Group:Week       13.4900  8    0.09607 .
Qual:Group:Week        2.5495  8    0.95938
Task:Qual:Group:Week   6.1650  8    0.62876
---
Signif. codes:  0 '***' 0.001 '**' 0.01 '*' 0.05 '.' 0.1 ' ' 1
```

2.3.3 Change over Time

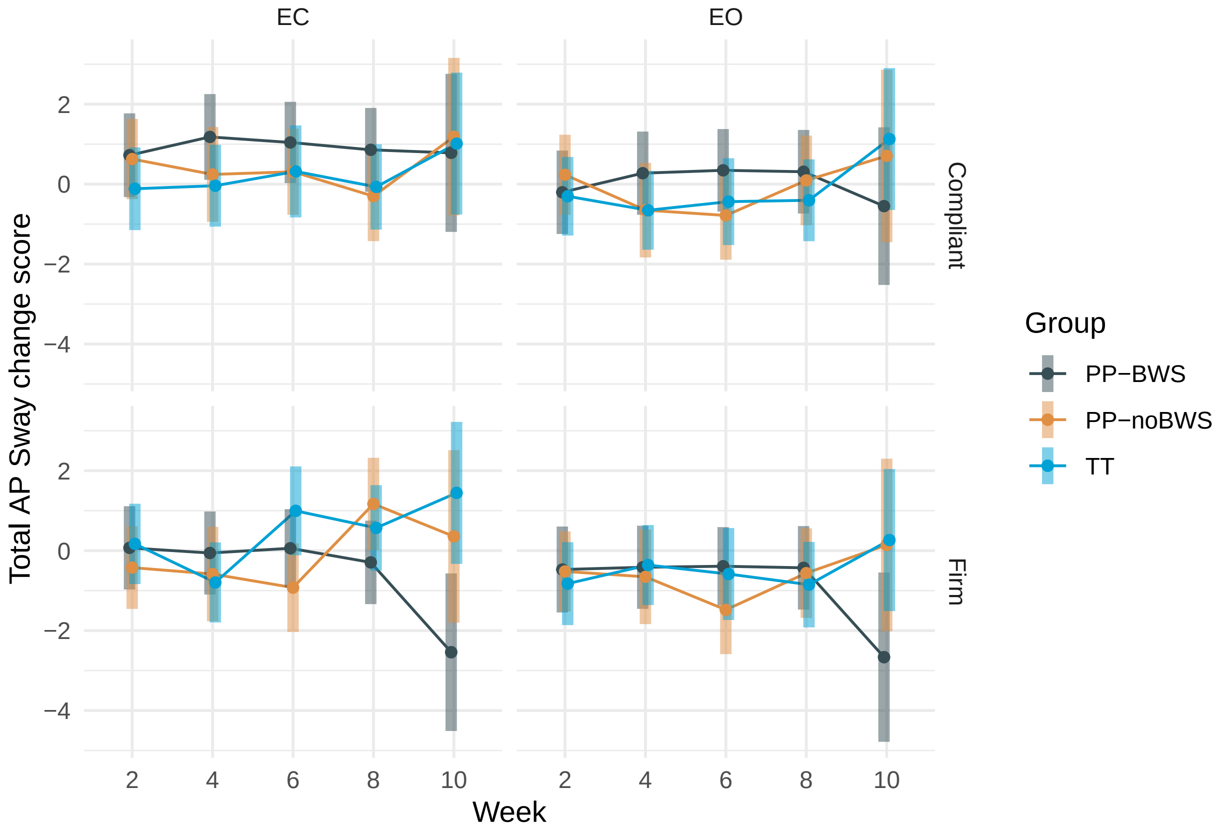

3 Sensitivity Analysis by Multiple-imputations

3.1 Total Distance Wandered

3.1.1 Between Group Difference in Change Score

```
## Registered S3 method overwritten by 'rchiro':
##   method             from
##   is.nan.data.frame mice
```

Contrast	Task	Qual	Week	Difference±SE [95% CI], SMD	t[df], p-value
(PP-BWS) - (PP-noBWS)	Compliant	EC	2	20±20 [-20, 50], 0.13	t[70.1]=1.106, 0.409
(PP-BWS) - TT	Compliant	EC	2	0±20 [-40, 40], -0.03	t[63]=-0.213, 0.832
(PP-noBWS) - TT	Compliant	EC	2	-20±20 [-60, 20], -0.16	t[65.1]=-1.303, 0.409
(PP-BWS) - (PP-noBWS)	Firm	EC	2	20±20 [-20, 60], 0.15	t[52.9]=1.081, 0.542
(PP-BWS) - TT	Firm	EC	2	10±20 [-20, 50], 0.11	t[64.4]=0.92, 0.542
(PP-noBWS) - TT	Firm	EC	2	0±20 [-40, 30], -0.03	t[60]=-0.262, 0.794
(PP-BWS) - (PP-noBWS)	Compliant	EO	2	10±20 [-20, 50], 0.11	t[68.7]=0.943, 0.685
(PP-BWS) - TT	Compliant	EO	2	10±20 [-30, 50], 0.05	t[59.5]=0.407, 0.685
(PP-noBWS) - TT	Compliant	EO	2	-10±20 [-50, 30], -0.07	t[62.4]=-0.518, 0.685
(PP-BWS) - (PP-noBWS)	Firm	EO	2	20±20 [-20, 60], 0.17	t[67.6]=1.385, 0.309
(PP-BWS) - TT	Firm	EO	2	20±20 [-20, 60], 0.16	t[64.6]=1.278, 0.309
(PP-noBWS) - TT	Firm	EO	2	0±20 [-40, 40], -0.01	t[64.6]=-0.103, 0.918
(PP-BWS) - (PP-noBWS)	Compliant	EC	4	10±10 [-30, 50], 0.1	t[44.8]=0.696, 0.735
(PP-BWS) - TT	Compliant	EC	4	0±10 [-30, 30], -0.02	t[79.9]=-0.215, 0.831
(PP-noBWS) - TT	Compliant	EC	4	-10±20 [-50, 30], -0.13	t[34.4]=-0.785, 0.735
(PP-BWS) - (PP-noBWS)	Firm	EC	4	0±20 [-50, 40], -0.04	t[33]=-0.258, 0.95
(PP-BWS) - TT	Firm	EC	4	-10±10 [-30, 20], -0.05	t[101]=-0.502, 0.95
(PP-noBWS) - TT	Firm	EC	4	0±20 [-40, 40], -0.01	t[29.7]=-0.064, 0.95
(PP-BWS) - (PP-noBWS)	Compliant	EO	4	0±20 [-40, 40], -0.01	t[33.5]=-0.081, 0.936
(PP-BWS) - TT	Compliant	EO	4	-10±10 [-30, 20], -0.07	t[95.1]=-0.705, 0.936
(PP-noBWS) - TT	Compliant	EO	4	-10±20 [-50, 40], -0.07	t[27.2]=-0.358, 0.936
(PP-BWS) - (PP-noBWS)	Firm	EO	4	-10±10 [-40, 30], -0.07	t[40.3]=-0.468, 0.907
(PP-BWS) - TT	Firm	EO	4	0±10 [-30, 20], -0.05	t[85.2]=-0.436, 0.907
(PP-noBWS) - TT	Firm	EO	4	0±20 [-40, 50], 0.02	t[25.8]=0.118, 0.907
(PP-BWS) - (PP-noBWS)	Compliant	EC	6	-10±10 [-40, 20], -0.13	t[73.8]=-1.098, 0.667
(PP-BWS) - TT	Compliant	EC	6	0±10 [-40, 30], -0.03	t[46.1]=-0.219, 0.827
(PP-noBWS) - TT	Compliant	EC	6	10±10 [-20, 40], 0.1	t[58.4]=0.769, 0.667
(PP-BWS) - (PP-noBWS)	Firm	EC	6	-10±20 [-50, 30], -0.08	t[35.2]=-0.467, 0.753
(PP-BWS) - TT	Firm	EC	6	-10±20 [-50, 30], -0.12	t[33.1]=-0.702, 0.753
(PP-noBWS) - TT	Firm	EC	6	0±10 [-30, 30], -0.04	t[65.6]=-0.316, 0.753

Contrast	Task	Qual	Week	Difference±SE [95% CI], SMD	t[df], p-value
(PP-BWS) - (PP-noBWS)	Compliant	EO	6	-10±10 [-40, 20], -0.07	t[67.1]=-0.555, 0.892
(PP-BWS) - TT	Compliant	EO	6	-10±10 [-40, 30], -0.05	t[51.5]=-0.393, 0.892
(PP-noBWS) - TT	Compliant	EO	6	0±10 [-30, 30], 0.02	t[62.3]=0.137, 0.892
(PP-BWS) - (PP-noBWS)	Firm	EO	6	-10±20 [-50, 30], -0.1	t[31.3]=-0.573, 0.802
(PP-BWS) - TT	Firm	EO	6	-10±20 [-60, 30], -0.14	t[30.4]=-0.747, 0.802
(PP-noBWS) - TT	Firm	EO	6	0±10 [-30, 30], -0.03	t[76.7]=-0.251, 0.802
(PP-BWS) - (PP-noBWS)	Compliant	EC	8	0±10 [-30, 30], -0.02	t[77.2]=-0.186, 0.853
(PP-BWS) - TT	Compliant	EC	8	10±10 [-20, 40], 0.1	t[88.8]=0.95, 0.517
(PP-noBWS) - TT	Compliant	EC	8	10±10 [-10, 40], 0.12	t[91]=1.169, 0.517
(PP-BWS) - (PP-noBWS)	Firm	EC	8	-20±10 [-50, 20], -0.17	t[45.3]=-1.157, 0.555
(PP-BWS) - TT	Firm	EC	8	-10±10 [-30, 20], -0.07	t[78.8]=-0.593, 0.555
(PP-noBWS) - TT	Firm	EC	8	10±10 [-20, 40], 0.1	t[64.2]=0.797, 0.555
(PP-BWS) - (PP-noBWS)	Compliant	EO	8	-10±10 [-40, 20], -0.08	t[68.7]=-0.664, 0.767
(PP-BWS) - TT	Compliant	EO	8	0±10 [-30, 20], -0.01	t[92.8]=-0.049, 0.961
(PP-noBWS) - TT	Compliant	EO	8	10±10 [-20, 30], 0.08	t[72.4]=0.66, 0.767
(PP-BWS) - (PP-noBWS)	Firm	EO	8	-10±10 [-40, 20], -0.11	t[63.8]=-0.874, 0.71
(PP-BWS) - TT	Firm	EO	8	0±10 [-30, 20], -0.02	t[95.5]=-0.234, 0.816
(PP-noBWS) - TT	Firm	EO	8	10±10 [-20, 40], 0.09	t[71.5]=0.721, 0.71
(PP-BWS) - (PP-noBWS)	Compliant	EC	10	0±20 [-40, 40], 0.01	t[39.9]=0.077, 0.939
(PP-BWS) - TT	Compliant	EC	10	0±20 [-40, 40], -0.02	t[41.3]=-0.149, 0.939
(PP-noBWS) - TT	Compliant	EC	10	0±20 [-40, 40], -0.04	t[39.6]=-0.226, 0.939
(PP-BWS) - (PP-noBWS)	Firm	EC	10	0±20 [-40, 40], -0.01	t[36.5]=-0.084, 0.976
(PP-BWS) - TT	Firm	EC	10	0±20 [-40, 40], -0.01	t[36.3]=-0.055, 0.976
(PP-noBWS) - TT	Firm	EC	10	0±20 [-40, 40], 0.01	t[33.1]=0.03, 0.976
(PP-BWS) - (PP-noBWS)	Compliant	EO	10	0±20 [-40, 40], 0.01	t[43.2]=0.084, 0.984
(PP-BWS) - TT	Compliant	EO	10	0±10 [-30, 30], 0	t[51.2]=-0.02, 0.984
(PP-noBWS) - TT	Compliant	EO	10	0±20 [-40, 40], -0.02	t[34.8]=-0.097, 0.984
(PP-BWS) - (PP-noBWS)	Firm	EO	10	0±10 [-30, 40], 0.04	t[49.4]=0.266, 0.844
(PP-BWS) - TT	Firm	EO	10	10±20 [-30, 50], 0.07	t[35.1]=0.416, 0.844
(PP-noBWS) - TT	Firm	EO	10	0±10 [-30, 40], 0.03	t[44]=0.199, 0.844

3.2 Total ML Sway

3.2.1 Between Group Difference in Change Score

Contrast	Task	Qual	Week	Difference±SE [95% CI], SMD	t[df], p-value
(PP-BWS) - (PP-noBWS)	Compliant	EC	2	2±2 [-3, 6], 0.08	t[104.9]=0.81, 0.714
(PP-BWS) - TT	Compliant	EC	2	1±2 [-4, 5], 0.04	t[90.7]=0.367, 0.714
(PP-noBWS) - TT	Compliant	EC	2	-1±2 [-5, 4], -0.04	t[95.4]=-0.411, 0.714
(PP-BWS) - (PP-noBWS)	Firm	EC	2	2±2 [-3, 6], 0.09	t[100.8]=0.919, 0.667
(PP-BWS) - TT	Firm	EC	2	1±2 [-4, 6], 0.05	t[89.1]=0.466, 0.667
(PP-noBWS) - TT	Firm	EC	2	-1±2 [-6, 4], -0.05	t[88.6]=-0.432, 0.667
(PP-BWS) - (PP-noBWS)	Compliant	EO	2	2±2 [-3, 7], 0.1	t[101]=1.046, 0.618
(PP-BWS) - TT	Compliant	EO	2	1±2 [-4, 6], 0.05	t[92.7]=0.5, 0.618
(PP-noBWS) - TT	Compliant	EO	2	-1±2 [-6, 4], -0.06	t[89.9]=-0.544, 0.618
(PP-BWS) - (PP-noBWS)	Firm	EO	2	1±2 [-3, 6], 0.08	t[104.2]=0.792, 0.828
(PP-BWS) - TT	Firm	EO	2	1±2 [-4, 6], 0.06	t[93.2]=0.56, 0.828
(PP-noBWS) - TT	Firm	EO	2	0±2 [-5, 4], -0.02	t[95.3]=-0.217, 0.828
(PP-BWS) - (PP-noBWS)	Compliant	EC	4	-1±2 [-5, 4], -0.04	t[64.7]=-0.333, 0.992
(PP-BWS) - TT	Compliant	EC	4	-1±1 [-4, 3], -0.04	t[114.7]=-0.412, 0.992
(PP-noBWS) - TT	Compliant	EC	4	0±2 [-5, 5], 0	t[49.1]=-0.011, 0.992
(PP-BWS) - (PP-noBWS)	Firm	EC	4	-1±2 [-6, 3], -0.1	t[61.9]=-0.768, 0.668
(PP-BWS) - TT	Firm	EC	4	-1±1 [-5, 2], -0.09	t[149.9]=-1.067, 0.668
(PP-noBWS) - TT	Firm	EC	4	0±2 [-5, 5], 0	t[55]=-0.026, 0.979
(PP-BWS) - (PP-noBWS)	Compliant	EO	4	-1±2 [-5, 3], -0.06	t[68.3]=-0.474, 0.955
(PP-BWS) - TT	Compliant	EO	4	-1±1 [-4, 2], -0.06	t[132.3]=-0.659, 0.955
(PP-noBWS) - TT	Compliant	EO	4	0±2 [-5, 4], -0.01	t[54.5]=-0.041, 0.967
(PP-BWS) - (PP-noBWS)	Firm	EO	4	-2±2 [-6, 3], -0.11	t[54.3]=-0.813, 0.629
(PP-BWS) - TT	Firm	EO	4	-1±1 [-5, 2], -0.08	t[138]=-0.969, 0.629
(PP-noBWS) - TT	Firm	EO	4	0±2 [-5, 5], 0.02	t[42.1]=0.1, 0.92
(PP-BWS) - (PP-noBWS)	Compliant	EC	6	-2±2 [-5, 2], -0.09	t[153.4]=-1.139, 0.682
(PP-BWS) - TT	Compliant	EC	6	-1±2 [-5, 3], -0.06	t[98.8]=-0.592, 0.682
(PP-noBWS) - TT	Compliant	EC	6	1±2 [-4, 5], 0.04	t[100]=0.411, 0.682
(PP-BWS) - (PP-noBWS)	Firm	EC	6	-2±2 [-6, 2], -0.11	t[100.3]=-1.096, 0.414
(PP-BWS) - TT	Firm	EC	6	-2±2 [-6, 2], -0.12	t[92.5]=-1.132, 0.414
(PP-noBWS) - TT	Firm	EC	6	0±2 [-4, 4], 0	t[156]=-0.053, 0.958
(PP-BWS) - (PP-noBWS)	Compliant	EO	6	-1±2 [-5, 3], -0.06	t[98.4]=-0.582, 0.897
(PP-BWS) - TT	Compliant	EO	6	-1±2 [-5, 3], -0.05	t[102]=-0.475, 0.897
(PP-noBWS) - TT	Compliant	EO	6	0±2 [-4, 4], 0.01	t[91.8]=0.13, 0.897
(PP-BWS) - (PP-noBWS)	Firm	EO	6	-2±2 [-6, 3], -0.11	t[90]=-1.006, 0.475
(PP-BWS) - TT	Firm	EO	6	-2±2 [-7, 3], -0.13	t[70.1]=-1.095, 0.475

Contrast	Task	Qual	Week	Difference±SE [95% CI], SMD	t[df], p-value
(PP-noBWS) - TT	Firm	EO	6	0±2 [-4, 4], -0.02	t[104.7]=-0.183, 0.855
(PP-BWS) - (PP-noBWS)	Compliant	EC	8	-2±2 [-6, 2], -0.14	t[109.1]=-1.415, 0.24
(PP-BWS) - TT	Compliant	EC	8	0±1 [-3, 4], 0.02	t[159.8]=0.233, 0.816
(PP-noBWS) - TT	Compliant	EC	8	3±2 [-1, 6], 0.16	t[105.9]=1.612, 0.24
(PP-BWS) - (PP-noBWS)	Firm	EC	8	-2±2 [-6, 2], -0.13	t[94.6]=-1.237, 0.537
(PP-BWS) - TT	Firm	EC	8	-1±1 [-5, 2], -0.08	t[142.2]=-0.923, 0.537
(PP-noBWS) - TT	Firm	EC	8	1±2 [-3, 4], 0.04	t[96.5]=0.44, 0.661
(PP-BWS) - (PP-noBWS)	Compliant	EO	8	-2±1 [-5, 2], -0.1	t[125.7]=-1.166, 0.646
(PP-BWS) - TT	Compliant	EO	8	-1±1 [-4, 3], -0.03	t[166.7]=-0.444, 0.657
(PP-noBWS) - TT	Compliant	EO	8	1±1 [-2, 5], 0.07	t[120]=0.79, 0.646
(PP-BWS) - (PP-noBWS)	Firm	EO	8	-1±1 [-5, 2], -0.08	t[131.9]=-0.94, 0.733
(PP-BWS) - TT	Firm	EO	8	-1±1 [-4, 2], -0.05	t[168.2]=-0.694, 0.733
(PP-noBWS) - TT	Firm	EO	8	0±1 [-3, 4], 0.03	t[129.4]=0.306, 0.76
(PP-BWS) - (PP-noBWS)	Compliant	EC	10	1±2 [-5, 6], 0.05	t[44.9]=0.302, 0.959
(PP-BWS) - TT	Compliant	EC	10	1±2 [-4, 6], 0.05	t[55.1]=0.376, 0.959
(PP-noBWS) - TT	Compliant	EC	10	0±2 [-5, 5], 0.01	t[60]=0.051, 0.959
(PP-BWS) - (PP-noBWS)	Firm	EC	10	0±2 [-6, 6], -0.01	t[39]=-0.051, 0.96
(PP-BWS) - TT	Firm	EC	10	0±2 [-5, 6], 0.02	t[43]=0.137, 0.96
(PP-noBWS) - TT	Firm	EC	10	0±2 [-5, 6], 0.03	t[46]=0.198, 0.96
(PP-BWS) - (PP-noBWS)	Compliant	EO	10	0±3 [-7, 6], -0.02	t[28.6]=-0.113, 0.998
(PP-BWS) - TT	Compliant	EO	10	0±2 [-6, 5], -0.02	t[38.7]=-0.137, 0.998
(PP-noBWS) - TT	Compliant	EO	10	0±3 [-7, 7], 0	t[27.4]=-0.002, 0.998
(PP-BWS) - (PP-noBWS)	Firm	EO	10	0±2 [-5, 6], 0.02	t[37.7]=0.116, 0.961
(PP-BWS) - TT	Firm	EO	10	0±2 [-6, 6], 0.03	t[32.8]=0.167, 0.961
(PP-noBWS) - TT	Firm	EO	10	0±3 [-7, 7], 0.01	t[24.9]=0.05, 0.961
