# Supplementary material for: Comparative effectiveness of lower body positive pressure and traditional treadmill training on adults with mild balance impairment
Source: Front Aging. 2025 Oct 22;6:1645026. doi: 10.3389/fragi.2025.1645026 (PMC12586058; doi:10.3389/fragi.2025.1645026)
Supplement: Supplementary file 4 [file DataSheet1.pdf]

Table 1S. Between-group differences

**Outcome measures**	**ANOVA**		**Week 2**	**Week 4**	**Week 6**	**Post intervention (Week 8)**	**Follow up (Week 10)**
BBS	**Time effect p<0.000**<br>Group effect NS<br>**Group by time p=0.003**	PP-BWS minus TT	NS	NS	NS	NS	**Significant Favouring TT**
		PP-BWS minus PP-noBWS	NS	NS	NS	NS	**Significant Favouring LBP-noBWS**
		PP-noBWS minus TT	NS	NS	NS	NS	NS
FRT	**Time effect p=0.04**<br>Group effect NS<br>Group by time NS	PP-BWS minus TT	NS	NS	NS	NS	NS
		PP-BWS minus PP-noBWS	NS	NS	NS	NS	NS
		PP-noBWS minus TT	NS	NS	NS	NS	NS
TUG	Time effect NS<br>**Group effect p=0.003**<br>**Group by time p=0.028**	PP-BWS minus TT	NS	**Significant favouring LBP-BWS**	NS	NS	NS
		PP-BWS minus PP-noBWS	**Significant favouring LBP-BWS**	**Significant favouring LBP-BWS**	NS	NS	NS
		PP-noBWS minus TT	NS	NS	NS	NS	NS
Total distance wandered	Time effect NS<br>Group effect NS<br>**Group by time p<0.01**<br>**Group by time by condition P=0.007**	PP-BWS minus TT	NS	NS	NS	NS	NS
		PP-BWS minus PP-noBWS	NS	NS	NS	NS	NS
		PP-noBWS minus TT	NS	Compliant EC<br>**Significant favoring LBPP-noBWS**	NS	NS	NS
MLsway	**Time effect p=0.02**<br>Group effect NS	PP-BWS minus TT	NS	Firm EC and Firm EO<br>**Significant favouring LBPP-BWS**	NS	NS	Compliant EC<br>**Significant favouring TT**

	**Group by time p<0.000**<br>Group by time by condition NS	PP-BWS minus PP-noBWS	NS	NS	Compliant EC<br>**Significant favouring LBPP-BWS**	Compliant EC and Firm EC<br>**Significant favouring LBPP-BWS**	Compliant EC<br>**Significant favouring LBPP-noBWS**
		PP-noBWS minus TT	NS	NS	NS	Compliant EC<br>**Significant favouring TT**	NS
AP sway	NS	PP-BWS minus TT	NT	NT	NT	NT	NT
		PP-BWS minus PP-noBWS	NT	NT	NT	NT	NT
		PP-noBWS minus TT	NT	NT	NT	NT	NT
G&B (Postural Stability)	**Time effect p=0.02**<br>Group effect NS<br>Group by time NS<br>Group by time by condition NS	PP-BWS minus TT	NS	Firm EO<br>**Significant favouring TT**	NS	NS	NS
		PP-BWS minus PP-noBWS	NS	Firm EO<br>**Significant favouring LBPP-noBWS**	NS	NS	NS
		PP-noBWS minus TT	NS	NS	NS	NS	NS
G&B (Postural Stability Mediolateral)	NS	PP-BWS minus TT	NT	NT	NT	NT	NT
		PP-BWS minus PP-noBWS	NT	NT	NT	NT	NT
		PP-noBWS minus TT	NT	NT	NT	NT	NT
G&B (Postural Stability Anterior-Posterior)	**Time effect p=0.03**<br>Group effect NS<br>Group by time NS<br>Group by time by condition NS	PP-BWS minus TT	NT	NT	NT	NT	NT
		PP-BWS minus PP-noBWS	NT	NT	NT	NT	NT
		PP-noBWS minus TT	NT	NT	NT	NT	NT
G&B (Gait Symmetry)	**Time effect p = 0.03**	PP-BWS minus TT	NT	NT	NT	NT	NT
		PP-BWS minus PP-noBWS	NT	NT	NT	NT	NT

	Group effect NS<br>Group by time NS<br>Group by time by condition NS	PP-noBWS minus TT	NT	NT	NT	NT	NT
G&B (Walking Speed)	**Time effect p 0.04**<br>Group effect NS<br>Group by time NS<br>Group by time by condition NS	PP-BWS minus TT	NT	NT	NT	NT	NT
		PP-BWS minus PP-noBWS	NT	NT	NT	NT	NT
		PP-noBWS minus TT	NT	NT	NT	NT	NT
G&B (Step length)	NS	PP-BWS minus TT	NT	NT	NT	NT	NT
		PP-BWS minus PP-noBWS	NT	NT	NT	NT	NT
		PP-noBWS minus TT	NT	NT	NT	NT	NT
G&B (Step Time)	NS	PP-BWS minus TT	NT	NT	NT	NT	NT
		PP-BWS minus PP-noBWS	NT	NT	NT	NT	NT
		PP-noBWS minus TT	NT	NT	NT	NT	NT

Bold = Statistically significant between-group tests
NS = not significant for between-group comparison
NT = not Tested
Favouring for sway data = less sway in that group
